# District-Level Dengue Early Warning Prediction System in Bangladesh Using Hybrid Explainable AI and Bayesian Deep Learning

**DOI:** 10.3390/tropicalmed11030073

**Published:** 2026-03-05

**Authors:** Md. Abu Bokkor Shiddik, Farzana Zannat Toshi, Sadia Yesmin, Md. Siddikur Rahman

**Affiliations:** Department of Statistics, Begum Rokeya University, Rangpur 5404, Bangladesh; abubokkor.brur60@gmail.com (M.A.B.S.); farzana.stat93@brur.ac.bd (F.Z.T.); sadiayesmin.stat@gmail.com (S.Y.)

**Keywords:** dengue fever, explainable artificial intelligence, spatio-temporal modeling, early warning system, Bangladesh

## Abstract

Dengue is a mosquito-borne viral disease which is predominantly endemic in tropical and subtropical countries. In Bangladesh, 321,179 dengue cases were reported in 2023, followed by 101,214 cases in 2024, which highlights a severe and ongoing public health challenge. Dengue transmission risks are shaped by climatic variability, rapid urbanization, socio-economic vulnerability, and healthcare strain. But existing dengue surveillance models remain limited in their ability to capture district-level disparities in Bangladesh. This study aimed to develop a district-level dengue early warning system that integrates climatic, socio-demographic, economic, healthcare, and environmental determinants to generate accurate and interpretable predictions. We examined dengue cases across all 64 districts in Bangladesh from 2017 to 2024, integrating Directorate General of Health Services (DGHS) case records with climate, socio-demographic, economic, and healthcare indicators. Machine learning and deep learning approaches, including Multi-Layer Perceptron (MLP) and Convolutional Long Short-Term Memory (ConvLSTM), were combined with SHAP (Shapley Additive Explanations)-based explainable artificial intelligence. We also used Bayesian spatio-temporal models to capture spatial clustering, temporal dependence, and the lagged transmission effects of dengue. Dengue outbreaks peaked in September 2023, with Dhaka recording 113,233 cases. DENV-4 (Dengue Virus type 4) emerged in 2022, accounting for 27% of infections in 2023. Climate was the strongest predictor of dengue transmission (humidity SHAP = 0.314; minimum temperature SHAP = 0.146; rainfall RR = 1.303). Poverty (SHAP = 0.193) and healthcare capacity (nursing/midwifery density SHAP = 0.073) mostly contributed to dengue prediction. The MLP model achieved the best yearly performance (accuracy = 0.93; ROC-AUC = 0.99), ConvLSTM was the best model in monthly prediction (recall = 0.88; ROC-AUC = 0.81), and Bayesian BYM2_RW2 with lagged effects improved predictive fit (DIC = 3671.055). Our integrated framework delivers transparent, interpretable predictions and district-level early warnings, supporting adaptive dengue outbreak preparedness and resource allocation in Bangladesh.

## 1. Introduction

Dengue fever (DF) is a mosquito-borne viral disease transmitted by *Aedes* mosquitoes [1,2]. It is currently one of the most serious ongoing public health problems in Bangladesh [3]. Bangladesh faced more than 101,000 dengue cases in 2019 and 69,483 in 2023, reflecting the persistent and cyclical nature of outbreaks in the country [3]. The dengue virus is a member of the *Flaviviridae* family with four serotypes (DENV-1, DENV-2, DENV-3 and DENV-4), all responsible for dengue infection [4,5]. Unpredictable rainfall, long monsoon seasons, rising temperatures, rapid urbanization, poor water management, and inadequate vector control increases the risks of dengue transmission, particularly in densely populated districts in Bangladesh [6].

Traditional statistical and surveillance models have been used to monitor dengue outbreaks. However, these approaches often rely on limited climatic or case-based indicators and lack the ability to integrate diverse ecological, socio-demographic, and healthcare drivers simultaneously [7,8]. Early warning systems (EWSs) of dengue surveillance developed in other countries have demonstrated utility, yet in Bangladesh such integrated systems remain underdeveloped [9,10]. This creates an important research gap: although predictive models exist, few frameworks combine accuracy, transparency and district-level spatio-temporal granularity tailored to the Bangladeshi context [11]. Explainable Artificial Intelligence (XAI) and Bayesian spatio-temporal modeling offers a novel integration by combining predictive performance with interpretability and uncertainty quantification [12]. These hybrid approaches enable stakeholders to understand the relative predictive combination of multiple interacting drivers while maintaining methodological rigor [11,13].

Bayesian hierarchical and spatio-temporal models have been widely applied in much infectious disease research, however their application to dengue forecasting in Bangladesh remains limited [14,15]. Most existing dengue-related studies in Bangladesh emphasize only the climatic covariates, whereas the socio-economic and the healthcare system indicators are less frequently incorporated [16,17]. Furthermore, Bayesian approaches have rarely been extended to comprehensive district-level modeling across all 64 districts or evaluated alongside machine learning and deep learning models to assess complementary strengths. Limited exploration of the lagged transmission effects of dengue also constrains understanding of delayed dengue transmission dynamics and localized clustering [18]. This underutilization highlights the need for an integrated framework that simultaneously incorporates multiple drivers of dengue while quantifying uncertainty, thereby improving both interpretability and the policy-relevant manner.

In this study, we aimed to develop and validate a district-level dengue early warning system (EWS) for Bangladesh by integrating the climatic, socio-demographic, healthcare, and environmental determinants within a unified spatio-temporal modeling framework. We applied machine learning (ML) and deep learning (DL) models to optimize predictive performance across short-term and long-term horizons, while SHAP (Shapley Additive Explanations)-based explainability was used to assess the relative predictive contributions of covariates. Bayesian spatio-temporal modeling was incorporated to capture spatial clustering, temporal dependence, and the lagged transmission effects of dengue, enabling district-level uncertainty-aware predictions. By combining predictive accuracy, interpretability, and spatial structure, this framework seeks to support evidence-based dengue preparedness and strengthen adaptive public health responses in Bangladesh.

## 2. Materials and Methods

### 2.1. Study Area and Data Collection

**Figure 1:**
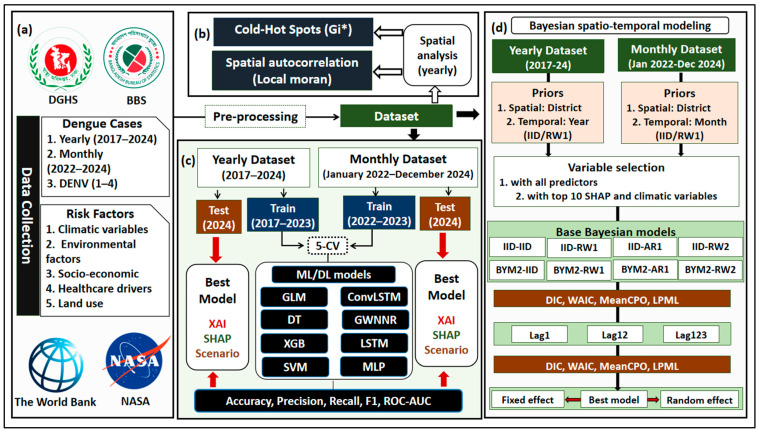
Study framework integrating spatial and spatio-temporal techniques. (**a**) Data Collection, (**b**) spatial analysis of the yearly dataset (2017–2024), (**c**) Building ML\DL models, and (**d**) building Bayesian spatio-temporal models. CV: cross-validation; GLM: Generalized Linear Model; ConvLSTM: Convolutional Long Short-Term Memory (2D); MLP: Multi-Layer Perceptron; LSTM: Long Short-Term Memory (two-sequence); SVM: Support Vector Machine; XGB: eXtreme Gradient Boosting; GWNNR: Geographically Weighted Neural Network Regression; DT: Decision Tree; XAI: Explainable Artificial Intelligence; SHAP: Shapley Additive Explanations; EWS: Early Warning System; IID: Independent and Identically Distributed; RW1: Random Walk (order 1); AR1: Autoregressive (order 1); RW2: Random Walk (order 2); BYM2: Besag–York–Mollié 2 model; DIC: Deviance Information Criterion; WAIC: Watanabe–Akaike Information Criterion; meanCPO: mean Conditional Predictive Ordinate, measuring model predictive performance; LPML: marginal log-likelihood.

This study was conducted across all 64 administrative districts of Bangladesh [19]. We collected dengue case records, including yearly district-wise cases and the dengue virus serotype distribution (DENV1–DENV4) from 2017 to 2024, and monthly cases from January 2022 to December 2024, from the Directorate General of Health Services (DGHS) [20,21]. Climatic and environmental variables were obtained from NASA [22]; socio-demographic, economic and land use indicators were sourced from the World Bank Group [23] and Bangladesh Bureau of Statistics (BBS) [24]; and healthcare system capacity data were obtained from the World Bank [23] and World Health Organization (WHO) [25]. Variables were explicitly grouped into four categories (climate and environmental, socio-demographic and economic, healthcare system capacity, and land use) and incorporated across all models (ML, DL, and Bayesian). Following data collection, we selected the variables a priori based on established dengue transmission frameworks, prior epidemiological evidence, and insights from our previously published studies [11,13,26,27] (see Figure 1 and Supporting Information: Figures S1 and S2; Table S1; Spatio-temporal data selection process).

For predictive modeling, we included all variables from Table S1 in the ML, DL, and Bayesian models. Climate variables included temperature, rainfall, humidity, and surface pressure; socio-demographic and economic variables included GDP, population, literacy, household size, electricity access, and poverty; healthcare variables included hospital beds, physicians, health expenditure, nursing personnel, and UHC coverage; land use variables included arable and agricultural land.

### 2.2. Pre-Processing and Statistical Analysis

We imputed the missing values using advanced spatio-temporal and machine learning techniques to ensure continuity in the district-level datasets [28,29,30,31,32] (see Supporting Information: Missing data imputation strategy). Outliers were detected by using Mahalanobis distances, and we Winsorized the extreme values to the 99th percentile to reduce distortion in the analysis [33]. Multicollinearity among predictors was assessed using the Variance Inflation Factor (VIF), and we removed the variables with VIF > 10 to improve model stability. We also used the Getis-Ord Gi* statistic, which allowed us to detect hot and cold spots at confidence levels of 99%, 95%, and 90% to identify spatial clustering of dengue cases [34]. Spatial autocorrelation was examined using Local Moran’s I (LISA), and we also evaluated it at 99%, 95%, and 90% confidence intervals (see Figure 1) [35]. We computed spatio-temporally weighted correlations to explore associations between dengue cases and explanatory variables that accounted for both spatial and temporal dependencies in dengue (see Supporting Information: Spatio-temporal weighted correlation analysis) [36]. All spatial analyses were conducted in RStudio (version 4.5.1) [37] using the *sf*, *spdep*, and *dplyr* libraries. Maps were generated using the shapefile ‘bgd_adm_bbs_20201113_SHP (administrative level 2)’, which was downloaded from the BBS [24].

For ML and DL classification, we defined the outbreak cutoff values using a percentile-based approach, whereby the districts with case counts above the mean (50th percentile) were categorized as 1 (outbreak) and those below the mean as 0 (non-outbreak), ensuring a standardized threshold for outbreak severity across datasets. To assess the robustness of outbreak classification, sensitivity analyses were performed using additional percentile-based thresholds (P25, P50/median, P75, P90) and historical baseline values with ± standard deviation (SD) from the mean. The results of these analyses are presented in Table S2. Predictor variables were standardized using the z-score transformation to ensure comparability across scales [38]. All these models were implemented in Python (version 3.12.5, packaged by conda-forge) within JupyterLab [39], which provides a robust computational environment for classification and model evaluation [40,41,42,43,44,45,46,47].

### 2.3. Machine Learning and Deep Learning Model Building, and Model Evaluation

In this study, we applied four machine learning (ML) classification models, (i) eXtreme Gradient Boosting (XGBoost), (ii) Support Vector Machine (SVM), (iii) Logistic Regression (GLM) with ElasticNet, and (iv) Decision Tree (DT), and four deep learning (DL) classification models, (i) Multi-Layer Perceptron (MLP), (ii) Long Short-Term Memory (LSTM), (iii) Convolutional Long Short-Term Memory (ConvLSTM), and (iv) Geographically Weighted Neural Network Regression (GWNNR), to predict dengue outbreaks in Bangladesh. For yearly dengue outbreak prediction, we split the dataset into the training set (2017–2023) and the testing set (2024). And for monthly dengue outbreaks prediction, the training set split covered from January 2022 to December 2023, and the testing set included January to December 2024. To ensure robust and interpretable outbreak prediction, we employed a structured modeling pipeline. Multiple ML and DL classifiers were compared across probability thresholds at both annual and monthly resolutions, allowing the identification of the best-performing model for each dataset. Feature contributions were tracked using SHAP to guide covariate selection for subsequent Bayesian spatio-temporal modeling. This approach balances comprehensive model evaluation with parsimony, supporting the development of a practical early warning system based on reliable predictions.

Model performance was evaluated using accuracy, precision, recall, F1-score, and ROC-AUC, calculated at probability thresholds of 0.1, 0.3, and 0.5. The rationale for selecting these models and thresholds is detailed in Supporting Information: Rationale for model selection and threshold choice. All four categories of predictors (climate, socio-demographic, healthcare, and land use) were included in all ML and DL models, with explicit tracking of which features contributed most to model outputs using SHAP. The best-performing model for each dataset (yearly and monthly) was selected for further XAI analysis [38]. SHAP was employed to compute feature contributions, both overall across all districts of Bangladesh and individually for each district. It allows us to identify the top contributing features for dengue prediction. Scenario analyses [48] were conducted by systematically decreasing or increasing the values of the top contributing features by 50 percent, and the resulting changes in dengue case probabilities were examined to assess model sensitivity. Additionally, based on the monthly prediction models, we developed a prototype early warning system. Warning levels were defined according to the best-performing model’s threshold outputs [49], distinguishing between *high alert* and *no-warning* conditions. This early warning system was further analyzed for the period from January 2025 to December 2026 to evaluate its predictive capacity and practical utility in real-world monitoring.

### 2.4. Cross-Validation and Parametric Tuning

We applied five-fold cross-validation across all the ML and DL models to ensure robust performance and minimize overfitting. MLP was tuned for hidden layer sizes (32–64 units), dropout (0.2–0.3), batch size (16), learning rates (0.001–0.01), and epochs (50–500). LSTM models used a two-timestep sequential design to capture epidemiological continuity, while ConvLSTM reshaped district-level features into spatial grids (latitude/longitude as columns) to jointly model spatial clustering and the temporal progression of dengue outbreaks [50]. Additional tuning details for other candidate models are provided in the Supporting Information: Cross-validation and parametric tuning.

### 2.5. Bayesian Spatio-Temporal Modeling of Dengue Cases

We implemented a Bayesian hierarchical framework to investigate spatio-temporal patterns of dengue across all districts using Integrated Nested Laplace Approximation (INLA) [14,15,18,51] (see Supporting Information: Distributional assessment and likelihood selection). Dengue counts exhibited overdispersion, right skewness, and a moderate proportion of zeros, motivating us to use a Negative Binomial likelihood (see Supporting Information: Figures S3 and S4):

$$Y_{it}\sim \;NB\;(\mu_{it},\;\phi ),\;var\left(Y_{it}\right)=\mu_{it}+\frac{\mu_{it}^{2}}{\phi }$$

The log-linear predictor incorporated covariates from all four categories (climate/environmental, socio-demographic/economic, healthcare, and land use) and spatio-temporal random effects:

$$\log\left(\mu_{it}\right)=\;\beta_{0}+\;\sum_{k=1}^{p}\beta_{k}X_{kit}+\;u_{i}+\;v_{t}+\gamma_{t}$$

where $\beta_{k}\;$ are regression coefficients for covariates $X_{kit}$, $u_{i}$ represents spatially structured random effect (BYM2), $v_{t}$ represents unstructured spatial effects (IID) and $\gamma_{t}$ represents temporal effects (IID, RW1/RW2, or AR1).

Spatial effects under BYM2 were modeled as:

$$u_{i}=\;\sqrt{\phi }u_{i}^{structured}+\;\sqrt{1-\phi }u_{i}^{structured},\;u_{i}^{structured}\;\sim \;CAR(adjacency),$$

while temporal trends included first-order or second-order random walks or autoregressive dependence:

$$\gamma_{t}\;\sim \left\{\begin{array}{cc} RW1(\sigma_{\gamma }^{2}) & \left(first-order\;random\;walk\right)\\ RW2(\sigma_{\gamma }^{2}) & (second-order\;random\;walk)\;\\ AR1(\rho ) & \left(\mathrm{autoregressive}\right) \end{array}\right.$$

Eight combinations of spatial and temporal structures were evaluated using all covariates or subsequently the top 10 features identified via SHAP (see Figure 1 and Supporting Information: Table S3). To account for delayed transmission effects, lagged responses up to three periods (Lag1, Lag12, Lag123) were tested by using the following equation:

$$\log\left(\mu_{it}\right)=\;\beta_{0}+\;\sum_{k=1}^{p}\beta_{k}X_{kit}\;+\;\sum_{l=1}^{L}\delta_{l}Y_{i,\;t-l}+u_{i}+\;v_{i}+\gamma_{t}$$

where $\delta_{l}$ represents the effect of lagged dengue cases $Y_{i,\;t-l}$ for lags *l* = 1, 12, 123.

Model performance was assessed using standard Bayesian criteria: Deviance Information Criterion (DIC), Watanabe–Akaike Information Criterion (WAIC), and Log Pseudo-Marginal Likelihood (LPML). The best model minimized DIC and WAIC while maximizing LPML. Posterior estimates of fixed effects, spatial and temporal random effects, and relative risks were obtained from the best selected model (BYM2_RW1 with Lag123 and top SHAP covariates):

$${RR}_{k}=exp(\sum_{k=1}^{p}\beta_{k}X_{kit}\;+\;\hat{u_{i}}\;+\;\hat{\;v_{i}}+\hat{\gamma_{t}})$$

Posterior predictive distributions were used for validation and forecasting, providing robust inference on the spatio-temporal dynamics of dengue [14,15,18,51] (see Supporting Information: Posterior Analysis).

## 3. Results

### 3.1. Spatio-Temporal Patterns of DF in Bangladesh (2017–2024)

**Figure 2:**
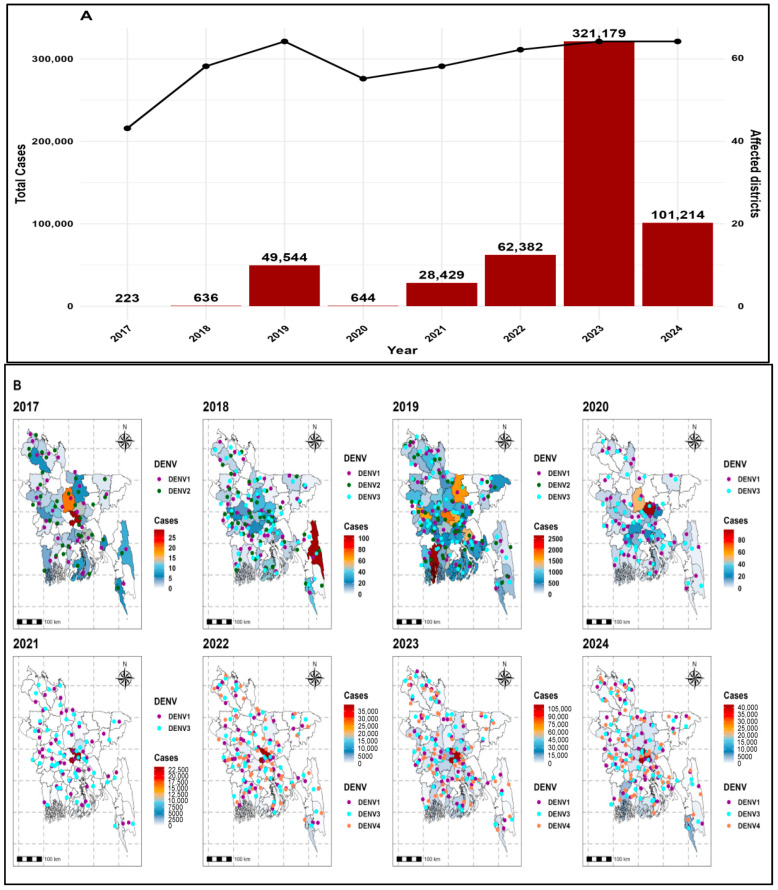
(**A**) Yearly distribution of dengue cases and affected districts across Bangladesh. (**B**) Spatio-temporal patterns of dengue incidence with associated serotype distribution from 2017 to 2024. Maps were generated in RStudio (version 4.5.1) using the sf library and the shapefile bgd_adm_bbs_20201113_SHP (administrative level 2), obtained from the Bangladesh Bureau of Statistics (BBS).

Bangladesh faced the first dengue outbreak in 2019 (49,544 cases), and the largest outbreak was in 2023 (321,179 cases), and in 2024, it still showed a high burden (101,214 cases) (see Figure 2; Supporting Information: Figures S1 and S2; Tables S4 and S5). The disease followed a seasonal pattern, with very few cases in January (only two average cases in January 2022), rising from June and peaking in August and September (Figure 2; Supporting Information: Figures S1 and S2; Tables S6 and S7). Dhaka was the most dengue-affected district (113,233 cases in September 2023) (Figure 2; Tables S6–S9). Hotspot analysis confirmed these outbreak patterns. It identified the district Dhaka as a hotspot in 2020 (GiZ = 3.616, 99% CI) and showed strong clustering of dengue outbreaks in 2017 (LisaZ = 3.654) and 2024 (LisaZ = 4.945) (See Supporting Information: Figure S5; Tables S10–S11). Barishal reported 13,603 cases in 2023, including 8874 in September, while Lakshmipur reported 6950 cases in 2023, including 4446 in September (Figure 2; Tables S6 and S10). Gazipur emerged as a hotspot in 2024 (GiZ = 3.398, 99% CI) and showed strong clustering in 2020 (LisaZ = 6.842, 99% CI). Narayanganj was identified as a hotspot in 2024 (GiZ = 3.146, 99% CI; LisaZ = 3.122, 99% CI) (Figure S5; Tables S10 and S11). Manikganj was identified as a hotspot in 2023 (GiZ = 3.205, 99% CI), while Munshiganj was identified as a hotspot in both 2023 and 2024 (GiZ = 3.019, 99% CI) (See Supporting Information: Figure S5; Table S10).

Virus serotypes shifted across years. DENV-1 remained common every year, accounting for 53% of infections in 2023. DENV-2 disappeared after 2019. DENV-3 was dominant in 2019 (697 average cases) and again in 2023 (3714 average cases). DENV-4 appeared in 2022 and expanded rapidly, making up 27% of infections in 2023 (See Figure 2; Supporting Information: Table S12).

### 3.2. Relationship Between Dengue Cases and Associated Risk Variables

The number of yearly dengue cases in Bangladesh was positively associated with the poverty head-count ratio (r = 0.286, 95% CI 0.205–0.364, *p* < 0.01), population growth (r = 0.218, 95% CI 0.134–0.299, *p* < 0.01), average temperature (r = 0.223, 95% CI 0.139–0.304, *p* < 0.01), and maximum temperature (r = 0.223, 95% CI 0.139–0.304, *p* < 0.01), while minimum temperature exhibited a negative correlation with dengue (r = −0.134, 95% CI −0.218–−0.048, *p* < 0.01).

For monthly dengue cases, significant positive associations with dengue were observed with minimum temperature (r = 0.261, 95% CI 0.2225–0.2987, *p* < 0.01), rainfall (r = 0.290, 95% CI 0.2516–0.3264, *p* < 0.01), relative humidity (r = 0.241, 95% CI 0.2026–0.2795, *p* < 0.01), and domestic general government health expenditure (r = 0.099, 95% CI 0.0582–0.1391, *p* < 0.01). Negative correlations with dengue were identified for arable land (r = −0.059, 95% CI −0.1–−0.0186, *p* < 0.01) and agriculture land (r = −0.059, 95% CI −0.1–−0.0186, *p* < 0.01) (see Supporting Information: Figure S6; Tables S13 and S14).

### 3.3. Selecting Best Performance Models for Dengue Prediction

#### 3.3.1. Machine Learning and Deep Learning Model Selection

**Figure 3:**
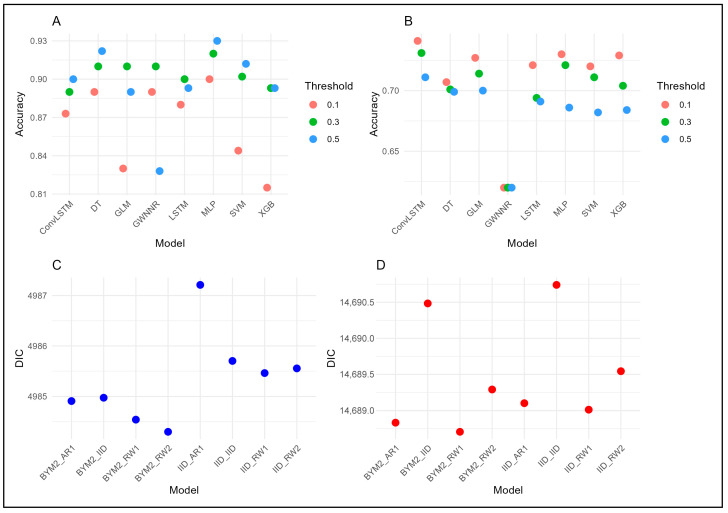
Machine learning and deep learning model comparison of test set for (**A**) yearly dengue dataset, (**B**) monthly dengue dataset. Bayesian spatio-temporal model comparison for (**C**) yearly dengue and (**D**) monthly dataset; GLM: Generalized Linear Model; ConvLSTM: Convolutional Long Short-Term Memory (2D); MLP: Multi-Layer Perceptron; LSTM: Long Short-Term Memory (two-sequence); SVM: Support Vector Machine; XGB: eXtreme Gradient Boosting; GWNNR: Geographically Weighted Neural Network Regression; DT: Decision Tree; IID: Independent and Identically Distributed; RW1: Random Walk (order 1); AR1: Autoregressive (order 1); RW2: Random Walk (order 2); BYM2: Besag–York–Mollié 2 model.

For yearly dengue cases, the MLP achieved the strongest performance at the 0.5 threshold, with training accuracy (0.980), ROC-AUC (0.991), test accuracy (0.930), and F1 score (0.902) (Figure 3; Table S15). For the monthly cases dataset, ConvLSTM performed best at the 0.3 threshold, with recall (0.960 train; 0.881 test) and ROC-AUC (0.921 train; 0.810 test), showing superior ability to capture short-term temporal patterns compared to XGB, MLP, and LSTM (See Figure 3 and Supporting Information: Figure S7; Table S16).

#### 3.3.2. Bayesian Spatio-Temporal Model Selection

Spatio-temporal comparisons indicated that BYM2 models with RW2 temporal effects outperformed IID models. For yearly data, BYM2_RW2 with top 10 variables achieved a DIC (4983.317), WAIC (4991.185), and log-likelihood (−2564.923), with a further improvement using 1–3-month lags (DIC 3671.055, WAIC 3675.390) (Table S17). For monthly data, BYM2_RW2 and BYM2_RW1 showed a lower DIC (14,686–14,687), WAIC (14,697–14,698), and higher log-likelihood (−7519 to −7516), with lagged models again improving predictive performance (see Figure 3 and Figure S7 and Supporting Information: Table S18).

### 3.4. Key Predictive Features on Dengue Outbreak Prediction in Bangladesh

**Figure 4:**
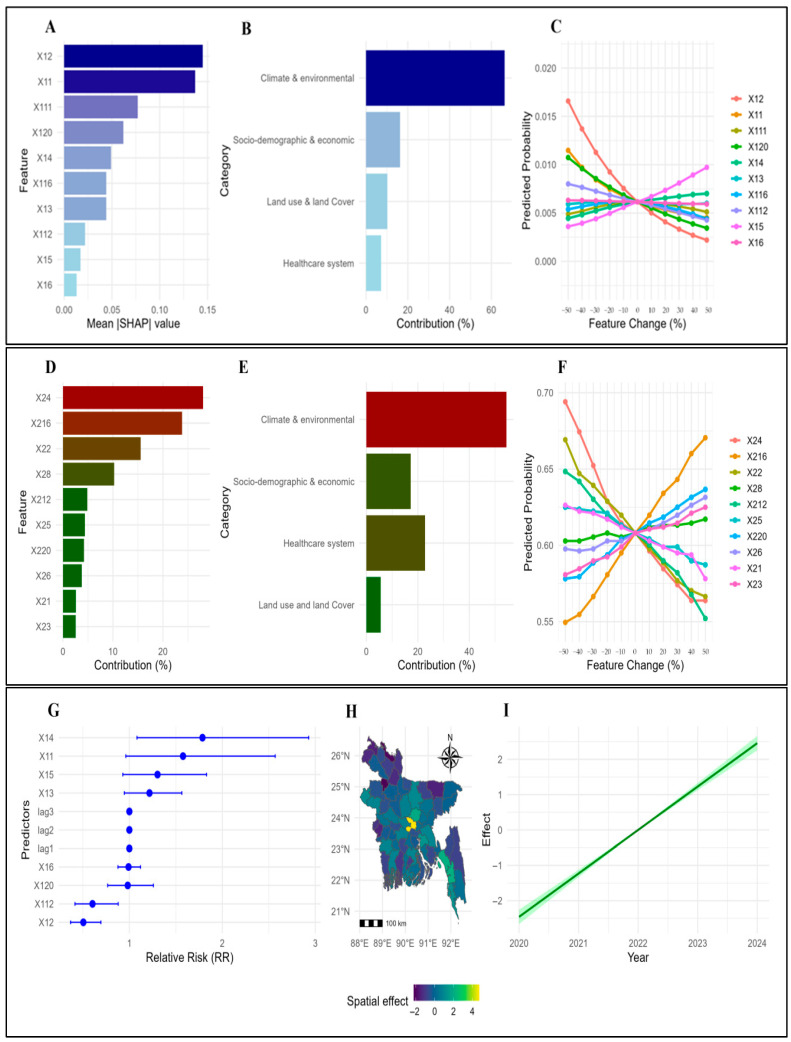

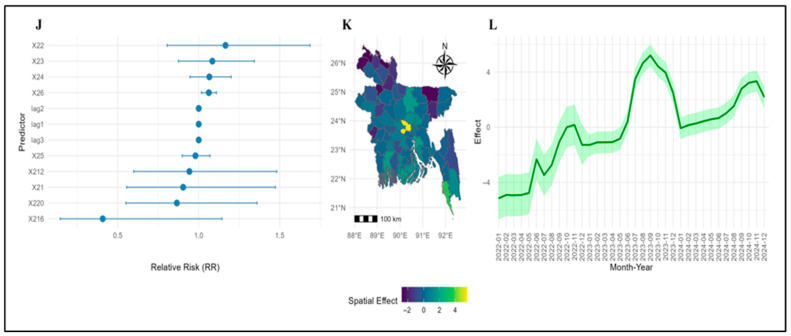
(**A**) SHAP analysis identifying the top 10 contributing features for yearly dengue case prediction. (**B**) Aggregated SHAP contributions by category for yearly dengue case prediction. (**C**) Sensitivity analysis of top 10 yearly predictors. (**D**) SHAP analysis identifying the top 10 contributing features for monthly dengue case prediction. (**E**) Aggregated SHAP contributions by category for monthly dengue case prediction. (**F**) Sensitivity analysis of top 10 monthly predictors. (**G**) Fixed effect on yearly dengue cases, (**H**) spatial random effect of yearly cases. (**I**) Yearly temporal effect of yearly dengue cases. (**J**) Fixed effect on yearly dengue cases, (**K**) spatial random effect of yearly cases. (**L**) Yearly temporal effect of yearly dengue cases; X12: Minimum yearly temperature at 2 m (°C); X11: Yearly average temperature at 2 m (°C); X111: Yearly population growth (%); X120: Yearly arable land; X14: Yearly relative humidity at 2 m (%); X13: Maximum yearly temperature at 2 m (°C); X116: Yearly domestic general government health expenditure (%); X112: Yearly average household size; X15: Yearly rainfall corrected (mm/day); X16: Yearly surface pressure (kPa); X24: Monthly relative humidity at 3 m (%); X216: Monthly domestic general government health expenditure (%); X22: Minimum monthly temperature at 3 m (°C); X28: Total monthly population (each district); X212: Average monthly household size; X25: Monthly rainfall corrected (mm/day); X220: Monthly arable land; X26: Monthly surface pressure (kPa); X21: Monthly average temperature at 2 m (°C); X23: Maximum monthly temperature at 3 m (°C).

Climatic and environmental factors were the most contributing predictors in the modeling of dengue (contributing 66.35% (yearly) and 54.44% (monthly). Socio-demographic and economic indicators ranked second (contribution on dengue outbreak prediction: 16.27% (yearly) and 17.21% (monthly)). Healthcare system capacity explained 7.17% (yearly) but played a stronger role in monthly prediction with 22.79% (monthly). Land use and land cover contributed 10.21% (yearly) and 5.56% (monthly). These values highlight the dominant role of the climate, with healthcare, socio-economic, and land use factors providing additional context for district-level heterogeneity (See Figure 4; Supporting Information: Figure S8; Tables S18 and S19).

#### 3.4.1. Climatic and Environmental Factors on Dengue Outbreak Prediction

Climate was the strongest predictor in the model for dengue. Yearly SHAP contributions were highest in Mymensingh (76.29%), Bagerhat (73.63%), Bandarban (73.20%), and Patuakhali (73.87%). The minimum yearly temperature was negatively associated with dengue risk (RR = 0.505), while the average temperature (RR = 1.577), maximum temperature (RR = 1.217), relative humidity (RR = 1.787), and rainfall (RR = 1.303) increased the risk. Monthly predictors confirmed climate dominance, with relative humidity (SHAP = 0.314) and minimum temperature (SHAP = 0.146) as the strongest predictors of dengue. Scenario analysis showed outbreak probabilities shifting between 0.573 and 0.636 when humidity and temperature varied ±50% (See Figure 4; Supporting Information: Figure S8; Tables S20–S34).

#### 3.4.2. Socio-Demographic and Economic Indicators of Dengue Outbreak Prediction

Socio-economic factors had important predictive contributions across both scales. Yearly SHAP contributions were particularly high in Moulvibazar (30.38%), Habiganj (29.95%), Cox’s Bazar (20.33%), and Chattogram (19.36%). The average household size was negatively associated with dengue predictions (RR = 0.604). Monthly predictors showed socio-demographic indicators contributing 17.21%, reflecting population density and household characteristics as important secondary predictors of transmission (See Figure 4; Supporting Information: Figure S8; Tables S19–S33).

#### 3.4.3. Healthcare System Capacity on Dengue Outbreak Prediction

**Figure 5:**
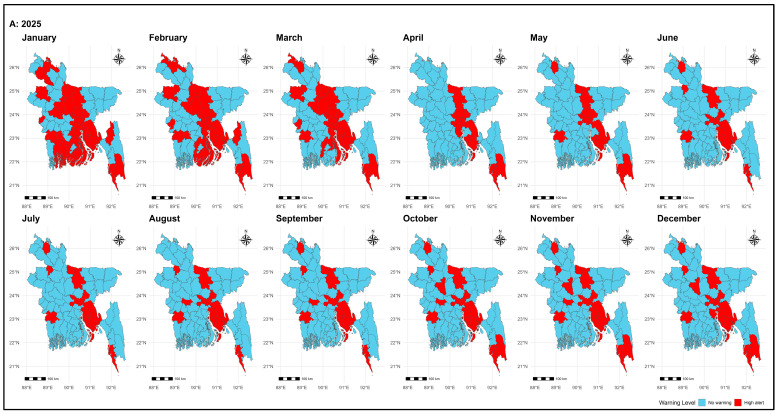

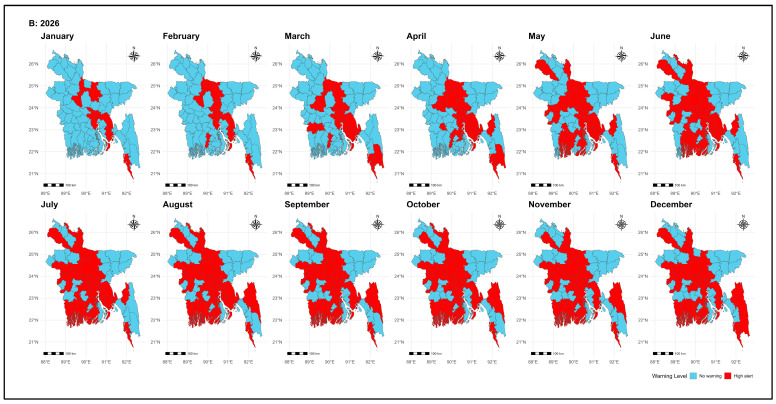
District-wise monthly early warnings for dengue outbreaks by using ConvLSTM (**A**) for the year 2025 and (**B**) for the year 2026. Maps were generated in RStudio (version 4.5.1) using the sf library and the shapefile bgd_adm_bbs_20201113_SHP (administrative level 2), obtained from the Bangladesh Bureau of Statistics (BBS).

Healthcare resources made a modest contribution to yearly dengue outbreak predictions but were more influential in monthly dengue predictions. In Dhaka, hospital beds (SHAP = 27.4%) and physicians (SHAP = 27.9%) together explained over 54% of yearly predicted dengue risk, while in Chattogram healthcare indicators contributed 43.8% to dengue prediction. Monthly predictors showed healthcare capacity contributing 22.79% to dengue prediction, with government health expenditure (SHAP = 0.112) and nursing/midwifery density (SHAP = 0.096) influencing transmission. These findings emphasize the role of healthcare infrastructure in shaping model-predicted outbreak patterns (See Figure 4 and Figure 5; Supporting Information: Tables S19–S33).

#### 3.4.4. Land Use and Land Cover on Dengue Outbreak Prediction

Land use indicators made localized predictive contributions to dengue outbreaks. Yearly SHAP contributions on dengue prediction were highest in Rangamati (40.4%), Bandarban (28.2%), Chapai Nawabganj (16.5%), and Chuadanga (18.6%), though Bayesian analysis indicated minor direct effects (RR = 0.982). Monthly predictors showed smaller overall predictive contributions (5.56%) on dengue, but localized contributions were notable in Narayanganj (12.2%) and Rangamati (9.2%). These results suggest that land use patterns, including arable land and urbanization, influence dengue outbreak predictions across districts (See Figure 4; Supporting Information: Figure S8; Tables S19–S33).

### 3.5. District-Wise Monthly Early Warning for Dengue Outbreak (2025–2026)

The ConvLSTM model shows a very clear picture of dengue risk across Bangladesh. In both 2025 and 2026, the urban and coastal centers (Dhaka, Chattogram, Cox’s Bazar, Cumilla, Mymensingh, Narayanganj, and Noakhali) stayed under continuous *high alert*. On the other hand, districts such as Habiganj, Sunamganj, Sylhet, Magura, Naogaon, Rajbari, Panchagarh, Gopalganj, and Rangpur remained under consistently low alert across both years. Between these extremes, many districts showed seasonal or progressive alerts. Bogura, Dinajpur, and Gazipur started with low probabilities early in the year but rose sharply to above 0.95 later, triggering sustained alerts. Districts like Netrokona, Sirajganj, Tangail, Pirojpur, Patuakhali, Khagrachhari, Jhenaidah, and Feni also peaked above 0.9 during outbreak months. Lakshmipur and Narail showed sharp peaks followed by declines, indicating short but intense outbreak periods (See Figure 5 and Supporting Information: Table S35).

## 4. Discussion

Our findings demonstrate that the dengue transmission in Bangladesh is shaped primarily by climate, with rainfall, humidity and temperature consistently emerging as the strongest predictors of dengue outbreaks. Seasonal dengue peaks occur in August and September, coinciding with monsoon rainfall. This pattern confirms that the climate plays a dominant role in dengue outbreaks and it also is consistent with observations from Brazil, Thailand and Singapore [6]. Scenario analyses further showed that even modest changes in humidity or temperature could shift dengue outbreak probabilities in meaningful ways. These findings underscore the sensitivity of dengue transmission to climatic variability in Bangladesh [52].

However, climate alone does not explain the magnitude of dengue outbreaks in Bangladesh. Socio-economic vulnerability and healthcare capacity also amplify the dengue risks. Poverty, population growth, and limited access to healthcare were positively associated with dengue, with Dhaka, due to its dense population and overstretched infrastructure, emerging as the most dengue-affected district [53,54]. These systemic vulnerabilities sustain dengue transmission over time and explain persistent high-dengue-risk districts.

The MLP model captured the long-term structural dengue-related drivers (poverty and healthcare expenditure), and ConvLSTM excelled at detecting the short-term climatic variability and spatial clustering of dengue. This dual perspective is crucial: one model explains systemic vulnerabilities that sustain the dengue transmission year after year, and the other provides time-sensitive alerts for the immediate dengue outbreak response [55]. Bayesian spatio-temporal analysis confirmed all these findings. BYM2 models with RW1/RW2 temporal structures consistently outperformed simpler IID models, showing strong spatial dengue clustering and temporal dengue dependence [56]. Incorporating the lagged responses improved predictive performance, reflecting delayed dengue transmission effects [8]. Posterior estimates reinforced the dominance of the climate variables, while also showing how socio-economic and healthcare indicators explain district-level dengue heterogeneity. This convergence across modeling approaches strengthens confidence in the robustness of our findings [57].

The dengue serotype shifts we documented, particularly the rapid rise in DENV-4 after 2022, and the emergence of new hotspots in Gazipur, Narayanganj, and Munshiganj, highlight the need for adaptive dengue surveillance [58].

Our ConvLSTM-based early warning system flagged districts under continuously high alert such as Dhaka, Chattogram, Cox’s Bazar, and Narayanganj. And the MLP and Bayesian models pointed to socio-economic and healthcare gaps sustaining long-term dengue transmission. While the system shows high predictive accuracy, its real-world implementation in Bangladesh requires timely, high-quality data, regular updates, and coordination among health authorities. Reporting delays or under-ascertainment could affect operational performance. Nevertheless, the framework can provide retrospective insights for planning and prospective guidance for resource allocation in high-risk districts [59].

By integrating XAI with SHAP and Bayesian inference, we moved beyond “black box” predictions. SHAP clarified the relative importance of climate, socio-economic, healthcare, and land-use drivers, while Bayesian modeling quantified uncertainty and the relative risks of dengue [60]. This combination makes predictions both accurate and interpretable, a crucial step for real-world decision-making [11,14,15,56,57].

### Limitations

Our study has some limitations. Firstly, we collected the dengue cases data from official surveillance records, which may underreport some infections (particularly in rural districts with weaker health infrastructure). Secondly, we aggregated climatic and socio-economic indicators at the district level, which may obscure local heterogeneity. For predictive models to be more useful for public health decision-making, a higher geographic resolution beyond the district level would be beneficial, as it could allow control activities to be targeted more precisely and potentially reduce transmission intensity. Thirdly, model-specific limitations also exist. The MLP is strong for yearly prediction, but it may oversimplify temporal dynamics and is sensitive to overfitting when data are limited. ConvLSTM is also effective for monthly predictions, but it requires large datasets and high computational resources, which may restrict real-time deployment. Both models depend heavily on input quality and cannot fully capture unmeasured behavioral, ecological, or vector-control interventions. Finally, the validation and generalizability of our models are limited. Their evaluation relied primarily on train–test splits within the same country and surveillance system, and no external or quasi-external validation was performed. This may lead to optimistic performance estimates. The retrospective validation may not fully reflect operational challenges in a real-world early warning system, and predictive performance could vary under reporting delays or under-ascertainment. While our framework provides valuable insights, prospective or real-time implementation would require careful consideration of data quality, timeliness, and integration with public health workflows.

## 5. Conclusions

Dengue fever in Bangladesh has escalated over the past decade. It is driven primarily by climatic variability and increased by socio-economic vulnerability and healthcare constraints. Our study developed a spatio-temporal framework that integrates XAI with Bayesian modeling to deliver dengue predictions that are both accurate and interpretable. The MLP model captured long-term structural dengue-related drivers (poverty, health expenditure, and healthcare capacity), while the ConvLSTM model excelled in short-term dengue prediction by detecting climatic variability and spatial clustering. The Bayesian BYM2 models further validated the spatial clustering, temporal dependence, and lagged dengue transmission effects. It strengthens confidence in the robustness of our overall approach. Together, these models provide complementary insights into yearly and monthly dengue risks, which enables district-level early warning and resource prioritization. By embedding SHAP-based interpretability and spatial statistics, our framework moves beyond “black box” predictions and offers actionable knowledge for policymakers. Future research should emphasize real-time deployment, the integration of behavioral and ecological data, and incorporation of vector-control interventions. Expanding this system to multi-disease surveillance and climate change projections will further enhance national preparedness and adaptive health planning against evolving vector-borne threats.

## Data Availability

The study data and codes can be accessed upon request to the corresponding author (M.S.R.).

## Supplementary Materials

The following supporting information can be downloaded at: https://www.mdpi.com/article/10.3390/tropicalmed11030073/s1, 1. Spatio-temporal data selection process. 2. Missing data imputation strategy. 3. Spatio-temporal weighted correlation analysis. 4. Rationale for machine learning and deep learning model selection and threshold choice. 5. Cross-validation and parametric tuning. 6. Distributional assessment and likelihood selection. 7. Bayesian spatio-temporal modeling of dengue incidence. Figure S1. All Bangladesh’s districts with dengue cases during the study period (2017–2024). Sixty-four dengue-affected districts are shown. Year to year, the number of dengue cases per district varies significantly. The bubble size represents the number of dengue cases per year per district. The bigger the bubble size, the greater the number of cases in a district per year. Figure S2. All Bangladesh’s districts with dengue cases during the study period (2022–2024). Sixty-four dengue-affected districts are shown. Month to month, the number of dengue cases per district varies significantly. The bubble size represents the number of dengue cases per year per district. The bigger the bubble size, the greater the number of cases in a district per year. Figure S3. Distribution of yearly dengue cases in Bangladesh. Panel (A) shows the histogram of raw yearly cases, (B) shows the log-transformed distribution, and (C) compares zero versus non-zero cases. Figure S4. Distribution of monthly dengue cases in Bangladesh. Panel (A) shows the histogram of raw monthly cases, (B) shows the log-transformed distribution, and (C) compares zero versus non-zero cases. Figure S5. (A) Hotspot and cold spot analysis of dengue cases using Getis-Ord Gi*, highlighting statistically significant clusters of elevated and reduced transmission risk across districts from 2017 to 2024 and (B) spatial autocorrelation and clustering analysis based on Local Moran’s I (LISA), showing the strength and direction of spatial dependence with confidence intervals at 90%, 95%, and 99%, thereby identifying areas of persistent transmission and emerging outbreak zones. Maps were generated in RStudio (version 4.5.1) using the sf library and the shapefile bgd_adm_bbs_20201113_SHP (administrative level 2), obtained from the Bangladesh Bureau of Statistics (BBS). Figure S6. Significant spatio-temporal weighted correlations between (A) yearly dengue cases, (B) monthly cases and the associated variables. X11: Yearly average temperature at 2 m (°C); X12: Minimum yearly temperature at 2 m (°C); X13: Maximum yearly temperature at 2 m (°C); X14: Yearly relative humidity at 2 m (%); X15: Yearly rainfall corrected (mm/day); X16: Yearly surface pressure (kPa); X19: Yearly poverty head-count ratio (% of population); X111: Yearly population growth (%); X112: Yearly average household size; X116: Yearly domestic general government health expenditure (%); X118: Yearly UHC Service Coverage Index (SDG 3.8.1); X120: Yearly arable land; X121: Yearly agriculture land; X21: Monthly average temperature at 2 m (°C); X22: Minimum monthly temperature at 2 m (°C); X23: Maximum monthly temperature at 2 m (°C); X24: Monthly relative humidity at 2 m (%); X25: Monthly rainfall corrected (mm/day); X26: Monthly surface pressure (kPa); X27: Monthly GDP (billion US$); X28: Total monthly population (each district); X29: Monthly poverty head-count ratio (% of population); X210: Monthly adult literacy rate (%); X211: Monthly population growth (%); X212: Average monthly household size; X213: Monthly access to electricity (% of population); X214: Total number of hospital beds (monthly); X215: Number of physicians (monthly); X216: Monthly domestic general government health expenditure (%); X217: Monthly density of nursing and midwifery personnel (per 10,000 population); X218: Monthly UHC Service Coverage Index (SDG 3.8.1); X219: Monthly land use; X220: Arable land (monthly); X221: Agriculture land (monthly). Figure S7. Model comparison matrices. Machine learning and deep learning model comparison for (A) yearly dengue dataset, (B) monthly dengue dataset. Bayesian spatio-temporal model comparison for (C) yearly dengue and (D) monthly dataset; GLM: Generalized Linear Model; ConvLSTM: Convolutional Long Short-Term Memory (2D); MLP: Multi-Layer Perceptron; LSTM: Long Short-Term Memory (two-sequence); SVM: Support Vector Machine; XGB: eXtreme Gradient Boosting; GWNNR: Geographically Weighted Neural Network Regression; DT: Decision Tree; IID: Independent and Identically Distributed; RW1: Random Walk (order 1); AR1: Autoregressive (order 1); RW2: Random Walk (order 2); BYM2: Besag–York–Mollié 2 model. Figure S8. (A) District-wise SHAP analysis identifying the top 10 contributing features for yearly dengue case prediction. (B) District-wise aggregated SHAP contributions by category for yearly dengue case prediction. (C) District-wise SHAP analysis identifying the top 10 contributing features for monthly dengue case prediction. (D) District-wise aggregated SHAP contributions by category for monthly dengue case prediction; X12: Minimum yearly temperature at 2 m (C); X11: Yearly average temperature at 2 m (C); X111: Yearly population growth(%); X120: Yearly arable land; X14: Yearly relative humidity at 2 m (%); X13: Maximum yearly temperature at 2 m (C); X116: Yearly domestic general government health expenditure (%); X112: Yearly average household size; X15: Yearly rainfall corrected (mm/day); X16: Yearly surface pressure (kPa); X24: Monthly relative humidity at 3 m (%); X216: Monthly domestic general government health expenditure (%); X22: Minimum monthly temperature at 3 m (C); X28: Total monthly population (each district); X212: Average monthly household size; X25: Monthly rainfall corrected (mm/day); X220: Monthly arable land; X26: Monthly surface pressure (kPa); X21: Monthly average temperature at 2 m (C); X23: Maximum monthly temperature at 2 m (°C). Table S1. Description of the variables used in our study. Table S2. Sensitivity analysis of dengue outbreak prediction using different cutoffs and probability thresholds. Table S3. The description of the Bayesian spatio-temporal models used in our study. Table S4. Summary statistics of yearly dengue cases over 64 districts in Bangladesh from 2017 to 2024. Table S5. Yearly total aggregated dengue cases and total affected districts of Bangladesh. Table S6. Monthly dengue case summary from January 2022 to December 2024. Table S7. Summary of monthly dengue cases in Bangladesh from 2022 to 2024. Table S8. District-wise summary of yearly dengue cases with highest year and lowest affected year. Table S9. District-wise summary of monthly dengue cases from 2022 to 2024 in Bangladesh. Table S10. Cold spot and hotspot analysis of yearly dengue cases in Bangladesh from 2017 to 2024. Table S11. Spatial autocorrelation or cluster analysis of yearly dengue cases in Bangladesh from 2017 to 2024. Table S12. DENV distribution summary from 2017 to 2024 in Bangladesh. Table S13. ST-weighted correlation between yearly dengue cases and the associated variables. Table S14. ST-weighted correlation between monthly dengue cases and the associated variables. Table S15. Performance matrices of yearly dengue cases 2017–24 (training set 2017–2023 and test set 2024). Table S16. Performance matrices of monthly dengue cases 2022–24 (training set January 2022–December 2023 and test set January 2024 to December 2024). Table S17. Spatio-temporal model comparison metrics for yearly dengue cases in Bangladesh (DIC, WAIC, meanCPO, and marginal log-likelihood). Table S18. Spatio-temporal model comparison metrics for monthly dengue cases using all variables and top 10 selected variables. Table S19. Category-wise summed contribution on yearly dengue case prediction. Table S20. Category-wise summed contribution on monthly dengue case prediction. Table S21. Top 10 features to predict yearly dengue cases in Bangladesh. Table S22. Scenario analysis of the top 10 features on yearly dengue case prediction. Table S23. District-wise SHAP analysis for selection of top 10 contributing features on yearly dengue case prediction. Table S24. District-wise total aggregated SHAP contribution of different categories on yearly dengue case prediction. Table S25. Posterior estimates of fixed effects for the BYM2_RW2_Lag123 model using standardized yearly predictors. Table S26. Posterior estimates of random effects for the BYM2_RW2_Lag123 model using standardized yearly predictors. Table S27. Posterior estimates of temporal effects for the BYM2_RW2_Lag123 model using standardized yearly predictors. Table S28. Top 10 contribution of the factors on the prediction of monthly dengue cases. Table S29. Top 10 contribution of the factors on the prediction of district-wise monthly dengue cases. Table S30. Top contribution of categories on the prediction of district-wise monthly dengue cases. Table S31. Scenario analysis of the top 10 features on monthly dengue prediction. Table S32. Posterior estimates of fixed effects for the IID_RW1_Lag123 model using standardized monthly predictors. Table S33. Posterior estimates of random effects for the IID_RW1_Lag123 model using standardized monthly predictors. Table S34. Posterior estimates of temporal effects for the IID_RW1_Lag123 model using standardized monthly predictors. Table S35. District-wise monthly early warning for dengue outbreaks (2025–2026) by using ConvLSTM.

## Abbreviations

The following abbreviations are used in this manuscript:

AI	Artificial Intelligence
DF	Dengue Fever
EWS	Early Warning System
XAI	Explainable Artificial Intelligence
ML	Machine Learning
DL	Deep Learning
CV	Cross-Validation
GLM	Generalized Linear Model
SVM	Support Vector Machine
XGB	Extreme Gradient Boosting
DT	Decision Tree
MLP	Multi-Layer Perceptron
LSTM	Long Short-Term Memory
ConvLSTM	Convolutional Long Short-Term Memory
GWNNR	Geographically Weighted Neural Network Regression
SHAP	Shapley Additive Explanations
IID	Independent and Identically Distributed
RW1	Random Walk of Order One
RW2	Random Walk of Order Two
AR1	Autoregressive Model of Order One
BYM2	Besag–York–Mollié Two Model
VIF	Variance Inflation Factor
LISA	Local Indicators of Spatial Association
DIC	Deviance Information Criterion
WAIC	Watanabe–Akaike Information Criterion
LPML	Log Pseudo-Marginal Likelihood
DENV-1	Dengue virus serotype 1
DENV-2	Dengue virus serotype 2
DENV-3	Dengue virus serotype 3
DENV-4	Dengue virus serotype 4
