# Supplementary material for: District-Level Dengue Early Warning Prediction System in Bangladesh Using Hybrid Explainable AI and Bayesian Deep Learning
Source: Trop Med Infect Dis. 2026 Mar 5;11(3):73. doi: 10.3390/tropicalmed11030073 (PMC13030265; doi:10.3390/tropicalmed11030073)
Supplement: Supplementary file 1 [file tropicalmed-11-00073-s001.zip › tropicalmed-4113353-supplementary.pdf]

Supporting information

1. Spatio-temporal dengue related data selection process

We conducted a comprehensive spatio-temporal feature selection which guided by dengue-related literature and national surveillance records. We collected the district-level datasets for Bangladesh covering the period 2017–2024. We considered two temporal resolutions: i) yearly aggregated district-wise data (2017–2024), and ii) monthly aggregated district-wise data (January 2022–December 2024) (**Table S1**). This dual structure allowed us to capture both long-term epidemiological trends and short-term seasonal fluctuations in dengue transmission in Bangladesh.

Table S1. Description of the variables used in our study

Catagory	Description	Code	Sources
Spatio-temporal	64 districts in Bangladesh	District	
	Districts latitude	Latitude	
	District latitude	Longitude	
	2017-24	Year	
	January 2022 to December 2024	Month	
Cases	Yearly district wise dengue cases 2017-24	Yearly_cases	DGHS
	Monthly district wise dengue cases 2022-24	Monthly_cases	DGHS
Dengue Virus Serotype Distribution (DENV1–DENV4)	Distribution of DENV1 Serotypes in Bangladesh	DENV1	DGHS
	Distribution of DENV2 Serotypes in Bangladesh	DENV2	DGHS
	Distribution of DENV3Serotypes in Bangladesh	DENV3	DGHS
	Distribution of DENV4 Serotypes in Bangladesh	DENV4	DGHS
Climate & Environmental Factors	Yearly average temperature at 2 meters (C)	X11	NASA
	Monthly average temperature at 2 meters (C)	X21	NASA
	Yearly minimum temperature at 2 meters (C)	X12	NASA
	Monthly minimum temperature at 3 meters (C)	X22	NASA
	Yearly maximum temperature at 2 Meters (C)	X13	NASA
	Monthly maximum temperature at 3 Meters (C)	X23	NASA
	Yearly relative humidity at 2 meters (%)	X14	NASA
	Monthly relative humidity at 3 meters (%)	X24	NASA
	Yearly rainfall corrected (mm/day)	X15	NASA
	Monthly rainfall corrected (mm/day)	X25	NASA

	Yearly surface pressure (kPa)	X16	NASA
	Monthly surface pressure (kPa)	X26	NASA
Socio-Demographic & Economic Indicators	Yearly GDP(Billion US$)	X17	World Bank
	Monthly GDP(Billion US$)	X27	World Bank
	Yearly total population (each district)	X18	BBS
	Monthly total population (each district)	X28	BBS
	Yearly poverty head-count ratio(% of population)	X19	BBS
	Monthly poverty head-count ratio(% of population)	X29	BBS
	Yearly adult literacy rate (%)	X110	BBS
	Monthly adult literacy rate (%)	X210	BBS
	Yearly Population growth(%)	X111	BBS
	Monthly Population growth(%)	X211	BBS
	Yearly average household size	X112	DGHS
	Monthly average household size	X212	DGHS
	Yearly access to electricity (% of population)	X113	World Bank
	Monthly access to electricity (% of population)	X213	World Bank
Healthcare System Capacity & Resources	Yearly total number of hospital bed	X114	World Bank
	Monthly total number of hospital bed	X214	World Bank
	Yearly number of of physicians	X115	WHO
	Monthly number of of physicians	X215	WHO
	Yearly domestic general government health expenditure (%)	X116	WHO
	Monthly domestic general government health expenditure (%)	X216	WHO
	Yearly density of nursing and midwifery personnel (per 10 000 population)	X117	WHO
	Monthly density of nursing and midwifery personnel (per 10 001 population)	X217	WHO
	Yearly UHC Service Coverage Index (SDG 3.8.1)	X118	WHO
	Monthly UHC Service Coverage Index (SDG 3.8.2)	X218	WHO
Land Use and Land Cover Indicators	Yearly land use	X119	World Bank
	Monthly land use	X219	World Bank
	Yearly arble land	X120	World Bank
	Monthly arble land	X220	World Bank
	Yearly agriculture land	X121	World Bank
	Monthly agriculture land	X221	World Bank

We used District boundaries (n = 64) as the spatial unit of analysis, with latitude and longitude coordinates assigned to each district centroid. Dengue case data were obtained from the Directorate General of Health Services (DGHS)[1],

including both yearly and monthly counts. We also incorporated Virus serotype distributions (DENV1–DENV4) to account for genetic diversity and its potential influence on outbreak dynamics[2].

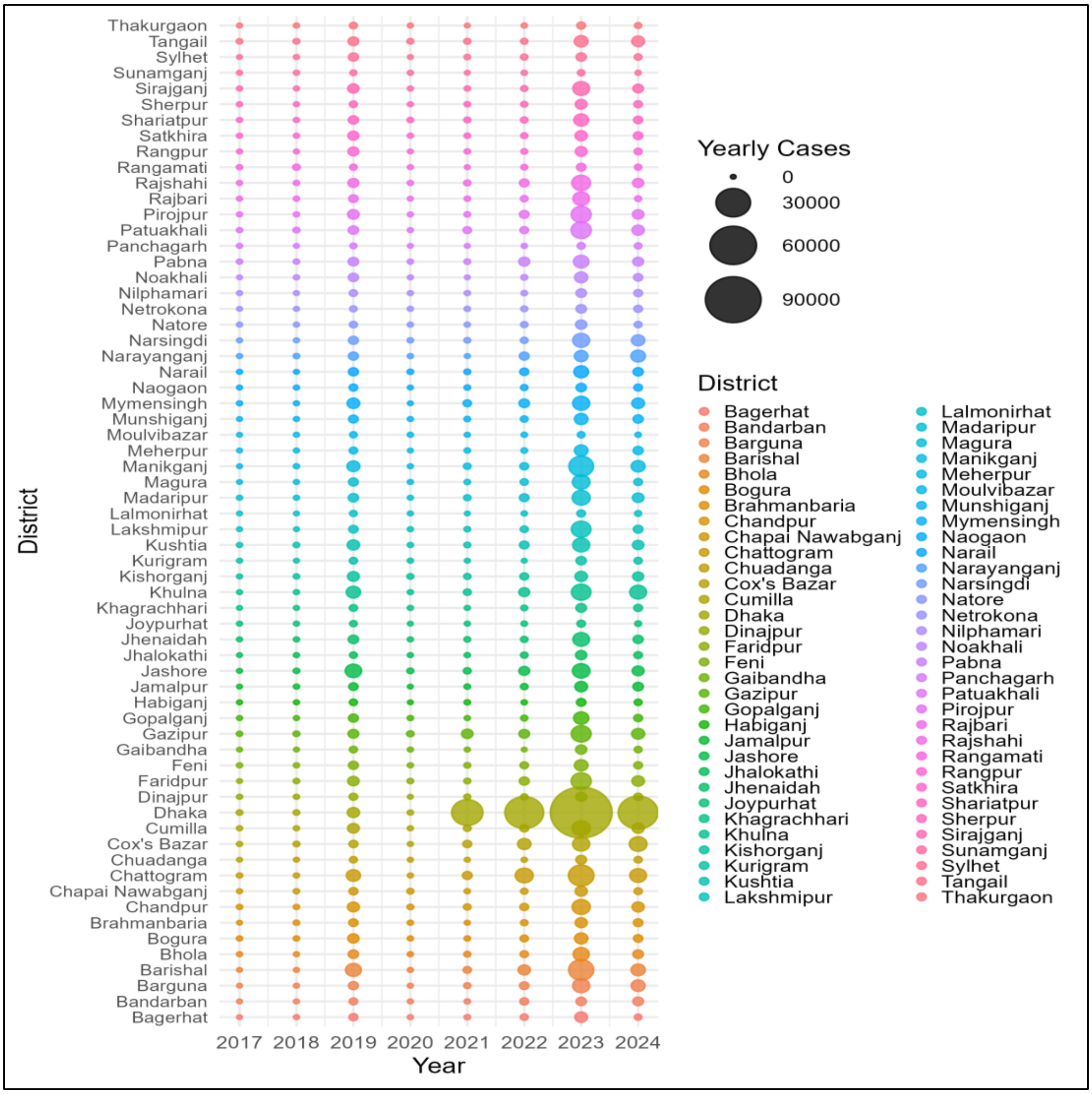

Figure S1. All Bangladesh's districts with dengue cases during the study period (2017-24). 64 dengue affected districts are shown. Year to year, the number of dengue cases per districts varies significantly. The bubble size represents the number of dengue cases per year per districts. The bigger the bubble size, the greater the number of cases in a districts per year.

We extracted the climate and the environmental features (average, minimum, and maximum temperature, relative humidity, rainfall, and surface pressure) from NASA[3] datasets at district resolution. These variables were selected based on their established role in vector ecology and dengue transmission in Bangladesh. The socio-demographic and the economic indicators (population, GDP, poverty ratio, literacy rate, household size, electricity access) were collected from Bangladesh Bureau of Statistics (BBS)[4], DGHS[2], and World Bank[5].The Healthcare system capacity indicators (hospital beds, physicians, health expenditure, nursing density, UHC coverage index) were obtained from WHO[6] and World Bank[5] databases. The land use and land cover indicators (arable and agricultural land) were included to capture environmental drivers of vector breeding habitats[7].

Certain features mentioned in dengue literature were excluded due to data unavailability or redundancy. For example, drought indices such as the Palmer Drought Severity Index (PDSI) were not included, as precipitation-related features were hypothesized to capture similar information. Likewise, global climatic oscillation indices (ENSO, NAO) were excluded due to the lack of district-level resolutions. Some regional factors such as wetlands and fine-scale land cover were also omitted due to incomplete or inconsistent data availability.

All datasets were harmonized to the district boundaries and aligned temporally with the dengue case data. Quality control procedures included cross-checking case counts with DGHS reports, ensuring consistency across years, and excluding records with unknown etiology. **Figure S1 and S2** illustrate the yearly and monthly distribution of dengue cases across districts, highlighting spatial heterogeneity and temporal variation.

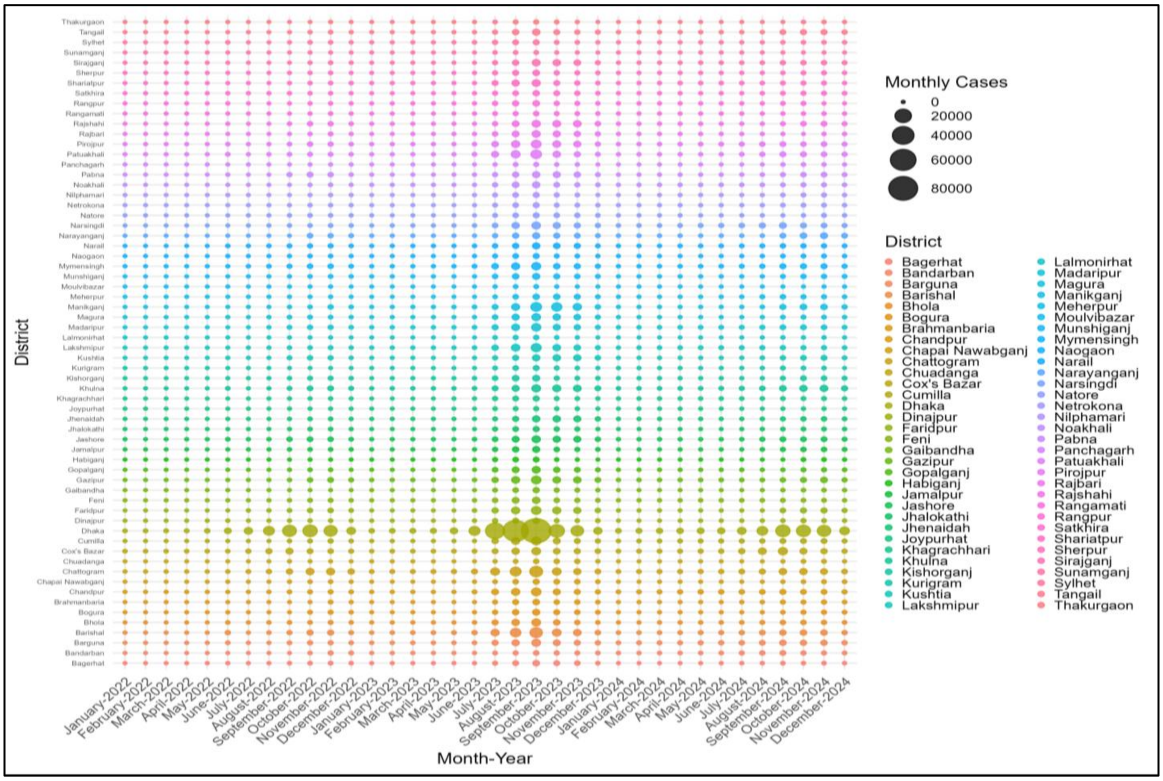

Figure S2. All Bangladesh's districts with dengue cases during the study period (2022-24). 64 dengue affected districts are shown. month to month, the number of dengue cases per districts varies significantly.

2. Missing data imputation strategy

We systematically imputed all the missing values to ensure continuity and reliability of the district-level datasets using advanced statistical and machine learning techniques based on the type of variable. Simple mean imputation was avoided, as it can distort variance and underestimate uncertainty. Instead of this, we adopted some imputation methods that preserve variability and account for both spatial and temporal dependencies inherent in dengue and climate data.

For the climatic and the environmental variables (temperature, rainfall, humidity, surface pressure), we imputed the missing values using *spatio-temporal regression* and *kriging* interpolation[8]. These approaches leveraged geographic proximity between districts and temporal continuity across months and years, ensuring that imputed values reflected both local climatic conditions and seasonal trends.

For the socio-demographic and the healthcare indicators(population, poverty ratio, literacy rate, hospital beds, physicians), we applied multiple imputation by chained equations(MICE)[9] and MissForest (random forest imputation)[10] methods. These methods allowed us to incorporate auxiliary predictors, capture non-linear relationships and generate multiple plausible imputations, thereby reducing bias and improving the robustness.

For the dengue case data(both yearly and monthly), missing records were rare but critical. In these instances, we employed Bayesian hierarchical imputation[11], borrowing strength from neighboring districts and adjacent time periods. This approach respected spatial clustering and temporal continuity of dengue, while explicitly modeling uncertainty in the imputed values.

All the imputation procedures were conducted prior to statistical and machine learning analyses, and diagnostic checks were performed to confirm that the distributions of imputed values were consistent with observed data. By combining spatio-temporal, regression-based, and machine learning imputation techniques, we ensured that the final datasets maintained integrity across all the districts, months and years, providing a reliable foundation for subsequent dengue modeling and early warning system development.

3. Spatio-temporal weighted correlation analysis

We performed a spatio-temporal weighted correlation analysis on district-level dengue data for both yearly and monthly panels to quantify the joint spatial and temporal dependence between dengue cases and the related predictors. Spatial dependence was captured using a *Queen contiguity* based spatial weights matrix W_S, and we modeled the temporal dependence using a first-order temporal adjacency matrix W_T. These were combined into a spatio-temporal weight matrix via the *Kronecker product*:

$$W_{ST} = W_T \otimes W_S$$

Where, $\otimes$ denotes the *Kronecker product*. Dengue cases Y and standardized predictor variables X_k were then spatially and temporally lagged as:

$Y^{ST} = W_{ST} Y$, $X_k^{ST} = W_{ST} X_k$

The spatio-temporal weighted correlation for each predictor was computed using the Pearson correlation:

$$r_k = cor(Y^{ST}, X_k^{ST})$$

where r_k represents the correlation between the lagged dengue cases and lagged predictor k, providing a measure of association that incorporates both spatial clustering and temporal continuity.

4. Rationale for machine learning and deep learning model selection and threshold choice

We applied a combination of statistical, machine learning (ML), and deep learning (DL) models to evaluate dengue outbreaks across yearly and monthly datasets. In our research, The Generalized Linear Model (GLM) served as a transparent statistical baseline model that widely used in public health researches[12]. The Support Vector Machine (SVM) was included for its ability to handle high-dimensional data and capture non-linear relationships common in disease transmission[13]. eXtreme Gradient Boosting (XGB) was selected as a state-of-the-art ensemble method that reduces overfitting and models complex predictor interactions[14]. In addition, the Decision Tree (DT) model was incorporated to provide an interpretable, rule-based framework for identifying hierarchical risk structures.

The DL model were chosen to address the spatio-temporal nature of dengue in Bangladesh. The Multi-Layer Perceptron (MLP) captured non-linear associations across socio-demographic, climatic, healthcare, and land-use features[15]. Long Short-Term Memory (LSTM) networks modeled sequential dependencies in outbreaks, implemented with a two-time sequence design to reflect epidemiological continuity[16]. Convolutional LSTM (ConvLSTM) extended this capacity by integrating spatial features, using a two-dimensional grid of district coordinates to capture both spatial clustering and temporal progression[17]. Furthermore, the Geographically Weighted Neural Network Regression (GWNNR) model was applied to explicitly account for spatial non-stationarity

by allowing predictor–dengue relationships to vary across districts. Together, these models allowed comparison of traditional statistical approaches with modern machine learning and deep learning architectures.

Table S2. Sensitivity analysis of dengue outbreak prediction using different cutoffs and probability thresholds

Cutoff type	Cutoff value	Probability threshold	AUC (mean ± SD)	Accuracy (mean)	F1 score (mean)
P25	4	0.3	–	1	1
P25	4	0.5	–	1	1
Median (P50)	57	0.3	0.961 ± 0.078	0.899	0.795
Median (P50)	57	0.5	0.961 ± 0.078	0.899	0.795
P75	619	0.3	0.900 ± 0.200	0.955	0.8
P75	619	0.5	0.900 ± 0.200	0.955	0.8
P90	2049.8	0.3	0.900 ± 0.200	0.984	0.393
P90	2049.8	0.5	0.900 ± 0.200	0.984	0.393
Mean	1102.05	0.3	0.900 ± 0.200	0.981	0.782
Mean	1102.05	0.5	0.900 ± 0.200	0.981	0.782
Baseline + SD	1640.38	0.3	0.898 ± 0.199	0.988	0.796
Baseline + SD	1640.38	0.5	0.898 ± 0.199	0.988	0.796
Baseline − SD	0	0.3	–	1	1
Baseline −SD	0	0.5	–	1	1

5. Cross-validation and parametric tuning

We applied five-fold cross-validation across all machine learning and deep learning models to ensure robust model performance and minimize overfitting. Hyperparameters were systematically tuned to balance predictive accuracy with generalizability. For the XGBoost model, tuning involved varying the number of estimators (up to 200), maximum tree depth (up to 4), and learning rate (down to 0.01). Subsampling and column sampling were both set to 0.6 to introduce randomness and reduce variance, and the regularization was controlled through *min_child_weight* = 1 and *gamma* = 0. The SVM model were optimized by using radial basis function kernels, with penalty parameter *C* and kernel coefficient gamma adjusted to achieve an optimal bias–variance trade-off. The GLM with Elastic Net regularization were tuned by varying the *L1/L2* mixing ratio and adjusting the regularization strength (*C*) to achieve sparsity and stability.

The DL models (MLP, LSTM and ConvLSTM) were tuned for hidden layer sizes ranging from 32 to 64 units, *dropout rates* between 0.2 and 0.3 to prevent overfitting, and *batch sizes* of 16 to balance computational efficiency with convergence stability[18]. Learning rates were varied between 0.001 and 0.01, and training epochs ranged from 50 to 500 depending on convergence behavior. LSTM models were trained on sequential inputs with a *two-timestep* design to reflect the epidemiological continuity of dengue transmission across consecutive periods[19]. ConvLSTM models were implemented in a two-dimensional structure, reshaping district-level features into spatial grids with latitude and longitude coordinates represented as “columns” with one row and channel. This design allowed the model to simultaneously capture spatial clustering and temporal progression of dengue outbreaks.

6. Distributional assessment and likelihood selection

Prior to Bayesian modeling, we examined the empirical distribution of dengue counts to select an appropriate likelihood. For yearly dataset, pronounced overdispersion (variance (34,536,049.48) > mean (1102.05)). Skewness was 15, and 44 zero counts (8.59%) were observed in this dataset. Monthly dataset exhibited similar overdispersion, with a mean of 311.71, variance 5,916,357.51, skewness 25.82, and 558 zero counts (24.22%). Given the overdispersion, right skew, and presence of zeros, we selected the *Negative Binomial (NB) likelihood*, which introduces a dispersion parameter to allow variance exceeding the mean[20–25]:

$$Y_{it} \sim NB\,(\mu_{it}, \phi)\,, \quad var(Y_{it}) = \mu_{it} + \frac{\mu_{it}^2}{\phi}$$

Where, Y_{it} denotes cases in district i at time t, μ_{it} is the expected incidence, and ϕ is the dispersion parameter.

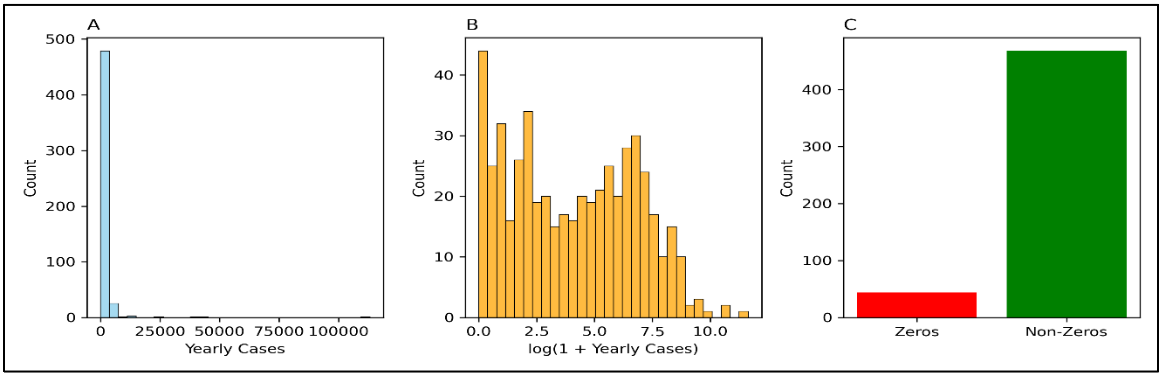

Figurer S3. Distribution of yearly dengue cases in Bangladesh**.** Panel (A) shows the histogram of raw yearly cases, (B) shows the log-transformed distribution, and (C) compares zero versus non-zero cases.

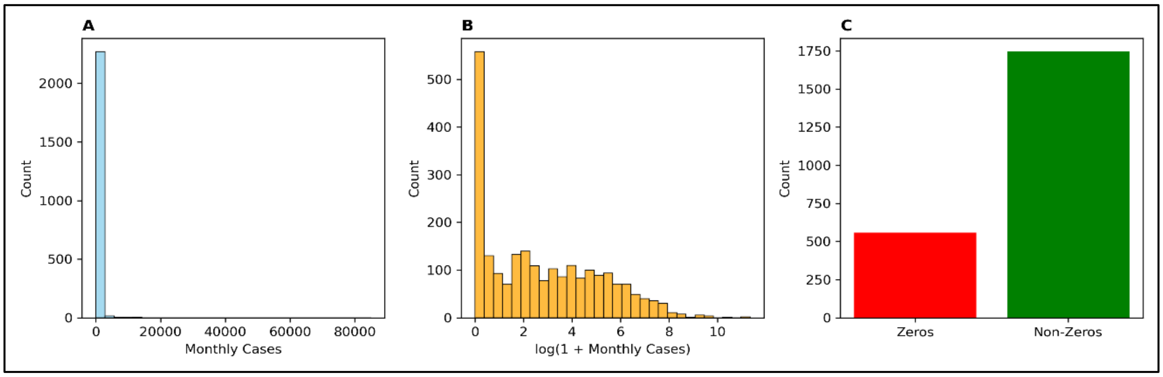

Figurer S4. Distribution of monthly dengue cases in Bangladesh**.** Panel (A) shows the histogram of raw monthly cases, (B) shows the log-transformed distribution, and (C) compares zero versus non-zero cases.

7. Bayesian spatio-temporal modeling of dengue incidence

To investigate spatio-temporal patterns of dengue incidence across Bangladeshi districts, we employed a Bayesian hierarchical framework using the Integrated Nested Laplace Approximation (INLA). The models accounted for both spatial and temporal dependencies, included covariate selection via machine learning, and incorporated lagged effects to capture delayed dengue transmission[20–25].

7.1. Model Formulation

Let Y_{it} denote the monthly or yearly dengue cases in district i at time t. We assumed a negative binomial likelihood based on the distribution' assumption.

$$Y_{it} \sim NB\ (\mu_{it}, \phi)\ ,$$

with the log-linear predictor:

$$\log(\mu_{it}) = \beta_0 + \sum_{k=1}^{p} \beta_k X_{kit} + u_i + v_t + \gamma_t$$

Here, X_{kit} represents covariates, β_k are fixed-effect coefficients, u_i denotes spatial effects, and v_t denotes temporal effects. Spatial effects were modeled as either unstructured IID ($u_i \sim N(0, \sigma_u^2)$) or using a BYM2 structure:

$$u_i = \sqrt{\phi} u_i^{\text{structured}} + \sqrt{1-\phi} u_i^{\text{structured}},$$

with $u_i^{\text{structured}}$ following a conditional autoregressive (CAR) prior based on district adjacency. Temporal effects were modeled as IID ($v_t \sim N(0, \sigma_v^2)$), first- or second-order random walks (RW1, RW2), or autoregressive processes (AR1):

$$\gamma_t \sim \begin{cases} RW1(\sigma_\gamma^2) & (first - order\ random\ walk) \\ RW2(\sigma_\gamma^2) & (second - order\ random\ walk) \\ AR1(\rho) & (\text{autoregressive}) \end{cases}$$

$$v_t = \rho v_{t-1} + \varepsilon_t\ ,$$

$$\varepsilon_t \sim N(0, \sigma_v^2)),$$

Eight combinations of spatial and temporal structures were considered, summarized in **Table S2**.

Table S3. The description of Baysian spatio temporal model's used in our study.

Model	Spatial effect	Temporal effect
IID_ IID	IID	IID
IID_ RW1	IID	RW1
IID_ AR1	IID	AR1
IID_ RW2	IID	RW2
BYM2_ IID	BYM2	IID
BYM2_ RW1	BYM2	RW1
BYM2_ AR1	BYM2	AR1
BYM2_ RW2	BYM2	RW2

IID: Independent and Identically Distributed; RW1: Random Walk (order 1); AR1: Autoregressive (order 1); RW2: Random Walk (order 2); BYM2: Besag–York–Mollié 2 model.

Each model was first fitted using all covariates, and subsequently with the top 10 features selected via SHAP analysis from machine learning models

7.2. Lagged models

To account for delayed effects of dengue transmission, we incorporated lagged responses:

$$\log(\mu_{it}) = \beta_0 + \sum_{k=1}^{p} \beta_k X_{kit} + \sum_{l=1}^{L} \delta_l Y_{i,t-l} + u_i + v_i + \gamma_t$$

where $Y_{i,t-l}$ is the dengue count lagged by l months or year. We tested models with lags of 1, 2, and 3 months and years (Lag1, Lag12, Lag123) and selected the lag that optimized model performance[26].

7.3. Model evaluation

Model performance was assessed using three standard Bayesian metrics. The Deviance Information Criterion (DIC) is defined as

$$DIC = \bar{D} + p_D,\ \bar{D} = E[D(\theta|Y)],\ \ p_D = \bar{D} - \hat{\theta}\ ,$$

where $D(\theta|Y)$ denotes the deviance, $\bar{D}$ is its posterior mean, and p_D represents the effective number of parameters. The Watanabe–Akaike Information Criterion (WAIC) is

$$WAIC = -2\sum_{i=1}^{n} \log\left(\frac{1}{S}\sum_{s=1}^{S} p\left(Y_i\middle|\theta^{(s)}\right)\right) + 2p_{waic}\ ,$$

where S is the number of posterior samples and p_{waic} is the effective number of parameters.
The Log Pseudo-Marginal Likelihood (LPML) is

$$LPML = \sum_{i=1}^{n} \log\left(CPO_i\right)\ , CPO_i = (Y_i|Y_{-i})$$

with CPO_i denoting the leave-one-out predictive density for observation i. The best-fitting model was selected based on minimal DIC and WAIC, and maximal LPML.

7.4.Posterior analysis

Posterior estimates of fixed effects, spatial random effects, and temporal trends were obtained from the selected model. Relative risks were computed as

$$RR_k = \exp\left(\beta_k\right),$$

where β_k is the posterior coefficient for covariate k. For disease counts, the likelihood was modeled using a Negative Binomial distribution:

$$Y_{it} = NB(\mu_{it}, \phi), \log(\mu_{it}) = X_{it}\beta + u_i + v_t,$$

where μ_{it} is the expected count for region i at time t, ϕ is the dispersion parameter, X_{it} are covariates, β are fixed effects, u_i represents spatial random effects, and v_t captures temporal trends. Posterior predictive distributions were used for model validation and forecasting, enabling inference on disease risk patterns across space and time.

Table S4. Sumarry statistics of yearly dengue cases over 64 district in Bangladesh from 2017 to 2024

Year	Min	Q1	Median	Q3	Max	Mean± SD
2017	0	0	1.5	4	29	3.48 ± 5.35
2018	0	2	4	10.5	105	9.94 ± 16.06
2019	47	213.5	611	934.25	4068	774.12 ± 789.49
2020	0	2	4	8.25	97	10.06 ± 17.19
2021	0	9.5	25	72.75	23625	444.2 ± 2948.15
2022	0	58.5	129	416	39410	974.72 ± 4935.25
2023	102	901.75	2190.5	4737.75	113233	5018.42 ± 14101.53
2024	5	231.25	525.5	1362	42045	1581.47 ± 5250.81

Min: Minimum; Q1: First quartile; Q3: 3rd quartile; Max: Maximum; SD: Standard deviation

Table S5. Yearly total aggregated dengue cases and total affected districts of Bangladesh

A. Year	Total cases	Affected districts
2017	223	43
2018	636	58

2019	49544	64
2020	644	55
2021	28429	58
2022	62382	62
2023	321179	64
2024	101214	64

Table S6. Monthly dengue case summary from January 2022 to December 2024

Year	Month	Min	Q1	Median	Q3	Max	Mean± SD	Percentage
2022	January	0	0	0	0	59	2 ± 9	1.59
2022	February	0	0	0	0	69	2 ± 10	1.37
2022	March	0	0	0	1	80	3 ± 11	1.81
2022	April	0	0	0	1	99	3 ± 13	1.59
2022	May	0	0	0	1	260	6 ± 33	1.7
2022	June	0	0	1	8	961	27 ± 123	1.55
2022	July	0	0	0	3	2244	42 ± 281	1.58
2022	August	0	0	1	6	5901	109 ± 740	1.56
2022	September	0	2	12	49	12284	251 ± 1538	1.56
2022	October	0	11	42	154	13828	343 ± 1731	1.56
2022	November	0	27	54	189	10581	302 ± 1332	1.56
2022	December	0	4	11	38	2717	78 ± 349	1.55
2023	January	0	0	1	5	279	9 ± 36	1.59
2023	February	0	0	2	6	355	11 ± 46	1.5
2023	March	0	0	2	7	429	13 ± 55	1.54
2023	April	0	0	2	7	521	15 ± 67	1.52
2023	May	0	0	2	8	1385	32 ± 174	1.58
2023	June	0	2	8	32	6114	125 ± 764	1.57
2023	July	17	110	198	456	29570	810 ± 3681	1.56
2023	August	65	354	672	1358	59142	1935 ± 7344	1.56
2023	September	89	587	1318	2727	85003	3178 ± 10563	1.56
2023	October	9	210	480	1235	16630	1059 ± 2153	1.56
2023	November	4	114	282	682	9323	636 ± 1220	1.56
2023	December	0	31	80	178	2277	145 ± 290	1.56
2024	January	0	2	5	13	368	17 ± 47	1.61
2024	February	0	2	6	16	491	22 ± 63	1.58
2024	March	0	2	8	20	612	27 ± 79	1.58
2024	April	0	3	8	28	790	35 ± 103	1.58

2024	May	0	3	8	34	1015	45 ± 132	1.58
2024	June	0	3	9	44	1345	57 ± 174	1.56
2024	July	0	5	14	61	2615	102 ± 340	1.56
2024	August	0	8	26	118	5578	201 ± 731	1.57
2024	September	0	41	107	402	13695	483 ± 1740	1.56
2024	October	0	86	173	412	12894	482 ± 1607	1.56
2024	November	2	88	154	402	11536	463 ± 1440	1.56
2024	December	0	24	58	129	3920	152 ± 490	1.56

Min: Minimum; Q1: First quartile; Q3: 3rd quartile; Max: Maximum; SD: Standard deviation

Table S7. Sumarry of monthly dengue cases of Bangladesh from 2022 to 2024

Month	Min	Q1	Median	Q3	Max	Mean± SD	Percentage	Highest affected year	Lowest affected year
January	126	346	566	812	1059	584 ± 467	33.35	2024	2022
February	146	439	732	1063	1394	757 ± 624	33.32	2024	2022
March	166	504	843	1276	1710	906 ± 774	33.32	2024	2022
April	189	588	986	1598	2209	1128 ± 1017	33.33	2024	2022
May	352	1187	2022	2438	2853	1742 ± 1274	33.33	2024	2022
June	1739	2700	3661	5820	7978	4459 ± 3195	33.33	2023	2022
July	2660	4590	6520	29180	51841	20340 ± 27349	33.33	2023	2022
August	6991	9916	12841	68329	123817	47883 ± 65826	33.33	2023	2022
September	16092	23515	30938	117174	203410	83480 ± 104127	33.33	2023	2022
October	21932	26406	30879	49342	67805	40205 ± 24317	33.33	2023	2022
November	19334	24493	29652	35164	40676	29887 ± 10673	33.33	2023	2022
December	5024	7156	9288	9516	9745	8019 ± 2604	33.33	2024	2022

Min: Minimum; Q1: First quartile; Q3: 3rd quartile; Max: Maximum; SD: Standard deviation

Table S8. District wise summary of yearly dengue cases with highest year and lowest affected year

District	Min	Q1	Median	Q3	Max	Mean± SD	Highest affected year	Lowest affected year
Bagerhat	0	2.75	70.5	224	1720	299.75 ± 586.79	2023	2017
Bandarban	0	2.5	138.5	436	841	274.5 ± 344.9	2024	2017
Barguna	0	2.75	281.5	1072.75	4592	1026.62 ± 1656.31	2023	2017
Barishal	0	3.5	861.5	2862	13603	2698 ± 4616.92	2023	2017
Bhola	2	7.75	154.5	764.5	3861	717.38 ± 1312.79	2023	2018
Bogura	8	13.75	130	600.5	2057	483.75 ± 734.53	2023	2020
Brahmanbaria	0	8.25	59.5	470.75	1433	316.5 ± 497.36	2023	2017
Chandpur	13	31.5	233	1413.75	5536	1118 ± 1886.89	2023	2017
Chapai Nawabganj	2	11.75	80.5	271.75	1448	281.12 ± 489.57	2023	2017
Chattogram	3	13	1545.5	4567	14200	3368.75 ± 4847.25	2023	2020
Chuadanga	0	1	5	171	877	156.12 ± 302.29	2023	2017

Cox's Bazar	2	32.5	372.5	2716.5	5156	1584.88 ± 2163.2	2024	2020
Cumilla	5	6.5	252	1305.75	5387	1075.62 ± 1834.89	2023	2017
Dhaka	11	29.75	12565.5	40068.75	113233	27486.12 ± 39022.26	2023	2020
Dinajpur	0	7	64	307.75	910	211.88 ± 313.22	2023	2020
Faridpur	0	23	342	1266.25	7502	1376.38 ± 2549.99	2023	2017
Feni	7	7.75	153	389.25	2228	435.12 ± 755.06	2023	2017
Gaibandha	0	0	2.5	168.25	1052	176.75 ± 362.8	2023	2017
Gazipur	13	82.25	842.5	1309.25	7252	1509 ± 2406.49	2023	2017
Gopalganj	2	3.5	42	403.25	3156	522.62 ± 1085.48	2023	2017
Habiganj	0	1.75	13	171.75	493	109.62 ± 173.44	2023	2020
Jamalpur	0	6	72.5	528.25	1692	383.5 ± 598.22	2023	2017
Jashore	1	42	534.5	2020.5	4974	1440.25 ± 1977.28	2023	2017
Jhalokathi	0	3.5	62	169	1053	198.38 ± 358.06	2023	2017
Jhenaidah	1	6.25	68	691.75	4194	721.38 ± 1437.43	2023	2017
Joypurhat	0	1.75	10	64.25	264	56 ± 92.57	2023	2020
Khagrachhari	0	3.25	28.5	242.75	827	178.75 ± 288.71	2023	2017
Khulna	1	4	541.5	3086	6853	1874.38 ± 2567.34	2023	2017
Kishorganj	1	39.5	199	1041.25	1549	533.12 ± 636.7	2023	2017
Kurigram	0	0.75	3.5	208.75	708	142.38 ± 247.46	2023	2018
Kushtia	5	20	278	1215.5	4556	977.12 ± 1560.79	2023	2017
Lakshmipur	0	3.75	108	476	6950	1020.88 ± 2405.4	2023	2017
Lalmonirhat	0	0	4.5	80.75	305	62.62 ± 107.24	2023	2017
Madaripur	3	4	306	842	5386	991.12 ± 1827.62	2023	2020
Magura	0	4.25	111.5	371.5	5031	767.62 ± 1734.62	2023	2017
Manikganj	0	8.25	232	1979.25	12952	2208.75 ± 4440.2	2023	2017
Meherpur	0	1.5	24	356.5	2176	392.5 ± 756.14	2023	2017
Moulvibazar	0	3.5	9.5	38.25	129	35.12 ± 53.65	2023	2017
Munshiganj	0	3.5	86	566.25	2167	443.25 ± 744.04	2023	2017
Mymensingh	0	5.75	514.5	1679	4533	1119.12 ± 1554.48	2023	2020
Naogaon	1	2.5	64.5	286.5	740	181.75 ± 257.12	2023	2017
Narail	0	19.25	216.5	637.75	2841	578.25 ± 958.85	2023	2018
Narayanganj	1	8.5	308	1015.5	2729	773 ± 1086.89	2024	2021
Narsingdi	2	15	92	982	4523	928.38 ± 1616.45	2023	2017
Natore	2	5.75	55.5	161.25	1063	189.75 ± 360.22	2023	2017
Netrokona	0	3.25	38.5	153.75	762	156.62 ± 265.28	2023	2017
Nilphamari	1	4.25	16	236.25	770	167.12 ± 271.21	2023	2018
Noakhali	0	2.25	25.5	493.5	1985	395.38 ± 692.91	2023	2017
Pabna	9	15.75	402.5	924	3388	763.12 ± 1144.38	2023	2020
Panchagarh	0	0	4	68.25	187	45.75 ± 70.98	2023	2018
Patuakhali	4	35	275.5	916.75	7579	1302.12 ± 2586.67	2023	2020

Pirojpur	2	7.5	277	1162.75	7361	1285.5 ± 2507.63	2023	2017
Rajbari	1	8	35.5	220.5	4176	608.75 ± 1450.16	2023	2020
Rajshahi	3	7.5	300	941.75	6078	1077.25 ± 2062.73	2023	2017
Rangamati	0	7.25	69	102	491	105.75 ± 162.14	2023	2021
Rangpur	0	1	29	377.25	1299	309.25 ± 507.91	2023	2020
Satkhira	4	5.5	47	611	1500	383.75 ± 568.36	2023	2017
Shariatpur	3	17.25	89	479.75	2901	525.75 ± 990.04	2023	2017
Sherpur	1	2.75	40.5	194.5	1281	226.75 ± 437.48	2023	2018
Sirajganj	1	2	42.5	902	4371	799.12 ± 1507.17	2023	2018
Sunamganj	0	3.25	6	17	102	21.62 ± 35.88	2023	2021
Sylhet	0	1.5	61	281.5	711	206.38 ± 301.37	2023	2017
Tangail	20	47.25	76	1092	2319	652.25 ± 917.36	2023	2017
Thakurgaon	0	1.75	7.5	104.75	309	73.25 ± 114.1	2023	2021

Min: Minimum; Q1: First quartile; Q3: 3rd quartile; Max: Maximum; SD: Standard deviation

Table S9. District wise summary of monthly dengue cases from 2022 to 2024 in Bangladesh.

District	Min	Q1	Median	Q3	Max	Mean ± SD	Highest Cases	Highest affected month	Highest affected year	Lowest Cases	Lowest affected month	Lowest affected year
Bagerhat	0	1	5	36	909	74 ± 184	909	September	2023	0	January	2022
Bandarban	0	6	26	140	439	79 ± 107	439	September	2024	0	January	2022
Barguna	1	22	90	314	2724	308 ± 529	2724	September	2023	1	January	2022
Barishal	3	43	132	613	8874	751 ± 1726	8874	September	2023	3	January	2022
Bhola	0	6	32	105	2758	205 ± 525	2758	September	2023	0	January	2022
Bogura	0	0	2	74	1217	103 ± 243	1217	September	2023	0	January	2022
Brahmanbaria	0	0	3	66	864	76 ± 171	864	September	2023	0	January	2022
Chandpur	3	8	98	302	3722	341 ± 726	3722	September	2023	3	January	2022
Chapai Nawabganj	0	1	2	46	634	61 ± 140	634	September	2023	0	January	2022
Chattogram	6	94	200	1086	9792	995 ± 1910	9792	September	2023	6	January	2022
Chuadanga	0	0	0	11	302	34 ± 80	302	October	2023	0	January	2022
Cox's Bazar	27	53	183	638	3150	516 ± 754	3150	September	2024	27	January	2023
Cumilla	1	3	51	206	3648	302 ± 705	3648	September	2023	1	January	2022
Dhaka	59	476	2260	10820	85003	8749 ± 17153	85003	September	2023	59	January	2022
Dinajpur	0	1	5	38	658	58 ± 135	658	September	2023	0	January	2024
Faridpur	0	0	4	275	3859	354 ± 807	3859	September	2023	0	January	2022
Feni	2	4	22	92	1617	124 ± 315	1617	September	2023	2	January	2022

Gaibandha	0	0	0	27	701	47 ± 133	701	September	2023	0	January	2022
Gazipur	1	17	33	292	3722	364 ± 760	3722	September	2023	1	January	2022
Gopalganj	0	0	4	40	1958	139 ± 378	1958	September	2023	0	January	2022
Habiganj	0	0	0	3	448	33 ± 96	448	September	2023	0	January	2022
Jamalpur	0	1	2	60	1196	100 ± 234	1196	September	2023	0	January	2022
Jashore	0	4	22	287	2273	252 ± 480	2273	September	2023	0	February	2022
Jhalokathi	0	0	0	50	601	55 ± 124	601	September	2023	0	January	2022
Jhenaidah	0	2	8	78	1442	159 ± 374	1442	October	2023	0	January	2022
Joypurhat	0	0	1	6	168	14 ± 35	168	September	2023	0	January	2022
Khagrachhari	0	0	11	42	610	55 ± 121	610	September	2023	0	January	2022
Khulna	0	15	52	400	2735	406 ± 679	2735	September	2023	0	January	2022
Kishorganj	0	4	10	118	1182	116 ± 254	1182	September	2023	0	January	2022
Kurigram	0	0	0	8	505	39 ± 103	505	September	2023	0	January	2022
Kushtia	0	1	7	189	1538	195 ± 403	1538	October	2023	0	January	2022
Lakshmipur	1	12	40	124	4446	317 ± 856	4446	September	2023	1	January	2022
Lalmonirhat	0	0	0	4	244	17 ± 50	244	September	2023	0	January	2022
Madaripur	0	11	48	192	3444	281 ± 653	3444	September	2023	0	January	2022
Magura	0	3	6	78	2645	197 ± 532	2645	September	2023	0	January	2022
Manikganj	0	5	11	232	5591	508 ± 1264	5591	September	2023	0	January	2022
Meherpur	0	1	8	66	771	95 ± 200	771	October	2023	0	January	2022
Moulvibazar	0	0	1	2	106	8 ± 22	106	September	2023	0	January	2022
Munshiganj	0	6	12	98	1265	115 ± 255	1265	September	2023	0	January	2022
Mymensingh	1	10	54	282	3067	293 ± 607	3067	September	2023	1	January	2022
Naogaon	0	2	6	41	517	47 ± 104	517	September	2023	0	January	2022
Narail	0	12	32	139	1388	146 ± 283	1388	September	2023	0	January	2022
Narayanganj	0	5	21	229	1178	182 ± 315	1178	September	2023	0	January	2022
Narsingdi	0	7	37	303	2569	274 ± 525	2569	September	2023	0	January	2022
Natore	0	0	2	26	500	48 ± 109	500	September	2023	0	January	2022
Netrokona	0	0	5	32	538	46 ± 105	538	September	2023	0	January	2022
Nilphamari	0	0	4	15	540	45 ± 110	540	September	2023	0	January	2022
Noakhali	0	2	13	50	1370	105 ± 272	1370	September	2023	0	January	2022
Pabna	0	1	8	185	1376	172 ± 330	1376	September	2023	0	January	2022
Panchagarh	0	0	0	3	135	12 ± 28	135	September	2023	0	January	2022
Patuakhali	2	5	21	193	5410	389 ± 1030	5410	September	2023	2	January	2022
Pirojpur	1	7	32	244	4099	357 ± 821	4099	September	2023	1	January	2022

Rajbari	0	0	6	20	2131	159 ± 436	2131	September	2023	0	January	2022
Rajshahi	0	4	12	192	2268	248 ± 553	2268	September	2023	0	January	2022
Rangamati	0	0	4	17	290	25 ± 57	290	September	2023	0	January	2022
Rangpur	0	1	8	14	911	69 ± 186	911	September	2023	0	January	2022
Satkhira	0	2	8	52	751	75 ± 156	751	September	2023	0	January	2022
Shariatpur	0	4	18	45	2138	156 ± 423	2138	September	2023	0	January	2022
Sherpur	0	0	5	32	878	66 ± 171	878	September	2023	0	January	2022
Sirajganj	0	0	9	124	1876	175 ± 418	1876	September	2023	0	January	2022
Sunamganj	0	0	0	2	89	6 ± 18	89	September	2023	0	January	2022
Sylhet	0	3	6	40	545	49 ± 116	545	September	2023	0	January	2022
Tangail	0	2	18	140	1482	146 ± 302	1482	September	2023	0	January	2022
Thakurgaon	0	0	0	4	209	17 ± 44	209	September	2023	0	January	2022

Min: Minimum; Q1: First quartile; Q3: 3rd quartile; Max: Maximum; SD: Standard deviation

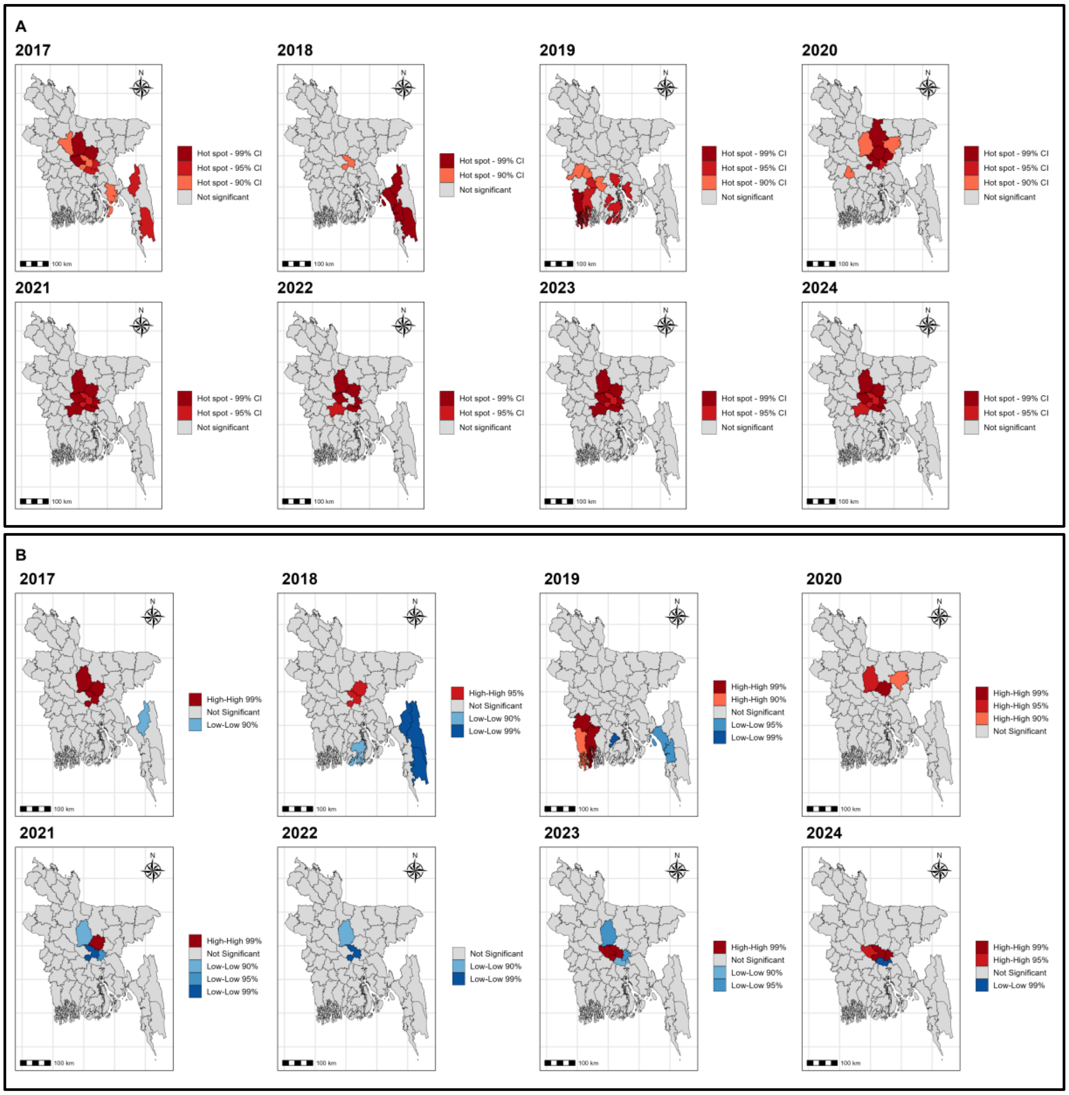

Figure S5. A) Hotspot and cold spot analysis of dengue cases using Getis-Ord Gi*, highlighting statistically significant clusters of elevated and reduced transmission risk across districts from 2017 to 2024 and B) Spatial autocorrelation and clustering analysis based on Local Moran's I (LISA), showing the strength and direction of spatial dependence with confidence intervals at 90%, 95%, and 99%, thereby identifying areas of persistent transmission and emerging outbreak zones. Maps were generated in RStudio (version 4.5.1) using the sf library and the shapefile bgd_adm_bbs_20201113_SHP (administrative level 2), obtained from the Bangladesh Bureau of Statistics (BBS).

Table S10. Cold spot and hot spot analysis of yearly dengue cases of Bangladesh from 2017 to 2024.

Year	2017		2018		2019		2020		2021		2022		2023		2024	
**District**	**Gi Z**	**HotCold**	**Gi Z**	**HotCold**	**Gi Z**	**HotCold**	**Gi Z**	**HotCold**	**Gi Z**	**HotCold**	**GiZ**	**HotCold**			**Gi Z**	**HotCold**
BAGERHAT	-0.911	Not significant	-0.844	Not significant	1.075	Not significant	0.043	Not significant	-0.306	Not significant	-0.235	Not significant	-0.007	Not significant	0.096	Not significant
BANDARBAN	2.247	Hot spot - 95% CI	4.531	Hot spot - 99% CI	0.523	Not significant	-0.721	Not significant	-0.084	Not significant	0.534	Not significant	0.178	Not significant	0.541	Not significant
BARGUNA	-0.652	Not significant	0.276	Not significant	1.229	Not significant	-0.883	Not significant	-0.270	Not significant	-0.223	Not significant	0.179	Not significant	-0.209	Not significant
BARISAL	0.059	Not significant	-0.210	Not significant	0.015	Not significant	0.152	Not significant	-0.432	Not significant	-0.474	Not significant	-0.021	Not significant	-0.366	Not significant
BHOLA	-0.552	Not significant	0.478	Not significant	1.595	Not significant	-0.614	Not significant	-0.225	Not significant	-0.197	Not significant	0.371	Not significant	-0.120	Not significant
BOGRA	-1.132	Not significant	-1.048	Not significant	-1.170	Not significant	-1.059	Not significant	-0.379	Not significant	-0.472	Not significant	-0.628	Not significant	-0.586	Not significant
BRAHAMANBARIA	-0.393	Not significant	-0.075	Not significant	0.082	Not significant	1.075	Not significant	-0.309	Not significant	-0.328	Not significant	-0.361	Not significant	-0.052	Not significant
CHANDPUR	-0.962	Not significant	-0.836	Not significant	1.496	Not significant	-0.223	Not significant	-0.318	Not significant	-0.304	Not significant	0.100	Not significant	-0.269	Not significant
CHITTAGONG	0.279	Not significant	2.781	Hot spot - 99% CI	-1.166	Not significant	-0.781	Not significant	-0.321	Not significant	-0.226	Not significant	-0.542	Not significant	-0.160	Not significant
CHUADANGA	-0.496	Not significant	0.163	Not significant	0.211	Not significant	-0.542	Not significant	-0.250	Not significant	-0.275	Not significant	-0.172	Not significant	-0.261	Not significant
COMILLA	0.272	Not significant	-0.171	Not significant	-0.344	Not significant	0.232	Not significant	-0.359	Not significant	-0.388	Not significant	-0.469	Not significant	-0.306	Not significant
COX'S BAZAR	1.094	Not significant	-0.444	Not significant	1.128	Not significant	-0.526	Not significant	-0.076	Not significant	0.541	Not significant	0.253	Not significant	0.292	Not significant
DHAKA	1.864	Hot spot - 90% CI	1.888	Hot spot - 90% CI	0.734	Not significant	3.616	Hot spot - 99% CI	2.481	Hot spot - 95% CI	0.269	Not significant	1.981	Hot spot - 95% CI	2.159	Hot spot - 95% CI
DINAJPUR	-1.066	Not significant	-1.251	Not significant	-1.553	Not significant	-1.162	Not significant	-0.385	Not significant	-0.501	Not significant	-0.792	Not significant	-0.696	Not significant
FARIDPUR	0.768	Not significant	-0.318	Not significant	0.398	Not significant	-0.108	Not significant	2.593	Hot spot - 99% CI	2.540	Hot spot - 95% CI	2.915	Hot spot - 99% CI	2.515	Hot spot - 95% CI

FENI	1.077	Not significant	-0.263	Not significant	1.619	Not significant	-0.566	Not significant	-0.129	Not significant	0.331	Not significant	0.273	Not significant	0.159	Not significant
GAIBANDHA	-0.083	Not significant	-0.638	Not significant	-0.821	Not significant	-1.047	Not significant	-0.376	Not significant	-0.466	Not significant	-0.699	Not significant	-0.608	Not significant
GAZIPUR	3.518	Hot spot - 99% CI	1.365	Not significant	1.082	Not significant	3.497	Hot spot - 99% CI	3.103	Hot spot - 99% CI	3.061	Hot spot - 99% CI	2.981	Hot spot - 99% CI	3.398	Hot spot - 99% CI
GOPALGANJ	-1.021	Not significant	-0.527	Not significant	1.663	Hot spot - 90% CI	0.250	Not significant	-0.325	Not significant	-0.186	Not significant	0.257	Not significant	-0.170	Not significant
HABIGANJ	-1.334	Not significant	-0.701	Not significant	-1.055	Not significant	0.209	Not significant	-0.337	Not significant	-0.422	Not significant	-0.746	Not significant	-0.601	Not significant
JAMALPUR	1.372	Not significant	-0.265	Not significant	-0.030	Not significant	0.232	Not significant	-0.374	Not significant	-0.456	Not significant	-0.528	Not significant	-0.425	Not significant
JESSORE	-0.817	Not significant	-0.827	Not significant	1.384	Not significant	0.092	Not significant	-0.309	Not significant	-0.296	Not significant	-0.143	Not significant	-0.112	Not significant
JHALOKATI	-0.935	Not significant	-0.531	Not significant	2.338	Hot spot - 95% CI	-0.654	Not significant	-0.204	Not significant	-0.053	Not significant	0.435	Not significant	0.157	Not significant
JHENAIDAH	-0.817	Not significant	0.399	Not significant	1.767	Hot spot - 90% CI	0.535	Not significant	-0.317	Not significant	-0.294	Not significant	-0.171	Not significant	-0.432	Not significant
JOYPURHAT	0.386	Not significant	-0.412	Not significant	-0.780	Not significant	-0.762	Not significant	-0.298	Not significant	-0.364	Not significant	-0.558	Not significant	-0.499	Not significant
KHAGRACHHARI	2.264	Hot spot - 95% CI	4.111	Hot spot - 99% CI	0.980	Not significant	-0.565	Not significant	-0.082	Not significant	0.490	Not significant	0.235	Not significant	0.169	Not significant
KHULNA	-0.667	Not significant	-0.249	Not significant	2.071	Hot spot - 95% CI	1.484	Not significant	-0.272	Not significant	-0.240	Not significant	-0.315	Not significant	-0.334	Not significant
KISHOREGANJ	-0.033	Not significant	-0.298	Not significant	-0.880	Not significant	1.737	Hot spot - 90% CI	-0.228	Not significant	-0.405	Not significant	-0.490	Not significant	-0.370	Not significant
KURIGRAM	-1.241	Not significant	-0.925	Not significant	-0.887	Not significant	-1.003	Not significant	-0.306	Not significant	-0.392	Not significant	-0.572	Not significant	-0.502	Not significant
KUSHTIA	-0.297	Not significant	-0.421	Not significant	-0.846	Not significant	-0.837	Not significant	-0.398	Not significant	-0.401	Not significant	-0.360	Not significant	-0.557	Not significant
LAKSHMIPUR	0.284	Not significant	-0.385	Not significant	2.204	Hot spot - 95% CI	0.559	Not significant	-0.241	Not significant	-0.187	Not significant	0.192	Not significant	-0.099	Not significant
LALMONIRHAT	-0.716	Not significant	-0.968	Not significant	-0.712	Not significant	-0.579	Not significant	-0.263	Not significant	-0.344	Not significant	-0.512	Not significant	-0.451	Not significant

MADARIPUR	-1.053	Not significant	-0.438	Not significant	1.625	Not significant	0.590	Not significant	-0.293	Not significant	-0.228	Not significant	0.150	Not significant	-0.194	Not significant
MAGURA	-0.998	Not significant	0.388	Not significant	1.878	Hot spot - 90% CI	1.660	Hot spot - 90% CI	-0.308	Not significant	-0.255	Not significant	-0.035	Not significant	-0.310	Not significant
MANIKGANJ	3.499	Hot spot - 99% CI	1.523	Not significant	0.758	Not significant	1.183	Not significant	3.052	Hot spot - 99% CI	3.055	Hot spot - 99% CI	3.205	Hot spot - 99% CI	3.075	Hot spot - 99% CI
MAULVIBAZAR	-0.802	Not significant	-0.666	Not significant	-0.616	Not significant	-0.797	Not significant	-0.208	Not significant	-0.269	Not significant	-0.448	Not significant	-0.388	Not significant
MEHERPUR	-0.269	Not significant	0.266	Not significant	0.181	Not significant	-0.396	Not significant	-0.206	Not significant	-0.210	Not significant	-0.231	Not significant	-0.253	Not significant
MUNSHIGANJ	2.487	Hot spot - 95% CI	0.852	Not significant	0.994	Not significant	1.209	Not significant	2.828	Hot spot - 99% CI	2.827	Hot spot - 99% CI	3.019	Hot spot - 99% CI	3.019	Hot spot - 99% CI
MYMENSINGH	1.323	Not significant	1.083	Not significant	-0.391	Not significant	3.942	Hot spot - 99% CI	-0.194	Not significant	-0.376	Not significant	-0.450	Not significant	-0.277	Not significant
NAOGAON	-0.126	Not significant	-0.584	Not significant	-0.886	Not significant	-0.683	Not significant	-0.335	Not significant	-0.379	Not significant	-0.513	Not significant	-0.553	Not significant
NARAIL	-1.338	Not significant	-0.083	Not significant	2.559	Hot spot - 95% CI	1.194	Not significant	-0.333	Not significant	-0.253	Not significant	-0.021	Not significant	-0.108	Not significant
NARAYANGANJ	2.248	Hot spot - 95% CI	0.652	Not significant	0.111	Not significant	2.207	Hot spot - 95% CI	3.216	Hot spot - 99% CI	3.022	Hot spot - 99% CI	3.094	Hot spot - 99% CI	3.146	Hot spot - 99% CI
NARSINGDI	0.391	Not significant	1.051	Not significant	-0.172	Not significant	3.856	Hot spot - 99% CI	-0.103	Not significant	-0.226	Not significant	-0.321	Not significant	-0.067	Not significant
NATORE	0.964	Not significant	0.292	Not significant	0.596	Not significant	-0.505	Not significant	-0.357	Not significant	-0.302	Not significant	-0.269	Not significant	-0.401	Not significant
NAWABGANJ	-0.402	Not significant	-0.356	Not significant	-0.298	Not significant	-0.485	Not significant	-0.191	Not significant	-0.195	Not significant	-0.164	Not significant	-0.256	Not significant
NETRAKONA	-0.167	Not significant	-0.201	Not significant	0.523	Not significant	0.927	Not significant	-0.203	Not significant	-0.220	Not significant	-0.369	Not significant	-0.233	Not significant
NILPHAMARI	-0.373	Not significant	-0.955	Not significant	-1.078	Not significant	-1.101	Not significant	-0.304	Not significant	-0.394	Not significant	-0.631	Not significant	-0.547	Not significant
NOAKHALI	1.827	Hot spot - 90% CI	-0.487	Not significant	1.299	Not significant	0.360	Not significant	-0.251	Not significant	0.070	Not significant	0.246	Not significant	-0.049	Not significant
PABNA	-0.492	Not significant	0.021	Not significant	0.789	Not significant	-0.664	Not significant	-0.319	Not significant	-0.346	Not significant	0.073	Not significant	-0.293	Not significant

PANCHAGARH	-0.047	Not significant	-0.567	Not significant	-1.195	Not significant	-0.728	Not significant	-0.260	Not significant	-0.337	Not significant	-0.545	Not significant	-0.449	Not significant
PATUAKHALI	-0.676	Not significant	-0.735	Not significant	2.013	Hot spot - 95% CI	-0.427	Not significant	-0.195	Not significant	-0.084	Not significant	0.304	Not significant	0.127	Not significant
PIROJPUR	-1.326	Not significant	-1.085	Not significant	0.847	Not significant	-0.678	Not significant	-0.302	Not significant	-0.248	Not significant	-0.050	Not significant	-0.208	Not significant
RAJBARI	-0.227	Not significant	0.627	Not significant	1.108	Not significant	-0.016	Not significant	-0.344	Not significant	-0.272	Not significant	0.236	Not significant	-0.204	Not significant
RAJSHAHI	-0.558	Not significant	-0.272	Not significant	-0.631	Not significant	-0.620	Not significant	-0.298	Not significant	-0.332	Not significant	-0.486	Not significant	-0.463	Not significant
RANGAMATI	0.541	Not significant	-0.802	Not significant	0.519	Not significant	-0.540	Not significant	-0.154	Not significant	0.327	Not significant	0.029	Not significant	0.080	Not significant
RANGPUR	-0.644	Not significant	-1.134	Not significant	-1.583	Not significant	-0.859	Not significant	-0.345	Not significant	-0.450	Not significant	-0.698	Not significant	-0.601	Not significant
SATKHIRA	-0.650	Not significant	0.401	Not significant	4.630	Hot spot - 99% CI	1.523	Not significant	-0.147	Not significant	-0.016	Not significant	0.091	Not significant	0.350	Not significant
SHARIATPUR	0.302	Not significant	-0.467	Not significant	2.087	Hot spot - 95% CI	0.434	Not significant	-0.229	Not significant	-0.150	Not significant	0.245	Not significant	-0.037	Not significant
SHERPUR	0.011	Not significant	-0.399	Not significant	0.517	Not significant	-0.793	Not significant	-0.151	Not significant	-0.155	Not significant	-0.192	Not significant	-0.092	Not significant
SIRAJGANJ	1.837	Hot spot - 90% CI	0.817	Not significant	0.350	Not significant	0.650	Not significant	-0.349	Not significant	-0.350	Not significant	-0.190	Not significant	-0.243	Not significant
SUNAMGANJ	-1.145	Not significant	-0.592	Not significant	-0.528	Not significant	0.525	Not significant	-0.289	Not significant	-0.359	Not significant	-0.604	Not significant	-0.466	Not significant
SYLHET	-0.935	Not significant	-0.709	Not significant	-1.493	Not significant	-0.837	Not significant	-0.265	Not significant	-0.349	Not significant	-0.602	Not significant	-0.513	Not significant
TANGAIL	2.747	Hot spot - 99% CI	0.951	Not significant	1.521	Not significant	1.804	Hot spot - 90% CI	3.246	Hot spot - 99% CI	3.076	Hot spot - 99% CI	3.440	Hot spot - 99% CI	3.263	Hot spot - 99% CI
THAKURGAON	0.268	Not significant	-0.525	Not significant	-1.060	Not significant	-0.793	Not significant	-0.209	Not significant	-0.270815577	Not significant	-0.453	Not significant	-0.368	Not significant

GiZ: Getis-OrdG_i^*statistic for local clustering

Table S11. Spatial autocorrelation or cluster analysis of yearly dengue cases of Bangladesh from 2017 to 2024..

**Year**	**2017**		**2018**		**2019**		**2020**		**2021**		**2022**		**2023**		**2024**	
**District**	**Lisa Z**	**LisaCat**	**Lisa Z**	**LisaCat**	**Lisa Z**	**LisaCat**	**Lisa Z**	**LisaCat**	**Lisa Z**	**LisaCat**	**Lisa Z**	**LisaCat**	**Lisa Z**	**LisaCat**	**Lisa Z**	**LisaCat**
BAGERHAT	0.227	Not Significant	0.454	Not Significant	-0.589	Not Significant	-0.266	Not Significant	0.223	Not Significant	0.232	Not Significant	-0.250	Not Significant	-0.581	Not Significant
BANDARBAN	-1.472	Not Significant	-2.780	Low-Low 99%	-0.483	Not Significant	-0.042	Not Significant	0.089	Not Significant	-0.238	Not Significant	-0.499	Not Significant	-0.971	Not Significant
BARGUNA	0.113	Not Significant	-0.005	Not Significant	-0.285	Not Significant	-0.024	Not Significant	0.173	Not Significant	0.177	Not Significant	-0.265	Not Significant	-0.617	Not Significant
BARISAL	-0.192	Not Significant	0.155	Not Significant	-0.494	Not Significant	-0.275	Not Significant	0.120	Not Significant	-0.031	Not Significant	-0.326	Not Significant	-0.698	Not Significant
BHOLA	-0.207	Not Significant	-0.134	Not Significant	-0.105	Not Significant	-0.258	Not Significant	0.170	Not Significant	0.215	Not Significant	-0.361	Not Significant	-0.310	Not Significant
BOGRA	-1.048	Not Significant	-0.210	Not Significant	-0.479	Not Significant	-0.223	Not Significant	0.249	Not Significant	0.328	Not Significant	0.143	Not Significant	0.213	Not Significant
BRAHAMANBARIA	-0.001	Not Significant	0.131	Not Significant	-0.192	Not Significant	-0.628	Not Significant	0.235	Not Significant	0.330	Not Significant	0.166	Not Significant	-0.348	Not Significant
CHANDPUR	-1.278	Not Significant	-0.148	Not Significant	0.834	Not Significant	-0.540	Not Significant	0.160	Not Significant	0.226	Not Significant	-0.237	Not Significant	-0.396	Not Significant
CHITTAGONG	0.078	Not Significant	-0.244	Not Significant	-2.253	Low-Low 95%	-0.096	Not Significant	-0.040	Not Significant	-0.669	Not Significant	-1.395	Not Significant	-0.899	Not Significant
CHUADANGA	0.113	Not Significant	-0.011	Not Significant	-0.295	Not Significant	-0.043	Not Significant	0.247	Not Significant	0.353	Not Significant	-0.038	Not Significant	0.072	Not Significant
COMILLA	-0.117	Not Significant	0.122	Not Significant	-0.283	Not Significant	-0.286	Not Significant	0.174	Not Significant	0.255	Not Significant	-0.294	Not Significant	-0.377	Not Significant
COX'S BAZAR	0.627	Not Significant	-1.303	Not Significant	-0.873	Not Significant	-0.031	Not Significant	0.031	Not Significant	0.814	Not Significant	-0.279	Not Significant	1.300	Not Significant
DHAKA	3.654	High-High 99%	2.055	High-High 95%	0.427	Not Significant	0.025	Not Significant	-6.133	Low-Low 99%	-7.090	Low-Low 99%	3.081	High-High 99%	4.945	High-High 99%
DINAJPUR	-0.610	Not Significant	0.256	Not Significant	0.618	Not Significant	0.100	Not Significant	0.242	Not Significant	0.392	Not Significant	0.439	Not Significant	0.393	Not Significant
FARIDPUR	-0.434	Not Significant	-0.063	Not Significant	0.029	Not Significant	-0.322	Not Significant	-1.252	Not Significant	-0.399	Not Significant	1.126	Not Significant	-0.264	Not Significant

FENI	0.464	Not Significant	0.148	Not Significant	-0.446	Not Significant	-0.230	Not Significant	0.123	Not Significant	-0.136	Not Significant	-0.491	Not Significant	-0.707	Not Significant
GAIBANDHA	-0.141	Not Significant	0.402	Not Significant	0.350	Not Significant	-0.042	Not Significant	0.256	Not Significant	0.398	Not Significant	0.337	Not Significant	0.398	Not Significant
GAZIPUR	3.541	High-High 99%	1.985	High-High 95%	0.005	Not Significant	6.842	High-High 99%	3.107	High-High 99%	-0.180	Not Significant	1.243	Not Significant	0.560	Not Significant
GOPALGANJ	0.006	Not Significant	0.297	Not Significant	-0.371	Not Significant	-0.226	Not Significant	0.222	Not Significant	0.206	Not Significant	-0.353	Not Significant	-0.226	Not Significant
HABIGANJ	0.249	Not Significant	0.393	Not Significant	0.554	Not Significant	-0.332	Not Significant	0.245	Not Significant	0.396	Not Significant	0.541	Not Significant	0.469	Not Significant
JAMALPUR	-0.675	Not Significant	0.119	Not Significant	-0.091	Not Significant	-0.326	Not Significant	0.224	Not Significant	0.334	Not Significant	0.089	Not Significant	-0.123	Not Significant
JESSORE	0.087	Not Significant	-0.621	Not Significant	3.694	High-High 99%	-0.256	Not Significant	0.165	Not Significant	0.107	Not Significant	-0.250	Not Significant	-0.390	Not Significant
JHALOKATI	0.366	Not Significant	0.467	Not Significant	-2.694	Low-Low 99%	0.034	Not Significant	0.205	Not Significant	0.142	Not Significant	-0.948	Not Significant	-0.781	Not Significant
JHENAIDAH	0.087	Not Significant	0.046	Not Significant	-0.089	Not Significant	-0.355	Not Significant	0.224	Not Significant	0.284	Not Significant	-0.219	Not Significant	-0.007	Not Significant
JOYPURHAT	-0.305	Not Significant	0.292	Not Significant	0.512	Not Significant	0.034	Not Significant	0.252	Not Significant	0.385	Not Significant	0.449	Not Significant	0.481	Not Significant
KHAGRACHHARI	-1.789	Low-Low 90%	-3.126	Low-Low 99%	-1.065	Not Significant	-0.154	Not Significant	0.107	Not Significant	-0.453	Not Significant	-0.640	Not Significant	-0.791	Not Significant
KHULNA	0.066	Not Significant	0.185	Not Significant	4.415	High-High 99%	-0.569	Not Significant	0.159	Not Significant	0.106	Not Significant	-0.414	Not Significant	-1.620	Not Significant
KISHOREGANJ	-0.160	Not Significant	0.033	Not Significant	-0.550	Not Significant	1.702	High-High 90%	0.125	Not Significant	0.259	Not Significant	0.078	Not Significant	-0.218	Not Significant
KURIGRAM	0.269	Not Significant	0.654	Not Significant	0.502	Not Significant	-0.130	Not Significant	0.260	Not Significant	0.413	Not Significant	0.400	Not Significant	0.388	Not Significant
KUSHTIA	-0.214	Not Significant	-0.220	Not Significant	-0.760	Not Significant	-0.231	Not Significant	0.225	Not Significant	0.211	Not Significant	-0.221	Not Significant	-0.186	Not Significant
LAKSHMIPUR	-0.315	Not Significant	0.325	Not Significant	-0.938	Not Significant	-0.348	Not Significant	0.197	Not Significant	0.217	Not Significant	-0.148	Not Significant	-0.302	Not Significant

LALMONIRHAT	0.239	Not Significant	0.777	Not Significant	0.546	Not Significant	-0.028	Not Significant	0.261	Not Significant	0.418	Not Significant	0.491	Not Significant	0.517	Not Significant
MADARIPUR	-0.238	Not Significant	0.234	Not Significant	-0.155	Not Significant	-0.390	Not Significant	0.179	Not Significant	0.174	Not Significant	-0.234	Not Significant	-0.341	Not Significant
MAGURA	0.265	Not Significant	-0.070	Not Significant	-0.473	Not Significant	-0.501	Not Significant	0.223	Not Significant	0.241	Not Significant	-0.250	Not Significant	-0.010	Not Significant
MANIKGANJ	-1.567	Not Significant	0.152	Not Significant	0.660	Not Significant	-0.498	Not Significant	-1.400	Not Significant	-1.217	Not Significant	5.298	High-High 99%	2.076	High-High 95%
MAULVIBAZAR	0.398	Not Significant	0.673	Not Significant	0.713	Not Significant	0.182	Not Significant	0.254	Not Significant	0.409	Not Significant	0.603	Not Significant	0.621	Not Significant
MEHERPUR	0.018	Not Significant	-0.089	Not Significant	-0.270	Not Significant	-0.040	Not Significant	0.242	Not Significant	0.332	Not Significant	-0.004	Not Significant	-0.024	Not Significant
MUNSHIGANJ	-1.083	Not Significant	-0.206	Not Significant	-0.304	Not Significant	-0.443	Not Significant	-1.551	Not Significant	-1.367	Not Significant	-1.942	Low-Low 90%	-2.831	Low-Low 99%
MYMENSINGH	0.366	Not Significant	-0.322	Not Significant	-0.492	Not Significant	-1.481	Not Significant	0.070	Not Significant	0.147	Not Significant	-0.208	Not Significant	-0.457	Not Significant
NAOGAON	-0.129	Not Significant	0.311	Not Significant	0.360	Not Significant	-0.051	Not Significant	0.239	Not Significant	0.339	Not Significant	0.261	Not Significant	0.323	Not Significant
NARAIL	0.059	Not Significant	0.131	Not Significant	-0.467	Not Significant	0.399	Not Significant	0.210	Not Significant	0.194	Not Significant	-0.243	Not Significant	-0.333	Not Significant
NARAYANGANJ	-0.022	Not Significant	0.417	Not Significant	-0.111	Not Significant	-0.161	Not Significant	-2.077	Low-Low 95%	-0.667	Not Significant	-2.113	Low-Low 95%	3.122	High-High 99%
NARSINGDI	-0.247	Not Significant	-0.340	Not Significant	-0.065	Not Significant	1.456	Not Significant	0.088	Not Significant	0.252	Not Significant	-0.212	Not Significant	-0.460	Not Significant
NATORE	-0.327	Not Significant	-0.018	Not Significant	-0.459	Not Significant	-0.221	Not Significant	0.241	Not Significant	0.271	Not Significant	-0.031	Not Significant	0.116	Not Significant
NAWABGANJ	0.113	Not Significant	0.402	Not Significant	0.281	Not Significant	0.009	Not Significant	0.235	Not Significant	0.322	Not Significant	0.053	Not Significant	0.260	Not Significant
NETRAKONA	-0.078	Not Significant	0.220	Not Significant	-0.614	Not Significant	-0.483	Not Significant	0.197	Not Significant	0.290	Not Significant	0.230	Not Significant	-0.016	Not Significant
NILPHAMARI	-0.087	Not Significant	0.612	Not Significant	0.630	Not Significant	-0.070	Not Significant	0.253	Not Significant	0.408	Not Significant	0.459	Not Significant	0.396	Not Significant
NOAKHALI	-0.902	Not Significant	0.162	Not Significant	-0.171	Not Significant	-0.330	Not Significant	0.176	Not Significant	0.049	Not Significant	-0.415	Not Significant	-0.372	Not Significant

PABNA	-0.753	Not Significant	0.103	Not Significant	-0.086	Not Significant	-0.258	Not Significant	0.223	Not Significant	0.099	Not Significant	-0.279	Not Significant	-0.214	Not Significant
PANCHAGARH	-0.154	Not Significant	0.493	Not Significant	1.106	Not Significant	0.067	Not Significant	0.257	Not Significant	0.412	Not Significant	0.560	Not Significant	0.481	Not Significant
PATUAKHALI	-0.694	Not Significant	-1.914	Low-Low 90%	-0.210	Not Significant	-0.150	Not Significant	0.092	Not Significant	0.151	Not Significant	-0.001	Not Significant	-0.426	Not Significant
PIROJPUR	0.081	Not Significant	0.125	Not Significant	0.319	Not Significant	-0.103	Not Significant	0.217	Not Significant	0.179	Not Significant	-0.282	Not Significant	-0.324	Not Significant
RAJBARI	-0.127	Not Significant	0.122	Not Significant	-0.414	Not Significant	-0.254	Not Significant	0.227	Not Significant	0.247	Not Significant	-0.290	Not Significant	-0.135	Not Significant
RAJSHAHI	-0.127	Not Significant	0.126	Not Significant	-0.226	Not Significant	-0.177	Not Significant	0.210	Not Significant	0.223	Not Significant	-0.399	Not Significant	-0.103	Not Significant
RANGAMATI	0.309	Not Significant	-4.937	Low-Low 99%	-0.612	Not Significant	-0.069	Not Significant	0.157	Not Significant	-0.206	Not Significant	-0.303	Not Significant	-0.598	Not Significant
RANGPUR	0.033	Not Significant	0.640	Not Significant	-0.379	Not Significant	0.034	Not Significant	0.251	Not Significant	0.406	Not Significant	0.358	Not Significant	0.450	Not Significant
SATKHIRA	-0.238	Not Significant	-0.104	Not Significant	1.691	High-High 90%	-0.524	Not Significant	0.174	Not Significant	0.110	Not Significant	-0.381	Not Significant	-1.078	Not Significant
SHARIATPUR	-0.184	Not Significant	0.203	Not Significant	-0.281	Not Significant	-0.036	Not Significant	0.171	Not Significant	0.202	Not Significant	-0.389	Not Significant	-0.374	Not Significant
SHERPUR	-0.165	Not Significant	0.405	Not Significant	-0.662	Not Significant	0.083	Not Significant	0.181	Not Significant	0.263	Not Significant	0.018	Not Significant	-0.223	Not Significant
SIRAJGANJ	-0.474	Not Significant	-0.265	Not Significant	0.019	Not Significant	-0.413	Not Significant	0.231	Not Significant	0.307	Not Significant	-0.226	Not Significant	-0.249	Not Significant
SUNAMGANJ	0.235	Not Significant	0.193	Not Significant	0.347	Not Significant	-0.366	Not Significant	0.247	Not Significant	0.384	Not Significant	0.533	Not Significant	0.444	Not Significant
SYLHET	0.366	Not Significant	0.490	Not Significant	0.115	Not Significant	0.116	Not Significant	0.249	Not Significant	0.393	Not Significant	0.549	Not Significant	0.587	Not Significant
TANGAIL	4.435	High-High 99%	1.303	Not Significant	0.069	Not Significant	2.116	High-High 95%	-1.762	Low-Low 90%	-1.759	Low-Low 90%	-2.235	Low-Low 95%	0.250	Not Significant
THAKURGAON	-0.305	Not Significant	0.314	Not Significant	0.991	Not Significant	0.083	Not Significant	0.255	Not Significant	0.408	Not Significant	0.560	Not Significant	0.516	Not Significant

LisaZ: Local Indicator of Spatial Association (Z-score) or Local Moran's I Z-score for location iii

Table S12.DENV distribution summary from 2017 to 2024 in Bangladesh.

DENV type	Year	Min	Q1	Median	Q3	Max	Mean± SD	Percentage
**DENV1**	2017	0	0	1	2	14	2 ± 3	72%
	2018	0	1	2	5	50	5 ± 8	66%
	2019	24	107	306	467	2034	387 ± 395	55%
	2020	0	1	2	4	50	5 ± 9	51%
	2021	0	5	13	38	12285	231 ± 1533	53%
	2022	0	30	67	216	20493	507 ± 2566	53%
	2023	53	469	1139	2464	58881	2610 ± 7333	53%
	2024	3	120	273	708	21863	822 ± 2730	53%
**DENV2**	2017	0	0	1	3	22	3 ± 4	108%
	2018	0	1	2	4	43	4 ± 7	52%
	2019	0	2	6	9	41	8 ± 8	1%
	2020	0	0	0	0	0	0 ± 0	0%
	2021	0	0	0	0	0	0 ± 0	0%
	2022	0	0	0	0	0	0 ± 0	0%
	2023	0	0	0	0	0	0 ± 0	0%
	2024	0	0	0	0	0	0 ± 0	0%
**DENV3**	2017	0	0	0	0	0	0 ± 0	0%
	2018	0	1	1	3	33	3 ± 5	39%
	2019	42	192	550	841	3661	697 ± 711	100%
	2020	0	2	4	8	97	10 ± 17	102%
	2021	0	10	25	73	23625	444 ± 2948	103%
	2022	0	44	98	316	29952	741 ± 3751	78%
	2023	75	667	1621	3506	83792	3714 ± 10435	76%
	2024	4	171	389	1008	31113	1170 ± 3886	76%
**DENV4**	2017	0	0	0	0	0	0 ± 0	0%
	2018	0	0	0	0	0	0 ± 0	0%
	2019	0	0	0	0	0	0 ± 0	0%

2020	0	0	0	0	0	0 ± 0	0%
2021	0	0	0	0	0	0 ± 0	0%
2022	0	14	31	100	9458	234 ± 1184	25%
2023	27	234	570	1232	29441	1305 ± 3666	27%
2024	1	60	137	354	10932	411 ± 1365	27%

Min: Minimum; Q1: First quartile; Q3: 3rd quartile; Max: Maximum; SD: Standard deviation

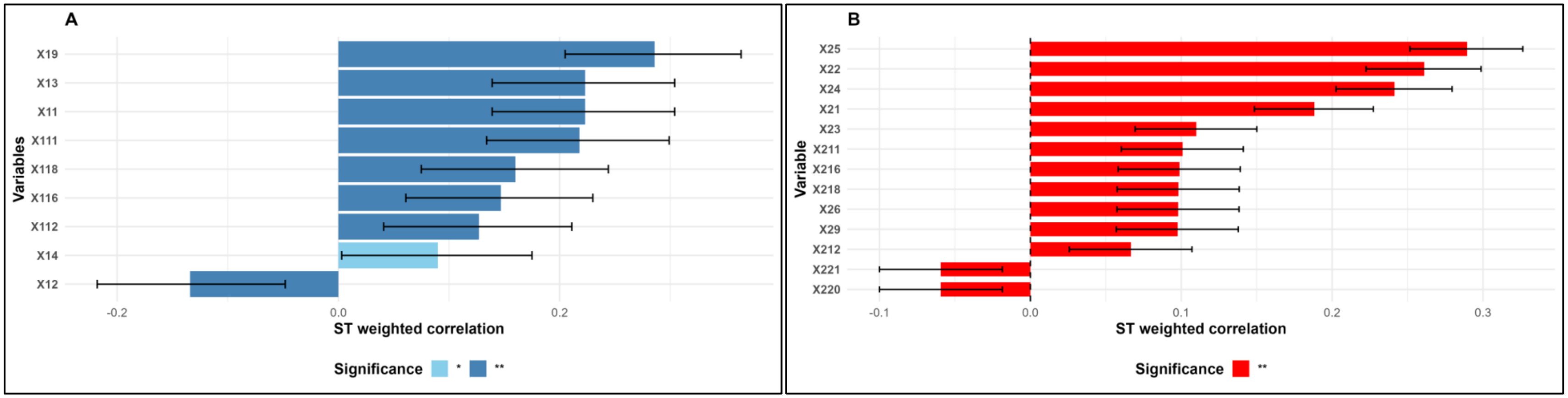

Figure S6. Significant spatio-temporal weighted correlations between A) yearly dengue cases, B) monthly cases and the associated variables. X11: Yearly average temperature at 2 meters (°C); X12: Minimum yearly temperature at 2 meters (°C); X13: Maximum yearly temperature at 2 meters (°C); X14: Yearly relative humidity at 2 meters (%); X15: Yearly rainfall corrected (mm/day); X16: Yearly surface pressure (kPa); X19: Yearly poverty head-count ratio (% of population); X111: Yearly population growth (%); X112: Yearly average household size; X116: Yearly domestic general government health expenditure (%); X118: Yearly UHC Service Coverage Index (SDG 3.8.1); X120: Yearly arable land; X121: Yearly agriculture land; X21: Monthly average temperature at 2 meters (°C); X22: Minimum monthly temperature at 2 meters (°C); X23: Maximum monthly temperature at 2 meters (°C); X24: Monthly relative humidity at 2 meters (%); X25: Monthly rainfall corrected (mm/day); X26: Monthly surface pressure (kPa); X27: Monthly GDP (Billion US$); X28: Total monthly population (each district); X29: Monthly poverty head-count ratio (% of population); X210: Monthly adult literacy rate (%); X211: Monthly population growth (%); X212: Average monthly household size; X213: Monthly access to electricity (% of population); X214: Total number of hospital beds (Monthly); X215: Number of physicians (Monthly); X216: Monthly domestic general government health expenditure (%); X217: Monthly density of nursing and midwifery personnel (per 10,000 population); X218: Monthly UHC Service Coverage Index (SDG 3.8.1); X219: Monthly land use; X220: Arable land (Monthly); X221: Agriculture land (Monthly).

Table S13. ST weighted correlation between yearly dengue cases and the associated variables.

Variable	*R* (95% CI)	P_value
X11	0.223 (0.139, 0.304)	<0.01**
X12	-0.134 (-0.218, -0.048)	<0.01**
X13	0.223 (0.139, 0.304)	<0.01**
X14	0.09 (0.003, 0.175)	<0.05*
X15	0.055 (-0.032, 0.141)	0.216
X16	0.086 (-0.001, 0.171)	0.052
X19	0.286 (0.205, 0.364)	<0.01**
X111	0.218 (0.134, 0.299)	<0.01**
X112	0.127 (0.041, 0.211)	<0.01**
X116	0.147 (0.061, 0.23)	<0.01**
X118	0.16 (0.075, 0.244)	<0.01**
X120	0.022 (-0.065, 0.108)	0.625
X121	-0.064 (-0.15, 0.022)	0.146

ST = Spatio-Temporal; R = Spatially weighted correlation coefficient; CI = 95% Confidence Interval; $P < 0.05$ (*), **P** < 0.01 (**). Variables correspond to yearly environmental, socio-demographic, and healthcare indicators in Bangladesh: X11: Yearly average temperature at 2 meters (°C); X12: Minimum yearly temperature at 2 meters (°C); X13: Maximum yearly temperature at 2 meters (°C); X14: Yearly relative humidity at 2 meters (%); X15: Yearly rainfall corrected (mm/day); X16: Yearly surface pressure (kPa); X19: Yearly poverty head-count ratio (% of population); X111: Yearly population growth (%); X112: Yearly average household size; X116: Yearly domestic general government health expenditure (%); X118: Yearly UHC Service Coverage Index (SDG 3.8.1); X120: Yearly arable land; X121: Yearly agriculture land.

Table S14. ST weighted correlation between monthly dengue cases and the associated variables.

Variable	R (95% **CI)**	P_value_star
X21	0.188 (0.1485, 0.2273)	<0.01**
X22	0.261 (0.2225, 0.2987)	<0.01**
X23	0.11 (0.0694, 0.1501)	<0.01**
X24	0.241 (0.2026, 0.2795)	<0.01**
X25	0.29 (0.2516, 0.3264)	<0.01**
X26	0.098 (0.0574, 0.1383)	<0.01**
X29	0.098 (0.0569, 0.1378)	<0.01**
X211	0.101 (0.0603, 0.1411)	<0.01**

X212	0.067 (0.0258, 0.1071)	<0.01**
X216	0.099 (0.0582, 0.1391)	<0.01**
X218	0.098 (0.0575, 0.1384)	<0.01**
X220	-0.059 (-0.1, -0.0186)	<0.01**
X221	-0.059 (-0.1, -0.0186)	<0.01**

ST = Spatio-Temporal; R = Spatially weighted correlation coefficient; CI = 95% Confidence Interval; $P < 0.05$ (*), **P** < 0.01 (**). Variables correspond to monthly environmental, socio-demographic, healthcare, and land-use indicators in Bangladesh: X21: Monthly average temperature at 2 meters (°C); X22: Minimum monthly temperature at 2 meters (°C); X23: Maximum monthly temperature at 2 meters (°C); X24: Monthly relative humidity at 2 meters (%); X25: Monthly rainfall corrected (mm/day); X26: Monthly surface pressure (kPa); X27: Monthly GDP (Billion US$); X28: Total monthly population (each district); X29: Monthly poverty head-count ratio (% of population); X210: Monthly adult literacy rate (%); X211: Monthly population growth (%); X212: Average monthly household size; X213: Monthly access to electricity (% of population); X214: Total number of hospital beds (Monthly); X215: Number of physicians (Monthly); X216: Monthly domestic general government health expenditure (%); X217: Monthly density of nursing and midwifery personnel (per 10,000 population); X218: Monthly UHC Service Coverage Index (SDG 3.8.1); X219: Monthly land use; X220: Arable land (Monthly); X221: Agriculture land (Monthly).

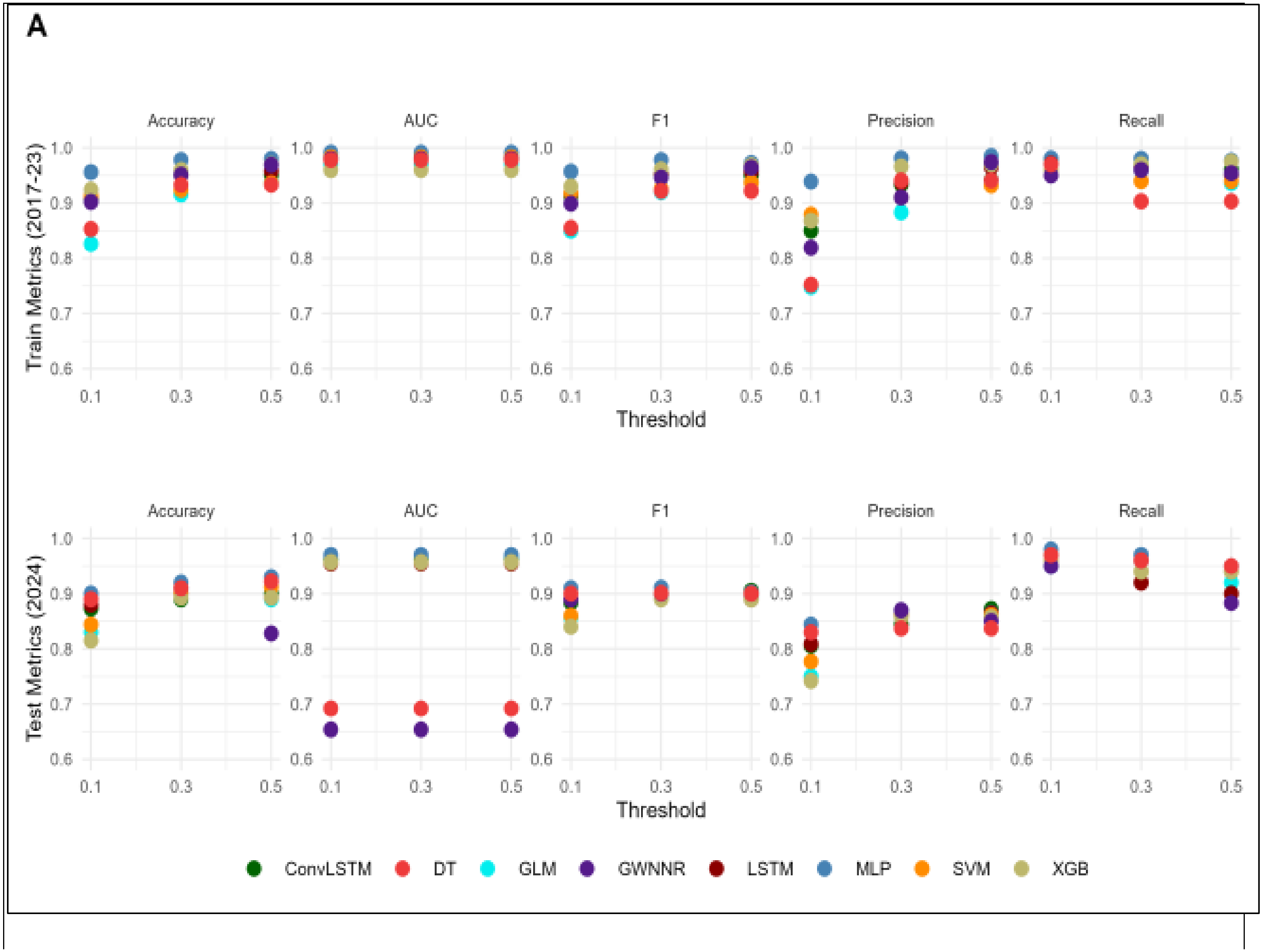
A
Accuracy
AUC
F1
Precision
Recall
Train Metrics (2017-23)
Test Metrics (2024)
Threshold
0.1
0.3
0.5
0.6
0.7
0.8
0.9
1.0
ConvLSTM
DT
GLM
GWNNR
LSTM
MLP
SVM
XGB

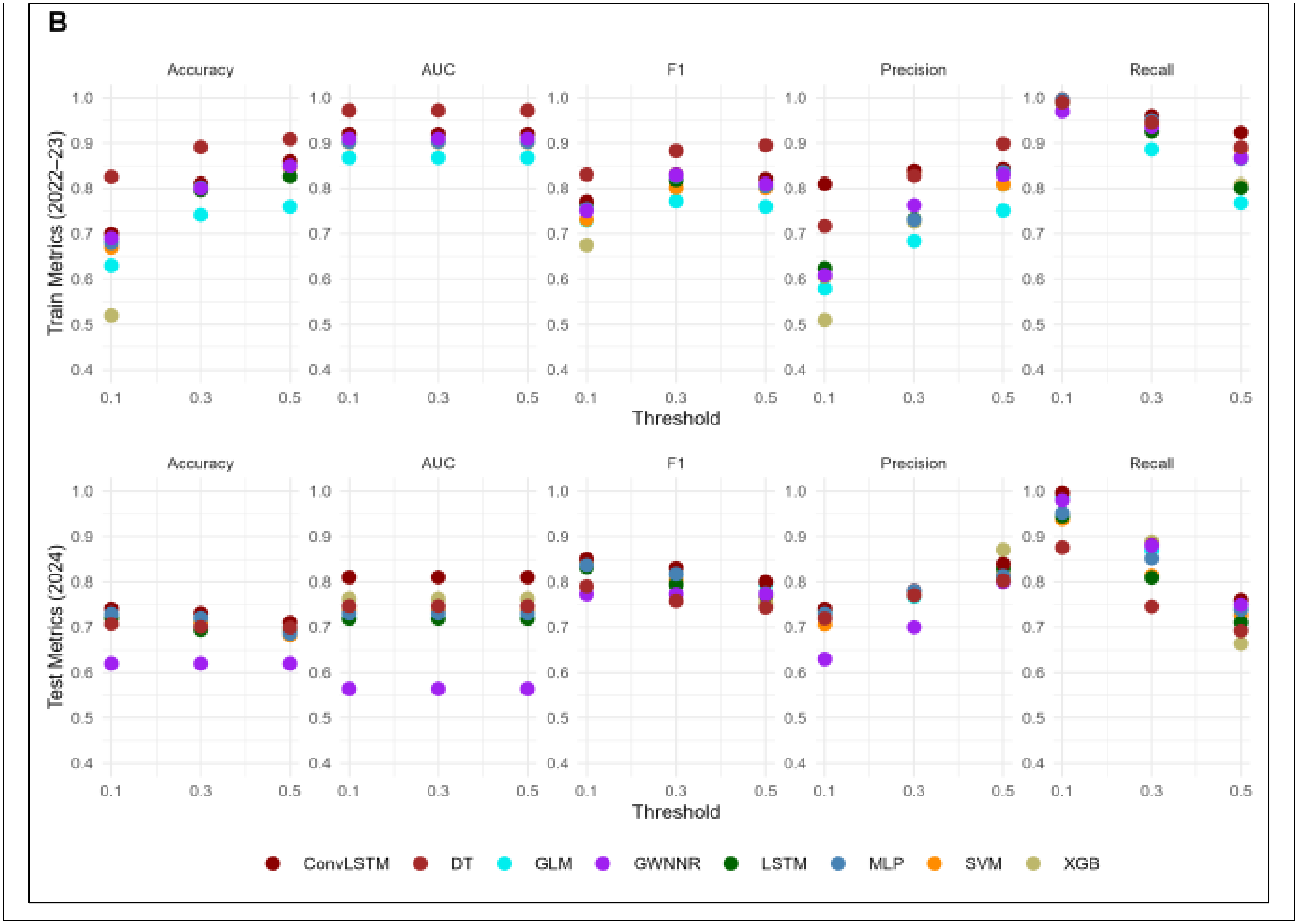

B
Accuracy
AUC
F1
Precision
Recall
Train Metrics (2022–23)
Test Metrics (2024)
Threshold
1.0
0.9
0.8
0.7
0.6
0.5
0.4
0.1
0.3
0.5
ConvLSTM
DT
GLM
GWNNR
LSTM
MLP
SVM
XGB

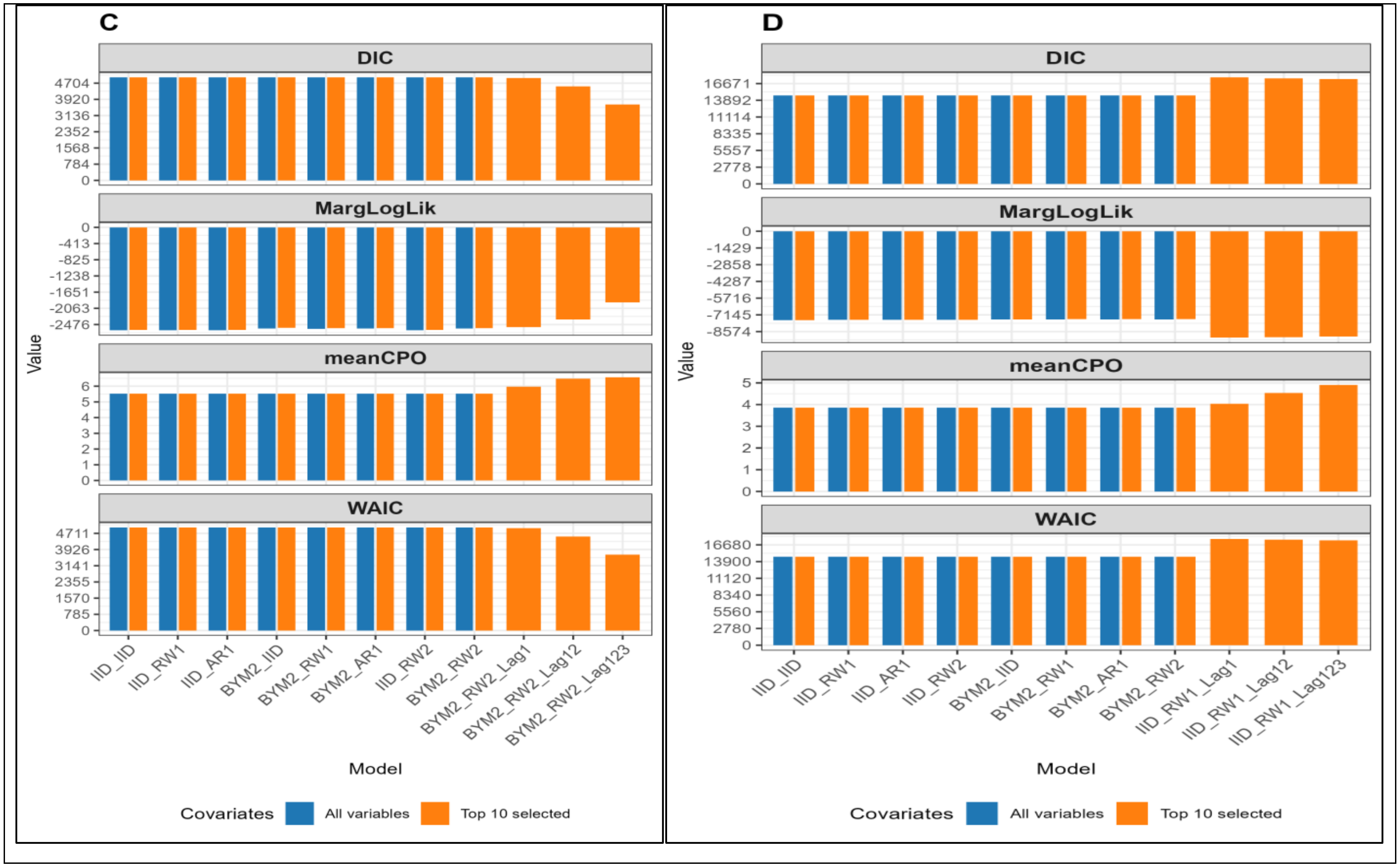

Figure S7. Model comparison matrices. Machine learning and deep learning model comparison for A) yearly dengue dataset, B) monthly dengue dataset. Baysian spatio-temporal model comparison for C) yearly dengue and D) monthly dataset; GLM: Generalized Linear Model; ConvLSTM: Convolutional Long Short-Term Memory (2D); MLP: Multi-Layer Perceptron; LSTM: Long Short-Term Memory (two-sequence); SVM: Support Vector Machine; XGB: eXtreme Gradient Boosting; GWNNR: Geographically Weighted Neural Network Regression; DT: Decision Tree; IID: Independent and Identically Distributed; RW1: Random Walk (order 1); AR1: Autoregressive (order 1); RW2: Random Walk (order 2); BYM2: Besag–York–Mollié 2 model.

Table S15. Performance matrices of yearly dengue cases 2017-24 (train 2017-2023) and (test set 2024)

**Dataset_1**	**Train**						**Test**				
**Models**	**Threshold**	**Accuracy**	**Precision**	**Recall**	**F1**	**ROC-AUC**	**Accuracy**	**Precision**	**Recall**	**F1**	**ROC-AUC**
GLM	0.1	0.826	0.748	0.97	0.85	0.97	0.83	0.75	0.95	0.857	0.96
	0.3	0.916	0.883	0.96	0.92	0.97	0.91	0.862	0.96	0.91	0.963
	0.5	0.938	0.941	0.936	0.938	0.97	0.89	0.87	0.92	0.895	0.967
ConvLSTM	0.1	0.907	0.85	0.98	0.913	0.98	0.873	0.806	0.97	0.884	0.962
	0.3	0.951	0.934	0.97	0.952	0.98	0.89	0.844	0.96	0.899	0.962
	0.5	0.951	0.942	0.961	0.951	0.98	0.9	0.872	0.94	0.905	0.962
**MLP**	**0.1**	**0.956**	**0.939**	**0.981**	**0.957**	**0.991**	**0.9**	**0.844**	**0.98**	**0.91**	**0.97**
	**0.3**	**0.978**	**0.981**	**0.98**	**0.978**	**0.991**	**0..920**	**0.87**	**0.97**	**0.911**	**0.97**
	**0.5**	**0.98**	**0.985**	**0.977**	**0.972**	**0.991**	**0..930**	**0.865**	**0.95**	**0.902**	**0.97**
LSTM	0.1	0.911	0.868	0.971	0.917	0.98	0.88	0.809	0.96	0.89	0.955
	0.3	0.951	0.938	0.966	0.952	0.98	0.9	0.861	0.92	0.9	0.955
	0.5	0.958	0.965	0.951	0.958	0.98	0.893	0.864	0.9	0.89	0.955
SVM	0.1	0.914	0.879	0.96	0.918	0.983	0.844	0.777	0.96	0.86	0.957
	0.3	0.924	0.91	0.94	0.926	0.983	0.902	0.86	0.94	0.9	0.957
	0.5	0.936	0.932	0.94	0.937	0.983	0.912	0.861	0.94	0.9	0.957
XGB	0.1	0.924	0.868	0.96	0.93	0.96	0.815	0.742	0.96	0.84	0.957
	0.3	0.96	0.966	0.97	0.961	0.96	0.893	0.857	0.94	0.89	0.957
	0.5	0.97	0.97	0.975	0.968	0.96	0.893	0.857	0.94	0.89	0.957
GWNNR	0.1	0.902	0.819	0.950	0.899	0.980	0.890	0.830	0.950	0.890	0.654
	0.3	0.951	0.910	0.960	0.946	0.980	0.910	0.870	0.960	0.900	0.654
	0.5	0.969	0.974	0.954	0.964	0.980	0.828	0.850	0.883	0.900	0.654
DT	0.1	0.853	0.752	0.970	0.855	0.978	0.890	0.830	0.970	0.900	0.692
	0.3	0.933	0.941	0.903	0.922	0.978	0.910	0.837	0.960	0.901	0.692
	0.5	0.933	0.941	0.903	0.922	0.978	0.922	0.837	0.950	0.900	0.692

GLM: Generalized Linear Model; ConvLSTM: Convolutional Long Short-Term Memory (2D); MLP: Multi-Layer Perceptron; LSTM: Long Short-Term Memory (with 2 time sequence); SVM: Support Vector Machine; XGB: eXtreme Gradient Boosting;. GWNNR: Geographically Weighted Neural Network Regression; DT: Decision Tree.

Table S16. Performance matrices of monthly dengue cases 2022-24 (train January 2022- December 2023) and (test set January 2024 to December 2024)

**Dataset_2**	**Train**						**Test**				
**Models**	**Threshold**	**Accuracy**	**Precision**	**Recall**	**F1**	**ROC_AUC**	**Accuracy**	**Precision**	**Recall**	**F1**	**ROC_AUC**
XGB	0.1	0.52	0.51	0.988	0.675	0.901	0.729	0.729	0.98	0.843	0.763
	0.3	0.801	0.726	0.941	0.827	0.901	0.704	0.78	0.889	0.829	0.763
	0.5	0.85	0.808	0.809	0.81	0.901	0.684	0.871	0.664	0.754	0.763
GLM	0.1	0.63	0.579	0.988	0.73	0.868	0.727	0.732	0.986	0.84	0.722
	0.3	0.742	0.684	0.886	0.772	0.868	0.714	0.768	0.87	0.816	0.722
	0.5	0.76	0.752	0.768	0.76	0.868	0.7	0.808	0.74	0.796	0.722
svm	0.1	0.67	0.606	0.989	0.732	0.91	0.72	0.706	0.938	0.843	0.738
	0.3	0.806	0.837	0.941	0.802	0.91	0.711	0.771	0.814	0.804	0.738
	0.5	0.847	0.81	0.887	0.801	0.91	0.682	0.817	0.727	0.769	0.738
LSTM	0.1	0.69	0.624	0.991	0.766	0.919	0.721	0.739	0.945	0.832	0.719
	0.3	0.796	0.733	0.926	0.819	0.919	0.694	0.78	0.809	0.794	0.719
	0.5	0.827	0.842	0.801	0.821	0.919	0.691	0.83	0.711	0.771	0.719
**ConvLSTM**	**0.1**	**0.7**	**0.81**	**0.996**	**0.771**	**0.921**	**0.741**	**0.741**	**0.996**	**0.851**	**0.81**
	**0.3**	**0.811**	**0.84**	**0.96**	**0.831**	**0.921**	**0.731**	**0.781**	**0.881**	**0.831**	**0.81**
	**0.5**	**0.86**	**0.844**	**0.924**	**0.821**	**0.921**	**0.711**	**0.84**	**0.76**	**0.8**	**0.81**
MLP	0.1	0.68	0.608	0.995	0.755	0.903	0.73	0.73	0.952	0.837	0.731
	0.3	0.803	0.731	0.951	0.827	0.903	0.721	0.78	0.852	0.817	0.731
	0.5	0.85	0.837	0.866	0.805	0.903	0.686	0.813	0.739	0.775	0.731
GWNNR	0.1	0.690	0.609	0.970	0.751	0.910	0.620	0.630	0.980	0.773	0.564
	0.3	0.800	0.763	0.936	0.830	0.910	0.620	0.700	0.880	0.773	0.564
	0.5	0.850	0.830	0.868	0.810	0.910	0.620	0.800	0.750	0.773	0.564
DT	0.1	0.826	0.717	0.990	0.831	0.972	0.707	0.720	0.876	0.790	0.747
	0.3	0.891	0.828	0.946	0.883	0.972	0.701	0.771	0.746	0.758	0.747
	0.5	0.909	0.899	0.891	0.895	0.972	0.699	0.803	0.692	0.744	0.747

GLM: Generalized Linear Model; ConvLSTM: Convolutional Long Short-Term Memory (2D); MLP: Multi-Layer Perceptron; LSTM: Long Short-Term Memory (2 time sequence); SVM: Support Vector Machine; XGB: eXtreme Gradient Boosting.

Table S17. Spatio-temporal model comparison metrics for yearly dengue cases in Bangladesh (DIC, WAIC, meanCPO, and Marginal Log-Likelihood)

Covariates	Model	DIC	WAIC	meanCPO	MargLogLik
All variables used	IID_IID	4985.702	4994.885	5.528	-2623.170
All variables used	IID_RW1	4985.461	4994.626	5.528	-2625.894
All variables used	IID_AR1	4987.212	4996.510	5.530	-2624.461
All variables used	BYM2_IID	4984.975	4994.108	5.527	-2579.337
All variables used	BYM2_RW1	4984.540	4993.642	5.527	-2582.092
All variables used	BYM2_AR1	4984.909	4994.068	5.527	-2580.838
All variables used	IID_RW2	4985.554	4994.798	5.528	-2624.652
All variables used	BYM2_RW2	4984.298	4993.463	5.526	-2580.830
Top 10 selected variable used	IID_IID	4986.532	4994.249	5.533	-2608.668
Top 10 selected variable used	IID_RW1	4986.177	4993.892	5.532	-2611.463
Top 10 selected variable used	IID_AR1	4988.684	4996.496	5.534	-2609.289
Top 10 selected variable used	BYM2_IID	4983.320	4991.184	5.526	-2563.538
Top 10 selected variable used	BYM2_RW1	4983.430	4991.155	5.526	-2566.322
Top 10 selected variable used	BYM2_AR1	4983.828	4991.788	5.527	-2564.706
Top 10 selected variable used	IID_RW2	4986.541	4994.437	5.533	-2610.071
**Top 10 selected variable used**	**BYM2_RW2**	**4983.317**	**4991.185**	**5.526**	**-2564.923**
Top 10 selected variable used	BYM2_RW2_Lag1	4950.211	4961.448	5.962	-2542.058
**Top 10 selected variable used**	BYM2_RW2_Lag12	4550.611	4556.168	6.478	-2347.707
Top 10 selected variable used	**BYM2_RW2_Lag123**	**3671.055**	**3675.390**	**6.557**	**-1911.331**

IID_IID: Independent and identically distributed spatial and temporal effects; IID_RW1: IID spatial effects with RW1 (first-order random walk) temporal effects; IID_AR1: IID spatial effects with AR1 (first-order autoregressive) temporal effects; BYM2_IID: BYM2 (Besag–York–Mollié 2) spatial + unstructured effects with IID temporal effects; BYM2_RW1: BYM2 spatial effects with RW1 temporal effects; BYM2_AR1: BYM2 spatial effects with AR1 temporal effects; IID_RW2: IID spatial effects with RW2 (second-order random walk) temporal effects; BYM2_RW2: BYM2 spatial effects with RW2 temporal effects; BYM2_RW2_Lag1: BYM2 spatial effects with RW2 temporal effects and 1-month lag; BYM2_RW2_Lag12: BYM2 spatial effects with RW2 temporal effects and 12-month lag; BYM2_RW2_Lag123: BYM2 spatial effects with RW2 temporal effects and combined 1–3 month lags; Covariates = variables included in the model; Model = model specification; DIC = Deviance Information Criterion; WAIC = Watanabe–Akaike Information Criterion; meanCPO = mean Conditional Predictive Ordinate, measuring model predictive performance; MargLogLik = marginal log-likelihood, indicating model fit. Lower DIC and WAIC values indicate better model fit, while higher meanCPO and marginal log-likelihood indicate better predictive performance.

Table S18. Spatio-temporal model comparison metrics for monthly dengue cases using all variables and top 10 selected variables.

Covariates	Model	DIC	WAIC	meanCPO	MargLogLik
All variables used	IID_IID	14690.741	14702.201	3.855	-7596.058
All variables used	IID_RW1	14689.013	14700.480	3.854	-7573.269
All variables used	IID_AR1	14689.103	14700.514	3.854	-7577.012
All variables used	IID_RW2	14689.544	14701.280	3.854	-7576.609
All variables used	BYM2_IID	14690.484	14702.124	3.855	-7546.895
All variables used	BYM2_RW1	14688.706	14700.350	3.854	-7524.094
All variables used	BYM2_AR1	14688.833	14700.368	3.854	-7527.861
All variables used	BYM2_RW2	14689.293	14701.181	3.854	-7527.445
Top 10 selected variable used	IID_IID	14687.851	14698.167	3.854	-7588.125
**Top 10 selected variable used**	**IID_RW1**	**14686.041**	**14696.242**	**3.853**	**-7565.340**
Top 10 selected variable used	IID_AR1	14686.564	14697.365	3.853	-7569.092
Top 10 selected variable used	IID_RW2	14686.586	14697.093	3.853	-7568.681
Top 10 selected variable used	BYM2_IID	14687.827	14698.824	3.854	-7538.953
Top 10 selected variable used	BYM2_RW1	14686.135	14697.111	3.853	-7516.170
Top 10 selected variable used	BYM2_AR1	14686.557	14697.717	3.854	-7519.886
**Top 10 selected variable used**	BYM2_RW2	14686.682	14697.922	3.853	-7519.471
Top 10 selected variable used	IID_RW1_Lag1	17680.871	17691.398	4.039	-9093.603
**Top 10 selected variable used**	IID_RW1_Lag12	17561.980	17573.858	4.542	-9047.197
**Top 10 selected variable used**	**IID_RW1_Lag123**	**17434.506**	**17447.737**	**4.901**	**-8996.363**

IID_IID: Independent and identically distributed spatial and temporal effects; IID_RW1: IID spatial effects with RW1 (first-order random walk) temporal effects; IID_AR1: IID spatial effects with AR1 (first-order autoregressive) temporal effects; BYM2_IID: BYM2 (Besag–York–Mollié 2) spatial + unstructured effects with IID temporal effects; BYM2_RW1: BYM2 spatial effects with RW1 temporal effects; BYM2_AR1: BYM2 spatial effects with AR1 temporal effects; IID_RW2: IID spatial effects with RW2 (second-order random walk) temporal effects; BYM2_RW2: BYM2 spatial effects with RW2 temporal effects; BYM2_RW2_Lag1: BYM2 spatial effects with RW2 temporal effects and 1-month lag; BYM2_RW2_Lag12: BYM2 spatial effects with RW2 temporal effects and 12-month lag; BYM2_RW2_Lag123: BYM2 spatial effects with RW2 temporal effects and combined 1–3 month lags; Covariates = variables included in the model; Model = model specification; DIC = Deviance Information Criterion; WAIC = Watanabe–Akaike Information Criterion; meanCPO = mean Conditional Predictive Ordinate, measuring model predictive performance; MargLogLik = marginal log-likelihood, indicating model fit. Lower DIC and WAIC values indicate better model fit, while higher meanCPO and marginal log-likelihood indicate better predictive performance; Covariates = variables included in the model; Model = model specification; DIC = Deviance Information Criterion; WAIC = Watanabe–Akaike Information Criterion; meanCPO = mean Conditional Predictive Ordinate, measuring model predictive performance; MargLogLik = marginal log-likelihood, indicating model fit. Lower DIC and WAIC values indicate better model fit, while higher meanCPO and marginal log-likelihood indicate better predictive performance.

Table S19. Category wise summed contribution on yearly dengue case prediction.

Category	SHAP_Sum	Contribution_%

Climate & Environmental	0.405	66.354
Socio-Demographic & Economic	0.099	16.268
Healthcare System	0.044	7.171
Land Use & Land Cover	0.062	10.207

Table S20. Category wise summed contribution on monthly dengue case prediction.

Category	Contribution (%)
Climate & Environmental Factors	54.435
Socio-Demographic & Economic Indicators	17.209
Healthcare System Capacity & Resources	22.794
Land Use and Land Cover Indicators	5.562

Table S21. Top 10 feature to predict yearly dengue cases in Bangladesh

Feature	Mean_SHAP
X12	0.145
X11	0.137
X111	0.077
X120	0.062
X14	0.049
X13	0.044
X116	0.044
X112	0.022
X15	0.017
X16	0.013

X12: Minimum yearly temperature at 2 meters (C); X11: Yearly average temperature at 2 meters (C); X111: Yearly population growth(%); X120: Yearly arable land; X14: Yearly relative humidity at 2 meters (%); X13: Maximum yearly temperature at 2 meters (C); X116: Yearly domestic general government health expenditure (%); X112: Yearly average household size; X15: Yearly rainfall corrected (mm/day); X16: Yearly surface pressure (kPa);

Table S22. Senario analysis of the top 10 features on yearly dengue case prediction

Feature	X12	X11	X111	X120	X14	X13	X116	X112	X15	X16

Chang e_%	Predicted Probability	Predicted Probability	Predicted Probability	Predicted Probability	Predicted Probability	Predicted Probability	Predicted Probability	Predicted Probability	Predicted Probability	Predicted Probability
-50	0.017	0.011	0.005	0.011	0.004	0.006	0.005	0.008	0.004	0.006
-40	0.014	0.010	0.005	0.010	0.005	0.006	0.006	0.008	0.004	0.006
-30	0.011	0.008	0.006	0.009	0.005	0.006	0.006	0.007	0.004	0.006
-20	0.009	0.007	0.006	0.008	0.006	0.006	0.006	0.007	0.005	0.006
-10	0.008	0.007	0.006	0.007	0.006	0.006	0.006	0.007	0.006	0.006
0	0.006	0.006	0.006	0.006	0.006	0.006	0.006	0.006	0.006	0.006
10	0.005	0.006	0.006	0.006	0.006	0.006	0.006	0.006	0.007	0.006
20	0.004	0.005	0.006	0.005	0.007	0.006	0.006	0.005	0.007	0.006
30	0.003	0.005	0.006	0.004	0.007	0.006	0.005	0.005	0.008	0.006
40	0.003	0.005	0.005	0.004	0.007	0.006	0.005	0.005	0.009	0.006
50	0.002	0.005	0.005	0.003	0.007	0.006	0.004	0.004	0.010	0.006

X12: Minimum yearly temperature at 2 meters (C); X11: Yearly average temperature at 2 meters (C); X111: Yearly population growth(%); X120: Yearly arable land; X14: Yearly relative humidity at 2 meters (%); X13: Maximum yearly temperature at 2 meters (C); X116: Yearly domestic general government health expenditure (%); X112: Yearly average household size; X15: Yearly rainfall corrected (mm/day); X16: Yearly surface pressure (kPa);

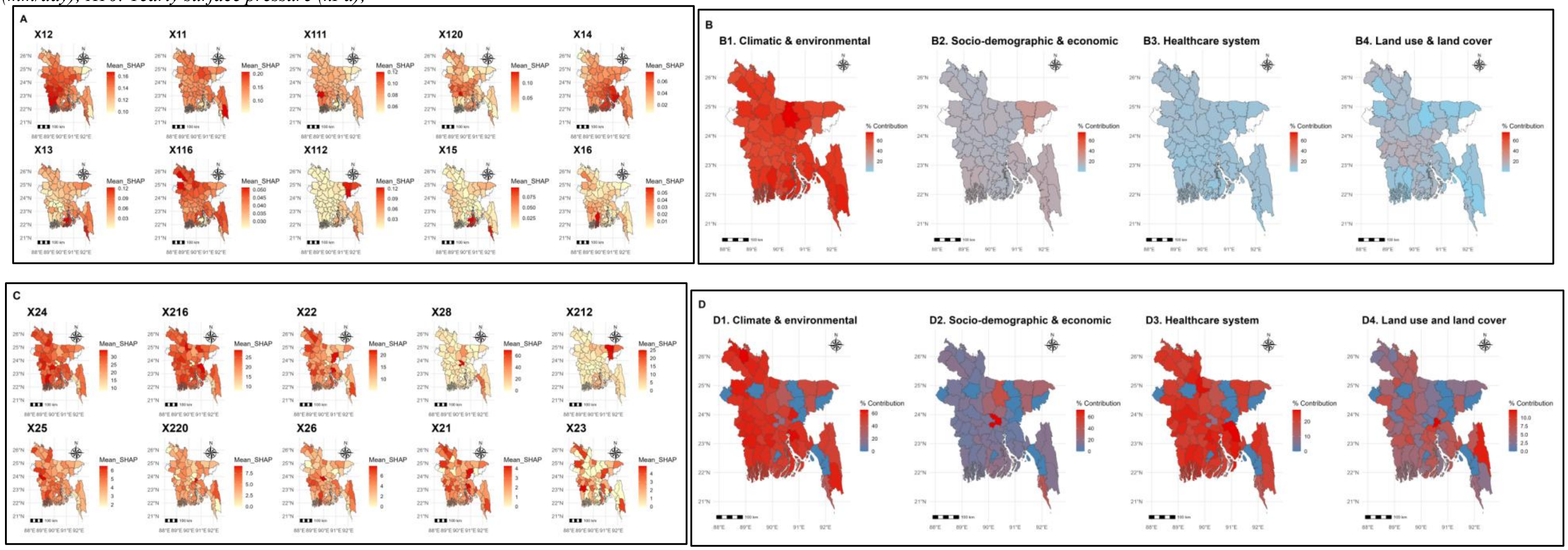

Figure S8. A) District-wise SHAP analysis identifying the top 10 contributing features for yearly dengue case prediction. B) District-wise aggregated SHAP contributions by category for yearly dengue case prediction. C) District-wise SHAP analysis identifying the top 10 contributing features for monthly dengue case prediction. D) District-wise aggregated SHAP contributions by category for monthly dengue case prediction; X12: Minimum yearly temperature at 2 meters (C); X11: Yearly average temperature at 2 meters (C); X111: Yearly population growth(%); X120: Yearly arable land; X14: Yearly relative humidity at 2 meters (%); X13: Maximum yearly temperature at 2 meters (C); X116: Yearly domestic general government health expenditure (%); X112: Yearly average household size; X15: Yearly rainfall corrected (mm/day); X16: Yearly surface pressure (kPa); X24: Monthly relative humidity at 3 meters (%); X216: Monthly domestic general government health expenditure (%); X22: Minimum monthly temperature at 3 meters (C); X28: Total monthly population (each district); X212: Average monthly household size; X25: Monthly rainfall corrected (mm/day); X220: Monthly arable land; X26: Monthly surface pressure (kPa); X21: Monthly average temperature at 2 meters (C); X23: Maximum

Table S23. District wise SHAP analysis for selection of top 10 contributing feature on yearly dengue case prediction

District_yearly SHAP (A)	X12	X11	X111	X120	X14	X13	X116	X112	X15	X16
Bagerhat	0.143	0.144	0.082	0.013	0.038	0.029	0.044	0.010	0.004	0.058
Bandarban	0.103	0.207	0.077	0.007	0.048	0.071	0.045	0.034	0.015	0.003
Barguna	0.145	0.130	0.078	0.074	0.069	0.055	0.046	0.011	0.003	0.012
Barishal	0.143	0.138	0.082	0.044	0.065	0.066	0.047	0.011	0.020	0.014
Bhola	0.153	0.079	0.053	0.086	0.070	0.115	0.029	0.042	0.092	0.006
Bogura	0.154	0.163	0.075	0.047	0.049	0.047	0.043	0.011	0.006	0.022
Brahmanbaria	0.145	0.119	0.080	0.053	0.053	0.032	0.052	0.029	0.006	0.001
Chandpur	0.144	0.119	0.074	0.065	0.069	0.054	0.046	0.036	0.002	0.010
Chapai Nawabganj	0.171	0.065	0.095	0.106	0.018	0.066	0.047	0.011	0.050	0.011
Chattogram	0.139	0.125	0.078	0.005	0.051	0.081	0.049	0.040	0.019	0.024
Chuadanga	0.166	0.102	0.087	0.120	0.028	0.039	0.043	0.010	0.034	0.015
Cox's Bazar	0.152	0.056	0.062	0.062	0.061	0.120	0.035	0.091	0.096	0.015
Cumilla	0.139	0.160	0.082	0.028	0.073	0.058	0.044	0.038	0.002	0.017
Dhaka	0.154	0.137	0.074	0.086	0.057	0.038	0.042	0.010	0.008	0.015
Dinajpur	0.120	0.148	0.079	0.018	0.027	0.037	0.051	0.008	0.009	0.036
Faridpur	0.166	0.143	0.085	0.087	0.052	0.001	0.040	0.010	0.017	0.024

Feni	0.120	0.155	0.075	0.060	0.076	0.074	0.042	0.029	0.008	0.007
Gaibandha	0.145	0.148	0.073	0.063	0.040	0.046	0.047	0.007	0.001	0.012
Gazipur	0.156	0.140	0.075	0.078	0.058	0.039	0.042	0.010	0.008	0.015
Gopalganj	0.161	0.158	0.088	0.094	0.061	0.001	0.037	0.010	0.003	0.018
Habiganj	0.145	0.099	0.061	0.041	0.050	0.030	0.041	0.123	0.017	0.008
Jamalpur	0.143	0.143	0.079	0.056	0.048	0.037	0.051	0.011	0.006	0.003
Jashore	0.166	0.158	0.120	0.085	0.053	0.060	0.037	0.011	0.020	0.035
Jhalokathi	0.145	0.131	0.085	0.076	0.059	0.027	0.049	0.007	0.005	0.007
Jhenaidah	0.165	0.142	0.084	0.090	0.052	0.001	0.040	0.010	0.017	0.024
Joypurhat	0.144	0.141	0.069	0.095	0.048	0.040	0.043	0.010	0.005	0.017
Khagrachhari	0.134	0.168	0.083	0.030	0.060	0.042	0.047	0.032	0.009	0.003
Khulna	0.167	0.132	0.098	0.025	0.044	0.017	0.043	0.012	0.021	0.021
Kishorganj	0.160	0.171	0.075	0.064	0.050	0.034	0.039	0.010	0.024	0.017
Kurigram	0.136	0.155	0.072	0.054	0.033	0.049	0.049	0.007	0.009	0.003
Kushtia	0.155	0.126	0.075	0.082	0.044	0.022	0.045	0.009	0.017	0.008
Lakshmipur	0.136	0.129	0.077	0.059	0.071	0.060	0.047	0.030	0.005	0.001
Lalmonirhat	0.132	0.146	0.069	0.075	0.034	0.047	0.048	0.007	0.008	0.003
Madaripur	0.159	0.154	0.087	0.102	0.061	0.001	0.038	0.010	0.003	0.017
Magura	0.160	0.132	0.081	0.112	0.051	0.000	0.041	0.010	0.015	0.021
Manikganj	0.154	0.138	0.075	0.089	0.056	0.032	0.042	0.010	0.009	0.014
Meherpur	0.145	0.188	0.077	0.044	0.049	0.040	0.043	0.010	0.020	0.003
Moulvibazar	0.138	0.057	0.062	0.078	0.020	0.002	0.042	0.096	0.017	0.007
Munshiganj	0.145	0.140	0.073	0.092	0.068	0.054	0.041	0.010	0.006	0.014
Mymensingh	0.147	0.189	0.076	0.001	0.042	0.052	0.046	0.020	0.024	0.008
Naogaon	0.139	0.138	0.079	0.020	0.029	0.025	0.051	0.009	0.024	0.026
Narail	0.165	0.140	0.103	0.137	0.052	0.054	0.037	0.010	0.017	0.027
Narayanganj	0.144	0.138	0.073	0.096	0.067	0.053	0.041	0.010	0.006	0.014
Narsingdi	0.152	0.135	0.073	0.093	0.057	0.037	0.042	0.010	0.008	0.014
Natore	0.155	0.117	0.075	0.075	0.028	0.009	0.047	0.010	0.028	0.008
Netrokona	0.140	0.174	0.075	0.040	0.046	0.044	0.048	0.017	0.023	0.007
Nilphamari	0.127	0.148	0.069	0.070	0.027	0.052	0.047	0.007	0.009	0.007

Noakhali	0.127	0.164	0.078	0.032	0.076	0.078	0.043	0.033	0.008	0.007
Pabna	0.160	0.134	0.076	0.064	0.045	0.024	0.045	0.011	0.019	0.009
Panchagarh	0.109	0.111	0.061	0.073	0.011	0.079	0.046	0.007	0.017	0.002
Patuakhali	0.152	0.077	0.049	0.064	0.071	0.123	0.026	0.047	0.097	0.007
Pirojpur	0.136	0.126	0.085	0.057	0.049	0.027	0.049	0.003	0.005	0.034
Rajbari	0.161	0.133	0.081	0.110	0.051	0.000	0.041	0.010	0.015	0.022
Rajshahi	0.158	0.121	0.078	0.063	0.028	0.010	0.048	0.010	0.030	0.008
Rangamati	0.131	0.168	0.077	0.035	0.038	0.085	0.048	0.044	0.009	0.008
Rangpur	0.146	0.150	0.073	0.059	0.040	0.046	0.047	0.007	0.001	0.012
Satkhira	0.167	0.131	0.096	0.033	0.044	0.017	0.044	0.012	0.020	0.020
Shariatpur	0.139	0.150	0.076	0.070	0.067	0.060	0.043	0.010	0.004	0.003
Sherpur	0.140	0.138	0.076	0.070	0.049	0.036	0.050	0.010	0.006	0.003
Sirajganj	0.154	0.138	0.075	0.055	0.042	0.030	0.047	0.011	0.014	0.002
Sunamganj	0.117	0.134	0.055	0.012	0.042	0.068	0.040	0.120	0.038	0.002
Sylhet	0.098	0.152	0.056	0.016	0.044	0.078	0.038	0.108	0.037	0.000
Tangail	0.154	0.161	0.081	0.027	0.045	0.033	0.046	0.010	0.002	0.005
Thakurgaon	0.136	0.113	0.069	0.068	0.007	0.044	0.048	0.007	0.006	0.006

X12: Minimum yearly temperature at 2 meters (C); X11: Yearly average temperature at 2 meters (C); X111: Yearly population growth(%); X120: Yearly arable land; X14: Yearly relative humidity at 2 meters (%); X13: Maximum yearly temperature at 2 meters (C); X116: Yearly domestic general government health expenditure (%); X112: Yearly average household size; X15: Yearly rainfall corrected (mm/day); X16: Yearly surface pressure (kPa);

Table S24. Districtwise total aggregated shap contribution of different categories on yearly dengue case prediction

District (Data1)	Climatic and environmental	Socio-demographic and economic	Healthcare	Land use
Bagerhat	73.633	16.308	7.741	2.318
Bandarban	73.203	18.242	7.338	1.217
Barguna	66.471	14.269	7.357	11.903
Barishal	70.710	14.857	7.421	7.011
Bhola	70.903	13.173	4.032	11.893
Bogura	71.437	13.965	6.907	7.691
Brahmanbaria	62.510	19.135	9.093	9.261

Chandpur	64.310	17.795	7.454	10.441
Chapai Nawabganj	59.565	16.581	7.317	16.537
Chattogram	71.960	19.355	7.945	0.740
Chuadanga	59.513	15.161	6.731	18.595
Cox's Bazar	66.689	20.332	4.685	8.294
Cumilla	70.016	18.650	6.897	4.437
Dhaka	65.770	13.522	6.802	13.905
Dinajpur	70.614	16.391	9.624	3.371
Faridpur	64.468	15.124	6.462	13.945
Feni	68.118	16.031	6.486	9.365
Gaibandha	67.393	13.650	8.041	10.916
Gazipur	66.957	13.666	6.764	12.612
Gopalganj	63.767	15.447	5.905	14.881
Habiganj	56.708	29.951	6.740	6.601
Jamalpur	65.894	15.573	8.816	9.716
Jashore	66.023	17.565	4.979	11.433
Jhalokathi	63.338	15.527	8.219	12.915
Jhenaidah	64.144	15.056	6.464	14.337
Joypurhat	64.513	12.983	6.940	15.564
Khagrachhari	68.442	18.935	7.658	4.964
Khulna	69.363	18.947	7.394	4.296
Kishorganj	70.840	13.174	6.015	9.971
Kurigram	67.891	13.963	8.652	9.495
Kushtia	63.739	14.426	7.772	14.064
Lakshmipur	65.315	17.455	7.666	9.564
Lalmonirhat	65.067	13.363	8.402	13.167
Madaripur	62.600	15.311	5.964	16.124
Magura	60.959	14.539	6.500	18.003
Manikganj	65.027	13.698	6.855	14.420
Meherpur	71.931	13.967	6.942	7.160
Moulvibazar	46.476	30.381	8.167	14.976

Munshiganj	66.357	12.939	6.419	14.286
Mymensingh	76.290	15.971	7.641	0.098
Naogaon	70.398	16.409	9.497	3.697
Narail	61.275	15.268	5.038	18.419
Narayanganj	65.771	12.833	6.401	14.995
Narsingdi	64.805	13.382	6.812	15.001
Natore	62.428	15.423	8.520	13.629
Netrokona	70.763	14.972	7.751	6.514
Nilphamari	65.783	13.376	8.419	12.422
Noakhali	71.336	17.161	6.604	4.899
Pabna	66.613	14.846	7.606	10.936
Panchagarh	63.878	13.077	8.871	14.175
Patuakhali	73.871	13.531	3.597	9.001
Pirojpur	66.056	15.450	8.607	9.887
Rajbari	61.257	14.581	6.496	17.667
Rajshahi	64.159	15.875	8.625	11.341
Rangamati	68.170	18.864	7.456	5.510
Rangpur	68.027	13.746	8.059	10.167
Satkhira	68.370	18.550	7.447	5.634
Shariatpur	68.022	13.811	6.922	11.245
Sherpur	64.234	14.908	8.670	12.187
Sirajganj	66.880	15.131	8.231	9.758
Sunamganj	63.845	27.909	6.282	1.964
Sylhet	65.254	26.122	6.056	2.568
Tangail	70.922	16.159	8.113	4.806
Thakurgaon	61.968	15.055	9.535	13.442

Table S25. Posterior estimates of fixed effects for the BYM2_RW2_Lag123 model using standardized yearly predictors.

Variable	mean	sd	0.025quant	0.5quant	0.975quant	mode	kld	RR	RR_lower	RR_upper	P_gt0	BayesSig	Pval
(Intercept)	4.719	0.143	4.437	4.719	5.001	4.719	0.000	112.057	84.554	148.521	0.000	1.000	0.000

X12	-0.683	0.162	-1.001	-0.683	-0.366	-0.683	0.000	0.505	0.368	0.694	1.000	1.000	0.000
X11	0.456	0.250	-0.039	0.457	0.944	0.457	0.000	1.577	0.962	2.569	0.034	0.966	0.069
X120	-0.018	0.126	-0.266	-0.018	0.230	-0.018	0.000	0.982	0.766	1.258	0.556	0.556	0.888
X14	0.580	0.254	0.079	0.582	1.075	0.582	0.000	1.787	1.082	2.929	0.011	0.989	0.022
X13	0.196	0.128	-0.056	0.196	0.448	0.196	0.000	1.217	0.946	1.564	0.063	0.937	0.126
X112	-0.505	0.192	-0.880	-0.505	-0.128	-0.505	0.000	0.604	0.415	0.880	0.996	0.996	0.008
X15	0.265	0.173	-0.073	0.264	0.605	0.264	0.000	1.303	0.930	1.830	0.063	0.937	0.125
X16	-0.009	0.062	-0.131	-0.009	0.113	-0.009	0.000	0.991	0.877	1.120	0.557	0.557	0.885
lag1	0.000	0.000	0.000	0.000	0.000	0.000	0.000	1.000	1.000	1.000	0.889	0.889	0.222
lag2	0.000	0.000	0.000	0.000	0.000	0.000	0.000	1.000	1.000	1.000	0.612	0.612	0.775
lag3	0.000	0.000	0.000	0.000	0.000	0.000	0.000	1.000	1.000	1.000	0.044	0.956	0.087

mean = *posterior mean;* sd = *posterior standard deviation;* 0.025quant = *2.5th percentile of the posterior distribution;* 0.5quant = *median of the posterior distribution;* 0.975quant = *97.5th percentile of the posterior distribution;* mode = *posterior mode (highest density);* kld = *Kullback–Leibler divergence, quantifying the difference between the posterior and prior distributions.*

; X12: Minimum yearly temperature at 2 meters (C); X11: Yearly average temperature at 2 meters (C); X120: Yearly arable land; X14: Yearly relative humidity at 2 meters (%); X13: Maximum yearly temperature at 2 meters (C); X112: Yearly average household size; X15: Yearly rainfall corrected (mm/day); X16: Yearly surface pressure (kPa); lag1: 1-month lag of dengue cases; lag2: 2-month lag of dengue cases; lag3: 3-month lag of dengue cases;

Table S26. Posterior estimates of random effects for the BYM2_RW2_Lag123 model using standardized yearly predictors.

ID	mean	sd	0.025quant	0.5quant	0.975quant	mode	kld	District_upper
1	-1.083	0.432	-1.931	-1.083	-0.236	-1.083	0.000	BAGERHAT
2	0.247	0.508	-0.753	0.248	1.242	0.248	0.000	BANDARBAN
3	0.715	0.390	-0.049	0.715	1.481	0.715	0.000	BARGUNA
4	0.909	0.407	0.113	0.909	1.709	0.909	0.000	BARISAL
5	-0.483	0.492	-1.450	-0.483	0.482	-0.483	0.000	BHOLA
6	-0.171	0.397	-0.950	-0.171	0.608	-0.171	0.000	BOGRA
7	0.124	0.427	-0.714	0.124	0.963	0.124	0.000	BRAHAMANBARIA
8	1.343	0.430	0.502	1.342	2.188	1.342	0.000	CHANDPUR
9	1.116	0.487	0.158	1.116	2.070	1.116	0.000	NAWABGANJ
10	2.078	0.450	1.197	2.077	2.964	2.077	0.000	CHITTAGONG
11	-1.425	0.441	-2.291	-1.425	-0.560	-1.425	0.000	CHUADANGA
12	1.256	0.540	0.198	1.255	2.315	1.255	0.000	COX'S BAZAR
13	0.921	0.440	0.060	0.920	1.786	0.920	0.000	COMILLA
14	4.738	0.398	3.965	4.735	5.527	4.735	0.000	DHAKA
15	-0.200	0.442	-1.068	-0.200	0.668	-0.200	0.000	DINAJPUR
16	0.787	0.364	0.074	0.787	1.500	0.787	0.000	FARIDPUR
17	-0.038	0.496	-1.009	-0.038	0.936	-0.038	0.000	FENI
18	-1.280	0.414	-2.093	-1.280	-0.468	-1.280	0.000	GAIBANDHA
19	1.936	0.356	1.239	1.936	2.637	1.936	0.000	GAZIPUR
20	-0.716	0.410	-1.520	-0.716	0.087	-0.716	0.000	GOPALGANJ
21	-0.469	0.595	-1.640	-0.468	0.695	-0.468	0.000	HABIGANJ
22	-0.173	0.352	-0.864	-0.173	0.517	-0.173	0.000	JAMALPUR
23	0.882	0.383	0.131	0.881	1.634	0.881	0.000	JESSORE
24	-0.981	0.429	-1.823	-0.981	-0.140	-0.981	0.000	JHALOKATI
25	-0.262	0.387	-1.022	-0.262	0.497	-0.262	0.000	JHENAIDAH
26	-2.226	0.457	-3.122	-2.226	-1.331	-2.226	0.000	JOYPURHAT

27	-0.708	0.472	-1.634	-0.708	0.219	-0.708	0.000	KHAGRACHHARI
28	0.854	0.403	0.065	0.854	1.646	0.854	0.000	KHULNA
29	0.374	0.370	-0.352	0.374	1.099	0.374	0.000	KISHOREGANJ
30	-1.124	0.443	-1.992	-1.123	-0.256	-1.123	0.000	KURIGRAM
31	0.404	0.373	-0.328	0.404	1.137	0.404	0.000	KUSHTIA
32	0.309	0.466	-0.603	0.308	1.225	0.308	0.000	LAKSHMIPUR
33	-2.096	0.465	-3.008	-2.096	-1.184	-2.096	0.000	LALMONIRHAT
34	0.179	0.387	-0.580	0.179	0.938	0.179	0.000	MADARIPUR
35	-0.154	0.395	-0.928	-0.154	0.621	-0.154	0.000	MAGURA
36	0.765	0.368	0.044	0.765	1.487	0.765	0.000	MANIKGANJ
37	-1.140	0.407	-1.939	-1.140	-0.342	-1.140	0.000	MEHERPUR
38	-0.082	0.678	-1.415	-0.081	1.245	-0.081	0.000	MAULVIBAZAR
39	-0.492	0.427	-1.329	-0.492	0.346	-0.492	0.000	MUNSHIGANJ
40	1.023	0.399	0.241	1.023	1.807	1.023	0.000	MYMENSINGH
41	-0.406	0.458	-1.305	-0.405	0.492	-0.405	0.000	NAOGAON
42	0.174	0.388	-0.587	0.174	0.934	0.174	0.000	NARAIL
43	0.270	0.415	-0.545	0.270	1.086	0.270	0.000	NARAYANGANJ
44	0.322	0.382	-0.428	0.322	1.072	0.322	0.000	NARSINGDI
45	-0.472	0.431	-1.319	-0.472	0.374	-0.472	0.000	NATORE
46	-0.795	0.392	-1.564	-0.795	-0.025	-0.795	0.000	NETRAKONA
47	-0.568	0.462	-1.476	-0.567	0.337	-0.567	0.000	NILPHAMARI
48	-0.696	0.478	-1.633	-0.696	0.243	-0.696	0.000	NOAKHALI
49	0.560	0.378	-0.182	0.560	1.302	0.560	0.000	PABNA
50	-1.509	0.620	-2.732	-1.507	-0.301	-1.507	0.000	PANCHAGARH
51	0.208	0.484	-0.741	0.207	1.157	0.207	0.000	PATUAKHALI
52	0.302	0.391	-0.464	0.302	1.068	0.302	0.000	PIROJPUR
53	-0.531	0.400	-1.315	-0.530	0.253	-0.530	0.000	RAJBARI
54	0.888	0.414	0.074	0.888	1.700	0.888	0.000	RAJSHAHI
55	-0.824	0.576	-1.957	-0.823	0.302	-0.823	0.000	RANGAMATI
56	-0.866	0.392	-1.636	-0.866	-0.097	-0.866	0.000	RANGPUR
57	-0.795	0.426	-1.631	-0.795	0.042	-0.795	0.000	SATKHIRA

58	-0.279	0.436	-1.133	-0.280	0.576	-0.280	0.000	SHARIATPUR
59	-0.536	0.376	-1.274	-0.536	0.202	-0.536	0.000	SHERPUR
60	-0.216	0.390	-0.983	-0.216	0.549	-0.216	0.000	SIRAJGANJ
61	-1.489	0.664	-2.799	-1.487	-0.193	-1.487	0.000	SUNAMGANJ
62	-0.080	0.605	-1.272	-0.078	1.102	-0.078	0.000	SYLHET
63	0.737	0.374	0.004	0.737	1.472	0.737	0.000	TANGAIL
64	-1.352	0.572	-2.480	-1.350	-0.234	-1.350	0.000	THAKURGAON

Table S27. Posterior estimates of temporal effects for the BYM2_RW2_Lag123 model using standardized yearly predictors.

ID	mean	sd	0.025quant	0.5quant	0.975quant	mode	kld	Year
4	-2.457	0.104	-2.660	-2.457	-2.253	-2.457	0.000	2020
5	-1.229	0.052	-1.331	-1.229	-1.127	-1.229	0.000	2021
6	0.000	0.007	-0.014	0.000	0.013	0.000	0.000	2022
7	1.228	0.052	1.126	1.228	1.330	1.228	0.000	2023
8	2.457	0.104	2.254	2.457	2.660	2.457	0.000	2024

mean = *posterior mean;* sd = *posterior standard deviation;* 0.025quant = *2.5th percentile of the posterior distribution;* 0.5quant = *median of the posterior distribution;* 0.975quant = *97.5th percentile of the posterior distribution;* mode = *posterior mode (highest density);* kld = *Kullback–Leibler divergence, quantifying the difference between the posterior and prior distribution.*

Table S28. Top 10 contribution of the factor on prediction of monthly dengue cases

Feature	Contribution (%)
**X24**	28.002
**X216**	23.811
**X22**	15.545
**X28**	10.233
**X212**	4.865
**X25**	4.393
**X220**	4.228
**X26**	3.766
**X21**	2.605
**X23**	2.551

X24: Monthly relative humidity at 3 meters (%); X216: Monthly domestic general government health expenditure (%); X22: Minimum monthly temperature at 3 meters (C); X28: Total monthly population (each district); X212: Average monthly household size; X25: Monthly rainfall corrected (mm/day); X220: Monthly arable land; X26: Monthly surface pressure (kPa); X21: Monthly average temperature at 2 meters (C); X23: Maximum monthly temperature at 3 meters (C);

Table S29. Top 10 contribution of the factor on prediction of district wise monthly dengue cases

Districts	X21	X212	X216	X22	X220	X23	X24	X25	X26	X28
**Bagerhat**	2.352	2.308	26.278	13.444	3.918	1.446	30.148	4.649	7.203	8.255
**Bandarban**	3.759	2.833	21.445	20.066	0.000	4.381	27.020	3.601	4.778	9.843
**Barguna**	2.097	8.553	24.299	14.656	3.318	1.627	26.945	4.409	4.293	9.803
**Barishal**	1.996	9.499	26.973	12.402	4.367	0.000	31.588	5.324	2.793	0.000
**Bhola**	3.539	7.640	22.028	19.807	1.855	4.026	27.675	4.047	2.300	7.082
**Bogura**	2.021	2.533	19.953	15.065	4.764	0.000	27.371	3.894	1.790	18.000
**Brahmanbaria**	2.430	3.024	28.617	14.709	1.880	2.023	32.632	4.918	3.029	6.738
**Chandpur**	4.253	3.330	25.928	22.057	2.078	4.335	28.838	3.720	3.202	2.259
**Chapai Nawabganj**	2.035	3.489	26.792	15.732	0.000	1.968	31.973	5.398	2.330	8.692
**Chattogram**	1.796	5.610	12.096	6.820	2.987	0.000	13.168	2.490	1.740	51.422
**Chuadanga**	3.750	1.543	23.409	19.912	4.414	3.839	25.338	3.482	6.367	7.945
**Cox's Bazar**	1.971	4.123	16.436	11.203	2.835	0.000	19.594	3.028	0.000	37.374
**Cumilla**	2.257	4.008	28.764	14.185	5.669	1.823	30.962	4.238	5.693	2.402
**Dhaka**	1.205	1.499	7.929	5.286	0.000	0.910	8.417	1.783	1.534	69.679
**Dinajpur**	2.623	2.189	24.405	16.402	3.668	0.000	29.813	4.425	2.474	10.218
**Faridpur**	2.095	0.000	25.877	13.498	2.966	1.897	34.196	5.586	3.401	7.255
**Feni**	3.712	2.864	23.201	18.978	6.320	4.152	25.493	3.728	2.906	8.645
**Gaibandha**	0.000	2.703	27.382	16.291	4.109	0.000	33.655	5.821	2.151	2.142
**Gazipur**	2.235	2.187	22.488	11.910	0.000	2.136	28.742	4.566	2.802	18.527
**Gopalganj**	3.549	1.467	22.173	19.665	2.183	3.906	28.566	4.729	3.331	10.431
**Habiganj**	0.000	24.652	23.056	11.473	4.643	1.680	23.400	4.099	1.904	3.263
**Jamalpur**	2.310	9.548	28.690	13.110	3.228	2.006	30.843	4.479	3.553	2.234
**Jashore**	3.908	2.278	23.083	20.724	5.286	4.921	27.859	3.979	4.353	3.608

**Jhalokathi**	1.912	7.825	22.068	13.874	7.880	0.000	24.245	3.556	6.820	8.601
**Jhenaidah**	2.008	1.724	26.686	13.225	2.248	2.180	33.710	5.290	6.974	5.954
**Joypurhat**	3.615	2.366	20.667	19.459	6.239	4.217	26.273	3.878	4.816	8.471
**Khagrachhari**	1.897	2.528	25.503	14.132	5.451	0.000	27.696	4.545	4.728	11.723
**Khulna**	2.460	2.438	27.157	13.747	4.072	0.000	34.448	6.105	3.097	2.511
**Kishorganj**	4.228	0.000	20.479	21.940	4.905	4.393	26.297	3.537	0.000	7.961
**Kurigram**	2.462	0.000	25.313	16.832	4.747	0.000	31.660	5.806	2.634	4.716
**Kushtia**	2.003	2.207	26.827	13.084	0.000	2.452	33.866	6.518	4.700	6.132
**Lakshmipur**	3.918	2.798	25.078	19.240	3.794	4.035	27.244	3.507	3.378	7.008
**Lalmonirhat**	2.098	2.034	25.408	15.874	5.007	1.901	30.556	4.563	2.336	10.224
**Madaripur**	1.785	0.000	27.729	12.350	5.982	2.273	29.990	4.941	3.472	9.420
**Magura**	3.493	0.000	22.059	19.018	5.417	3.927	25.781	3.906	6.537	7.955
**Manikganj**	2.205	2.082	25.771	15.859	3.376	1.598	28.599	4.855	7.910	7.746
**Meherpur**	1.933	0.000	26.381	12.590	5.990	1.857	31.763	5.347	6.494	5.465
**Moulvibazar**	0.000	18.944	19.634	14.102	6.526	2.982	20.344	3.022	4.060	7.702
**Munshiganj**	1.930	0.000	24.834	15.091	7.380	0.000	29.040	4.744	3.438	9.237
**Mymensingh**	1.562	6.347	16.950	8.728	1.955	0.000	19.088	3.061	1.540	38.023
**Naogaon**	3.951	1.988	22.870	20.683	4.021	4.554	29.357	4.302	0.000	3.764
**Narail**	1.714	1.649	22.568	14.192	6.779	0.000	26.782	4.868	5.166	13.816
**Narayanganj**	2.112	0.000	24.628	11.637	9.126	0.000	29.646	5.114	2.682	7.787
**Narsingdi**	4.161	2.454	24.419	21.822	4.981	4.581	27.768	4.189	3.191	0.000
**Natore**	2.191	3.307	25.802	16.244	1.828	2.286	32.011	6.100	2.401	7.829
**Netrokona**	2.275	9.253	28.049	13.041	5.723	0.000	29.445	4.491	3.352	2.331
**Nilphamari**	4.050	2.052	23.893	21.038	1.572	4.395	28.102	3.802	5.841	5.256
**Noakhali**	2.332	2.983	27.869	15.647	5.253	0.000	28.082	4.152	4.165	7.526
**Pabna**	2.263	4.049	27.711	13.101	5.334	0.000	32.394	5.168	5.132	0.000
**Panchagarh**	3.612	2.013	21.926	20.115	3.012	4.277	27.290	3.888	5.048	8.817
**Patuakhali**	1.923	8.387	23.814	14.866	5.157	0.000	27.655	4.265	3.318	8.641
**Pirojpur**	1.807	8.407	24.311	11.897	4.342	1.832	26.672	4.558	4.329	11.846
**Rajbari**	3.349	0.000	22.486	18.141	4.886	3.811	26.160	4.327	3.653	11.537
**Rajshahi**	0.000	3.240	25.331	16.229	5.738	0.000	30.054	6.143	2.237	4.374
**Rangamati**	2.171	2.659	23.220	11.724	7.540	0.000	26.467	3.641	6.189	11.936
**Rangpur**	3.812	0.000	21.472	21.338	4.583	4.489	28.099	3.916	2.734	5.929
**Satkhira**	2.127	1.853	24.653	15.850	4.502	1.875	31.662	5.302	2.563	9.612
**Shariatpur**	2.376	2.425	27.194	14.077	5.893	0.000	32.262	5.271	3.320	5.026
**Sherpur**	3.889	7.884	21.636	19.036	2.977	3.747	25.210	3.226	5.673	6.722
**Sirajganj**	0.000	3.238	25.493	14.073	5.486	0.000	29.757	4.303	4.220	8.141
**Sunamganj**	1.633	25.050	23.583	9.437	2.545	1.442	26.556	3.774	4.511	0.000
**Sylhet**	2.840	17.833	19.290	15.309	3.118	3.339	22.383	3.169	1.377	11.342
**Tangail**	1.913	1.949	21.275	13.393	3.698	0.000	26.374	3.957	2.208	20.951
**Thakurgaon**	2.185	2.020	24.554	13.028	0.000	2.225	32.047	6.035	4.390	11.312

X24: Monthly relative humidity at 3 meters (%); X216: Monthly domestic general government health expenditure (%); X22: Minimum monthly temperature at 3 meters (C); X28: Total monthly population (each district); X212: Average monthly household size; X25: Monthly rainfall

corrected (mm/day); X220: Monthly arable land; X26: Monthly surface pressure (kPa); X21: Monthly average temperature at 2 meters (C); X23: Maximum monthly temperature at 3 meters (C);

Table S30. Top contribution of the catagorieson prediction of district wise monthly dengue cases

District	Climate & Environmental Factors	Socio-Demographic & Economic Indicators	Healthcare System Capacity & Resources	Land Use and Land Cover Indicators
Bagerhat	57.922	11.941	25.692	4.444
Bandarban	61.423	14.862	20.709	3.005
Barguna	52.531	19.804	23.626	4.039
Barishal	53.969	13.564	26.027	6.439
Bhola	59.071	16.701	21.195	3.034
Bogura	49.712	25.094	19.189	6.005
Brahmanbaria	57.713	11.515	27.645	3.127
Chandpur	64.809	7.395	25.305	2.492
Chapai Nawabganj	58.157	14.122	26.215	1.506
Chattogram	26.129	58.304	11.843	3.724
Chuadanga	60.941	10.677	22.757	5.626
Cox's Bazar	36.798	42.967	16.053	4.183
Cumilla	57.449	7.765	27.933	6.853
Dhaka	18.878	72.515	7.822	0.786
Dinajpur	55.848	16.478	23.606	4.068
Faridpur	58.106	12.572	24.782	4.541
Feni	56.944	12.216	22.404	8.436
Gaibandha	59.021	8.848	26.124	6.006
Gazipur	50.971	25.032	21.879	2.118
Gopalganj	62.168	13.614	21.625	2.593
Habiganj	41.987	29.770	22.051	6.192
Jamalpur	53.795	13.862	27.414	4.929
Jashore	62.422	8.569	21.917	7.092
Jhalokathi	50.058	18.025	21.236	10.681
Jhenaidah	61.139	9.398	25.740	3.723
Joypurhat	59.817	12.117	19.857	8.209
Khagrachhari	52.618	15.951	24.620	6.811
Khulna	59.871	9.516	26.123	4.490
Kishorganj	59.610	14.089	19.619	6.682
Kurigram	58.526	10.396	24.193	6.885
Kushtia	61.395	11.006	26.301	1.298
Lakshmipur	59.459	11.389	24.317	4.835
Lalmonirhat	55.422	13.808	24.563	6.207
Madaripur	53.363	11.814	26.996	7.827
Magura	60.835	10.639	21.416	7.110
Manikganj	59.366	11.458	25.071	4.105
Meherpur	57.885	8.773	25.458	7.884

Moulvibazar	44.294	28.515	18.509	8.682
Munshiganj	53.684	12.576	23.928	9.812
Mymensingh	34.199	46.815	16.599	2.387
Naogaon	62.434	11.014	22.063	4.489
Narail	52.465	16.768	21.824	8.942
Narayanganj	50.821	13.280	23.509	12.391
Narsingdi	62.750	7.557	23.319	6.374
Natore	58.748	13.381	24.754	3.117
Netrokona	51.987	13.968	26.820	7.226
Nilphamari	65.574	9.245	23.305	1.876
Noakhali	54.162	11.945	26.901	6.992
Pabna	57.132	8.860	26.398	7.610
Panchagarh	62.667	12.256	21.392	3.686
Patuakhali	52.143	17.928	23.033	6.896
Pirojpur	49.614	21.235	23.607	5.544
Rajbari	57.662	14.184	21.814	6.341
Rajshahi	56.030	11.939	24.088	7.944
Rangamati	49.919	15.959	22.501	11.622
Rangpur	60.954	12.256	20.327	6.464
Satkhira	57.809	13.376	24.002	4.813
Shariatpur	56.586	10.003	25.890	7.521
Sherpur	58.913	16.507	20.970	3.610
Sirajganj	53.666	14.520	24.327	7.486
Sunamganj	45.977	28.219	22.898	2.906
Sylhet	47.182	30.597	18.797	3.423
Tangail	47.769	27.257	20.641	4.333
Thakurgaon	58.172	15.876	23.842	2.110

Table S31. Senario analysis of the top 10 features on monthly dengue prediction

Feature	X21	X212	X216	X22	X220	X23	X24	X25	X26	X28
**Change_%**	**Predicted Probability**	**Predicted Probability**	**Predicted Probability**	**Predicted Probability**	**Predicted Probability**	**Predicted Probability**	**Predicted Probability**	**Predicted Probability**	**Predicted Probability**	**Predicted Probability**
-50	0.626	0.648	0.549	0.669	0.578	0.581	0.694	0.625	0.598	0.603
-40	0.622	0.642	0.555	0.647	0.579	0.585	0.674	0.624	0.596	0.603
-30	0.621	0.630	0.566	0.639	0.589	0.590	0.652	0.622	0.598	0.605
-20	0.617	0.620	0.581	0.629	0.594	0.592	0.629	0.621	0.603	0.608
-10	0.612	0.613	0.595	0.620	0.604	0.599	0.615	0.613	0.603	0.605
0	0.608	0.608	0.608	0.608	0.608	0.608	0.608	0.608	0.608	0.608
10	0.603	0.600	0.620	0.598	0.615	0.611	0.596	0.604	0.611	0.612
20	0.599	0.590	0.634	0.589	0.618	0.612	0.585	0.599	0.615	0.613
30	0.595	0.582	0.643	0.577	0.625	0.615	0.574	0.599	0.620	0.613

40	0.594	0.568	0.660	0.570	0.632	0.621	0.564	0.590	0.626	0.615
50	0.578	0.552	0.671	0.566	0.637	0.625	0.564	0.587	0.632	0.617

X24: Monthly relative humidity at 3 meters (%); X216: Monthly domestic general government health expenditure (%); X22: Minimum monthly temperature at 3 meters (C); X28: Total monthly population (each district); X212: Average monthly household size; X25: Monthly rainfall corrected (mm/day); X220: Monthly arable land; X26: Monthly surface pressure (kPa); X21: Monthly average temperature at 2 meters (C); X23: Maximum monthly temperature at 3 meters (C);

Table S32. Posterior estimates of fixed effects for the IID_RW1_Lag123 model using standardized monthly predictors.

Variable	mean	sd	0.025quant	0.5quant	0.975quant	RR	RR_lower	RR_upper
X24	0.064	0.061	-0.055	0.064	0.183	1.066	0.946	1.200
X216	-0.898	0.524	-1.931	-0.898	0.135	0.407	0.145	1.144
X22	0.153	0.189	-0.217	0.153	0.523	1.165	0.805	1.687
X212	-0.060	0.230	-0.513	-0.060	0.394	0.942	0.599	1.482
X25	-0.021	0.045	-0.109	-0.021	0.067	0.980	0.897	1.070
X220	-0.145	0.230	-0.598	-0.145	0.308	0.865	0.550	1.360
X26	0.060	0.022	0.017	0.060	0.104	1.062	1.017	1.110
X21	-0.101	0.249	-0.589	-0.101	0.387	0.904	0.555	1.473
X23	0.081	0.109	-0.134	0.081	0.295	1.084	0.875	1.344
lag1	0.000	0.000	0.000	0.000	0.000	1.000	1.000	1.000
lag2	0.000	0.000	0.000	0.000	0.000	1.000	1.000	1.000
lag3	0.000	0.000	0.000	0.000	0.000	1.000	1.000	1.000

mean = *posterior mean;* sd = *posterior standard deviation;* 0.025quant = *2.5th percentile of the posterior distribution;* 0.5quant = *median of the posterior distribution;* 0.975quant = *97.5th percentile of the posterior distribution;* mode = *posterior mode (highest density);* kld = *Kullback–Leibler divergence, quantifying the difference between the posterior and prior distributions; X24: Monthly relative humidity at 3 meters (%); X216: Monthly domestic general government health expenditure (%); X22: Minimum monthly temperature at 3 meters (C); X212: Average monthly household size; X25: Monthly rainfall corrected (mm/day); X220: Monthly arable land; X26: Monthly surface pressure (kPa); X21: Monthly average temperature at 2 meters (C); X23: Maximum monthly temperature at 3 meters (C); lag1: 1-month lag of dengue cases; lag2: 2-month lag of dengue cases; lag3: 3-month lag of dengue cases;*

Table S33. Posterior estimates of random effects for the IID_RW1_Lag123 model using standardized monthly predictors.

**District**	**mean**	**sd**	**0.025quant**	**0.5quant**	**0.975quant**	**mode**	**kld**
BAGERHAT	-0.483	0.471	-1.410	-0.483	0.443	-0.483	0.000
BANDARBAN	1.158	0.558	0.062	1.158	2.257	1.158	0.000
BARGUNA	1.833	0.336	1.173	1.833	2.496	1.833	0.000
BHOLA	0.676	0.282	0.122	0.675	1.230	0.675	0.000
CHANDPUR	1.780	0.431	0.933	1.780	2.629	1.780	0.000
CHUADANGA	-1.971	0.343	-2.647	-1.970	-1.298	-1.970	0.000

COX'S BAZAR	3.615	0.372	2.885	3.614	4.349	3.614	0.000
DHAKA	5.530	0.311	4.922	5.529	6.144	5.529	0.000
DINAJPUR	-0.219	0.423	-1.052	-0.219	0.614	-0.219	0.000
FARIDPUR	0.599	0.291	0.026	0.599	1.172	0.599	0.000
FENI	0.429	0.509	-0.572	0.429	1.432	0.429	0.000
GAIBANDHA	-1.786	0.291	-2.359	-1.786	-1.216	-1.786	0.000
GAZIPUR	1.247	0.291	0.675	1.247	1.821	1.247	0.000
GOPALGANJ	-0.755	0.310	-1.365	-0.755	-0.147	-0.755	0.000
HABIGANJ	-1.835	0.716	-3.247	-1.835	-0.426	-1.835	0.000
JAMALPUR	-0.431	0.283	-0.987	-0.431	0.124	-0.431	0.000
JHENAIDAH	-0.111	0.294	-0.689	-0.111	0.466	-0.111	0.000
JOYPURHAT	-2.567	0.380	-3.316	-2.566	-1.821	-2.566	0.000
KHAGRACHHARI	-0.190	0.370	-0.917	-0.190	0.537	-0.190	0.000
KHULNA	1.707	0.461	0.801	1.707	2.616	1.707	0.000
KURIGRAM	-1.523	0.296	-2.106	-1.522	-0.941	-1.522	0.000
KUSHTIA	0.202	0.299	-0.385	0.202	0.790	0.202	0.000
LAKSHMIPUR	1.062	0.460	0.158	1.062	1.968	1.062	0.000
LALMONIRHAT	-2.681	0.335	-3.342	-2.680	-2.024	-2.680	0.000
MADARIPUR	0.902	0.334	0.245	0.902	1.560	0.902	0.000
MAGURA	-0.237	0.347	-0.920	-0.237	0.445	-0.237	0.000
MANIKGANJ	0.816	0.312	0.202	0.816	1.430	0.816	0.000
MEHERPUR	-0.168	0.336	-0.829	-0.168	0.493	-0.168	0.000
MUNSHIGANJ	-0.070	0.362	-0.782	-0.070	0.642	-0.070	0.000
MYMENSINGH	1.743	0.510	0.740	1.742	2.747	1.742	0.000
NAOGAON	-0.549	0.455	-1.445	-0.549	0.346	-0.549	0.000
NARAIL	0.706	0.351	0.016	0.706	1.397	0.706	0.000
NARAYANGANJ	0.513	0.389	-0.251	0.513	1.278	0.513	0.000
NARSINGDI	1.102	0.331	0.452	1.102	1.753	1.102	0.000
NATORE	-1.237	0.313	-1.853	-1.236	-0.622	-1.236	0.000
NILPHAMARI	-1.458	0.299	-2.047	-1.458	-0.871	-1.458	0.000
NOAKHALI	0.068	0.365	-0.650	0.068	0.787	0.068	0.000

PABNA	0.409	0.323	-0.226	0.409	1.044	0.409	0.000
PANCHAGARH	-2.778	0.328	-3.425	-2.778	-2.135	-2.778	0.000
PATUAKHALI	1.244	0.281	0.693	1.244	1.796	1.244	0.000
PIROJPUR	1.049	0.347	0.367	1.049	1.733	1.049	0.000
RAJBARI	-0.477	0.338	-1.143	-0.477	0.188	-0.477	0.000
RAJSHAHI	0.501	0.326	-0.141	0.501	1.143	0.501	0.000
RANGAMATI	-0.911	0.765	-2.418	-0.911	0.595	-0.911	0.000
RANGPUR	-0.739	0.295	-1.318	-0.739	-0.160	-0.739	0.000
SATKHIRA	-0.134	0.431	-0.981	-0.134	0.713	-0.134	0.000
SHARIATPUR	0.022	0.333	-0.632	0.022	0.676	0.022	0.000
SHERPUR	-1.033	0.350	-1.722	-1.033	-0.346	-1.033	0.000
SIRAJGANJ	-0.066	0.332	-0.718	-0.066	0.587	-0.066	0.000
SUNAMGANJ	-2.602	0.717	-4.015	-2.601	-1.194	-2.601	0.000
SYLHET	0.056	0.701	-1.323	0.056	1.436	0.056	0.000
TANGAIL	0.325	0.423	-0.507	0.325	1.157	0.325	0.000
THAKURGAON	-2.540	0.301	-3.133	-2.539	-1.949	-2.539	0.000

Table S34. Posterior estimates of temporal effects for the IID_RW1_Lag123 model using standardized monthly predictors.

MonthYear	mean	sd	0.025quant	0.5quant	0.975quant	mode	kld
2022-01	-5.149	0.778	-6.685	-5.148	-3.619	-5.148	0.000
2022-02	-4.908	0.777	-6.442	-4.907	-3.380	-4.907	0.000
2022-03	-4.928	0.775	-6.458	-4.927	-3.404	-4.927	0.000
2022-04	-4.910	0.771	-6.432	-4.909	-3.394	-4.909	0.000
2022-05	-4.759	0.768	-6.280	-4.757	-3.254	-4.757	0.000
2022-06	-2.326	0.751	-3.802	-2.328	-0.840	-2.328	0.000
2022-07	-3.469	0.755	-4.957	-3.469	-1.981	-3.469	0.000
2022-08	-2.738	0.752	-4.219	-2.738	-1.253	-2.738	0.000
2022-09	-1.060	0.750	-2.536	-1.061	0.423	-1.061	0.000

2022-10	0.007	0.749	-1.467	0.006	1.487	0.006	0.000
2022-11	0.167	0.748	-1.306	0.166	1.647	0.166	0.000
2022-12	-1.277	0.738	-2.736	-1.276	0.178	-1.276	0.000
2023-01	-1.282	0.383	-2.039	-1.281	-0.528	-1.281	0.000
2023-02	-1.097	0.402	-1.891	-1.096	-0.308	-1.096	0.000
2023-03	-1.090	0.402	-1.884	-1.089	-0.300	-1.089	0.000
2023-04	-1.080	0.399	-1.867	-1.079	-0.297	-1.079	0.000
2023-05	-0.829	0.400	-1.618	-0.828	-0.044	-0.828	0.000
2023-06	0.410	0.394	-0.366	0.410	1.185	0.410	0.000
2023-07	3.492	0.395	2.719	3.490	4.274	3.490	0.000
2023-08	4.641	0.395	3.866	4.639	5.422	4.639	0.000
2023-09	5.202	0.395	4.428	5.201	5.984	5.201	0.000
2023-10	4.418	0.393	3.645	4.417	5.195	4.417	0.000
2023-11	3.965	0.390	3.200	3.965	4.737	3.965	0.000
2023-12	2.476	0.388	1.713	2.476	3.242	2.476	0.000
2024-01	-0.067	0.396	-0.852	-0.066	0.709	-0.066	0.000
2024-02	0.151	0.397	-0.634	0.152	0.930	0.152	0.000
2024-03	0.272	0.397	-0.512	0.273	1.051	0.273	0.000
2024-04	0.438	0.393	-0.338	0.439	1.210	0.439	0.000
2024-05	0.576	0.396	-0.205	0.576	1.354	0.576	0.000
2024-06	0.667	0.393	-0.107	0.667	1.442	0.667	0.000
2024-07	1.020	0.396	0.243	1.019	1.802	1.019	0.000
2024-08	1.538	0.395	0.761	1.537	2.319	1.537	0.000
2024-09	2.779	0.395	2.005	2.778	3.560	2.778	0.000
2024-10	3.229	0.392	2.458	3.228	4.004	3.228	0.000
2024-11	3.336	0.389	2.572	3.335	4.104	3.335	0.000
2024-12	2.184	0.388	1.418	2.184	2.947	2.184	0.000

mean = *posterior mean;* sd = *posterior standard deviation;* 0.025quant = *2.5th percentile of the posterior distribution;* 0.5quant = *median of the posterior distribution;* 0.975quant = *97.5th percentile of the posterior distribution;* mode = *posterior mode (highest density);* kld = *Kullback–Leibler divergence, quantifying the difference between the posterior and prior distributions.*

Table S35. District wise monthly early warning for dengue outbreak (2025-26) by using ConvLSTM

Month		January		February		March		April		May		June		July		August		September		October		November		December	
District	Year	Prob High Dengue	High Alert	Prob High Dengue	High_Alert	Prob_High_Dengue	High_Alert	Prob_High_Dengue	High_Alert	Prob_High_Dengue	High_Alert	Prob_High_Dengue	High_Alert	Prob_High_Dengue	High_Alert	Prob_High_Dengue	High_Alert	Prob_High_Dengue	High_Alert	Prob_High_Dengue	High_Alert	Prob_High_Dengue	High_Alert	Prob_High_Dengue	High_Alert
Bagerhat	2025	0.699	1	0.256	0	0.070	0	0.019	0	0.010	0	0.005	0	0.003	0	0.002	0	0.002	0	0.002	0	0.001	0	0.001	0
Bandarban	2025	0.976	1	0.997	1	0.987	1	0.742	1	0.589	1	0.367	0	0.164	0	0.119	0	0.275	0	0.641	1	0.737	1	0.797	1
Barguna	2025	0.833	1	0.671	1	0.247	0	0.075	0	0.012	0	0.001	0	0.000	0	0.000	0	0.000	0	0.000	0	0.000	0	0.000	0
Barishal	2025	0.926	1	0.702	1	0.550	1	0.297	0	0.273	0	0.132	0	0.071	0	0.028	0	0.005	0	0.002	0	0.000	0	0.000	0
Bhola	2025	0.504	1	0.781	1	0.565	1	0.059	0	0.022	0	0.029	0	0.067	0	0.171	0	0.215	0	0.228	0	0.216	0	0.257	0
Bogura	2025	0.926	1	0.919	1	0.767	1	0.419	0	0.115	0	0.025	0	0.019	0	0.005	0	0.002	0	0.002	0	0.004	0	0.001	0
Brahmanbaria	2025	0.544	1	0.225	0	0.114	0	0.059	0	0.054	0	0.038	0	0.029	0	0.021	0	0.019	0	0.020	0	0.017	0	0.015	0
Chandpur	2025	0.866	1	0.932	1	0.737	1	0.382	0	0.443	0	0.563	1	0.599	1	0.807	1	0.826	1	0.776	1	0.594	1	0.555	1
Chapai Nawabganj	2025	0.330	0	0.317	0	0.142	0	0.050	0	0.025	0	0.014	0	0.009	0	0.007	0	0.006	0	0.006	0	0.008	0	0.008	0
Chattogram	2025	1.000	1	1.000	1	1.000	1	1.000	1	1.000	1	1.000	1	1.000	1	1.000	1	1.000	1	1.000	1	1.000	1	1.000	1

Chuadanga	2025	0.369	0	0.811	1	0.536	1	0.334	0	0.388	0	0.440	0	0.354	0	0.188	0	0.320	0	0.346	0	0.294	0	0.345	0
Cox's Bazar	2025	1.000	1	1.000	1	1.000	1	1.000	1	1.000	1	1.000	1	1.000	1	1.000	1	1.000	1	1.000	1	1.000	1	1.000	1
Cumilla	2025	0.997	1	0.991	1	0.971	1	0.903	1	0.828	1	0.831	1	0.847	1	0.789	1	0.744	1	0.702	1	0.508	1	0.664	1
Dhaka	2025	1.000	1	1.000	1	1.000	1	1.000	1	1.000	1	1.000	1	1.000	1	1.000	1	1.000	1	1.000	1	1.000	1	1.000	1
Dinajpur	2025	0.574	1	0.474	0	0.151	0	0.056	0	0.028	0	0.020	0	0.024	0	0.020	0	0.022	0	0.017	0	0.017	0	0.027	0
Faridpur	2025	0.306	0	0.104	0	0.061	0	0.021	0	0.017	0	0.015	0	0.013	0	0.008	0	0.007	0	0.003	0	0.001	0	0.001	0
Feni	2025	0.666	1	0.948	1	0.887	1	0.458	0	0.614	1	0.851	1	0.894	1	0.949	1	0.962	1	0.961	1	0.852	1	0.550	1
Gaibandha	2025	0.263	0	0.189	0	0.052	0	0.016	0	0.007	0	0.004	0	0.003	0	0.002	0	0.002	0	0.001	0	0.001	0	0.001	0
Gazipur	2025	0.967	1	0.901	1	0.754	1	0.556	1	0.561	1	0.446	0	0.397	0	0.315	0	0.259	0	0.307	0	0.231	0	0.253	0
Gopalganj	2025	0.175	0	0.461	0	0.332	0	0.118	0	0.126	0	0.209	0	0.178	0	0.124	0	0.126	0	0.127	0	0.137	0	0.151	0
Habiganj	2025	0.004	0	0.002	0	0.001	0	0.000	0	0.000	0	0.000	0	0.000	0	0.000	0	0.000	0	0.000	0	0.000	0	0.000	0
Jamalpur	2025	0.964	1	0.856	1	0.647	1	0.412	0	0.350	0	0.233	0	0.223	0	0.190	0	0.176	0	0.238	0	0.309	0	0.440	0
Jashore	20	0.556	1	0.899	1	0.810	1	0.500	0	0.593	1	0.655	1	0.648	1	0.554	1	0.656	1	0.653	1	0.541	1	0.539	1

	25																										
Jhalokathi	2025	0.865	1	0.932	1	0.781	1	0.397	0	0.265	0	0.287	0	0.265	0	0.332	0	0.339	0	0.558	1	0.839	1	0.937	1		
Jhenaidah	2025	0.389	0	0.167	0	0.090	0	0.029	0	0.023	0	0.018	0	0.016	0	0.014	0	0.011	0	0.013	0	0.012	0	0.013	0		
Joypurhat	2025	0.297	0	0.726	1	0.573	1	0.346	0	0.446	0	0.568	1	0.564	1	0.549	1	0.618	1	0.625	1	0.614	1	0.511	1		
Khagrachhari	2025	0.920	1	0.884	1	0.456	0	0.090	0	0.013	0	0.002	0	0.000	0	0.000	0	0.001	0	0.000	0	0.000	0	0.000	0		
Khulna	2025	0.610	1	0.236	0	0.140	0	0.054	0	0.043	0	0.029	0	0.020	0	0.015	0	0.014	0	0.011	0	0.009	0	0.003	0		
Kishorganj	2025	0.681	1	0.890	1	0.796	1	0.386	0	0.309	0	0.470	0	0.559	1	0.674	1	0.557	1	0.420	0	0.422	0	0.396	0		
Kurigram	2025	0.183	0	0.108	0	0.030	0	0.011	0	0.004	0	0.003	0	0.001	0	0.001	0	0.000	0	0.000	0	0.000	0	0.000	0		
Kushtia	2025	0.488	0	0.262	0	0.094	0	0.036	0	0.023	0	0.018	0	0.015	0	0.010	0	0.009	0	0.006	0	0.005	0	0.005	0		
Lakshmipur	2025	0.913	1	0.964	1	0.892	1	0.601	1	0.627	1	0.866	1	0.930	1	0.978	1	0.981	1	0.967	1	0.884	1	0.803	1		
Lalmonirhat	2025	0.764	1	0.741	1	0.397	0	0.153	0	0.038	0	0.010	0	0.002	0	0.001	0	0.000	0	0.000	0	0.000	0	0.000	0		
Madaripur	2025	0.701	1	0.417	0	0.199	0	0.067	0	0.028	0	0.010	0	0.004	0	0.004	0	0.002	0	0.000	0	0.000	0	0.000	0		
Magura	2025	0.260	0	0.442	0	0.187	0	0.077	0	0.035	0	0.013	0	0.006	0	0.004	0	0.007	0	0.009	0	0.005	0	0.002	0		

Manikganj	2025	0.309	0	0.230	0	0.093	0	0.022	0	0.006	0	0.003	0	0.002	0	0.003	0	0.002	0	0.003	0	0.002	0	0.001	0
Meherpur	2025	0.741	1	0.339	0	0.103	0	0.032	0	0.017	0	0.008	0	0.005	0	0.004	0	0.003	0	0.003	0	0.001	0	0.001	0
Moulvibazar	2025	0.087	0	0.219	0	0.059	0	0.011	0	0.003	0	0.001	0	0.000	0	0.000	0	0.000	0	0.002	0	0.021	0	0.192	0
Munshiganj	2025	0.959	1	0.942	1	0.757	1	0.504	1	0.188	0	0.032	0	0.004	0	0.001	0	0.000	0	0.000	0	0.000	0	0.000	0
Mymensingh	2025	1.000	1	1.000	1	1.000	1	1.000	1	1.000	1	0.999	1	0.999	1	0.999	1	0.991	1	0.993	1	0.974	1	0.993	1
Naogaon	2025	0.564	1	0.871	1	0.828	1	0.171	0	0.056	0	0.073	0	0.145	0	0.243	0	0.239	0	0.221	0	0.221	0	0.216	0
Narail	2025	0.844	1	0.779	1	0.321	0	0.129	0	0.036	0	0.008	0	0.002	0	0.000	0	0.000	0	0.000	0	0.000	0	0.000	0
Narayanganj	2025	0.999	1	0.998	1	0.993	1	0.985	1	0.984	1	0.980	1	0.974	1	0.963	1	0.958	1	0.954	1	0.951	1	0.950	1
Narsingdi	2025	0.830	1	0.930	1	0.822	1	0.557	1	0.777	1	0.862	1	0.877	1	0.938	1	0.950	1	0.958	1	0.954	1	0.948	1
Natore	2025	0.224	0	0.195	0	0.074	0	0.027	0	0.013	0	0.007	0	0.005	0	0.004	0	0.004	0	0.005	0	0.006	0	0.006	0
Netrokona	2025	0.986	1	0.921	1	0.797	1	0.571	1	0.482	0	0.380	0	0.318	0	0.284	0	0.284	0	0.290	0	0.270	0	0.244	0
Nilphamari	2025	0.504	1	0.909	1	0.728	1	0.409	0	0.521	1	0.604	1	0.513	1	0.326	0	0.526	1	0.613	1	0.524	1	0.572	1
Noakhali	20	0.996	1	0.997	1	0.990	1	0.958	1	0.859	1	0.859	1	0.857	1	0.906	1	0.925	1	0.918	1	0.927	1	0.969	1

	25																								
Pabna	2025	0.600	1	0.451	0	0.344	0	0.110	0	0.079	0	0.067	0	0.067	0	0.057	0	0.050	0	0.077	0	0.083	0	0.135	0
Panchagarh	2025	0.259	0	0.730	1	0.582	1	0.258	0	0.240	0	0.242	0	0.152	0	0.060	0	0.075	0	0.062	0	0.059	0	0.070	0
Patuakhali	2025	0.640	1	0.506	1	0.097	0	0.022	0	0.008	0	0.005	0	0.006	0	0.004	0	0.005	0	0.003	0	0.002	0	0.005	0
Pirojpur	2025	0.965	1	0.924	1	0.760	1	0.432	0	0.201	0	0.156	0	0.130	0	0.083	0	0.063	0	0.017	0	0.003	0	0.003	0
Rajbari	2025	0.017	0	0.251	0	0.328	0	0.065	0	0.118	0	0.344	0	0.473	0	0.574	1	0.702	1	0.765	1	0.580	1	0.337	0
Rajshahi	2025	0.356	0	0.252	0	0.074	0	0.021	0	0.009	0	0.005	0	0.003	0	0.002	0	0.002	0	0.002	0	0.001	0	0.002	0
Rangamati	2025	0.272	0	0.113	0	0.025	0	0.004	0	0.002	0	0.001	0	0.001	0	0.001	0	0.000	0	0.001	0	0.001	0	0.001	0
Rangpur	2025	0.246	0	0.417	0	0.269	0	0.112	0	0.123	0	0.188	0	0.226	0	0.280	0	0.289	0	0.326	0	0.347	0	0.381	0
Satkhira	2025	0.219	0	0.261	0	0.073	0	0.021	0	0.008	0	0.006	0	0.005	0	0.004	0	0.003	0	0.004	0	0.007	0	0.011	0
Shariatpur	2025	0.948	1	0.915	1	0.807	1	0.598	1	0.485	0	0.394	0	0.365	0	0.331	0	0.283	0	0.333	0	0.421	0	0.565	1
Sherpur	2025	0.910	1	0.985	1	0.963	1	0.816	1	0.849	1	0.914	1	0.900	1	0.902	1	0.955	1	0.947	1	0.887	1	0.856	1
Sirajganj	2025	0.824	1	0.884	1	0.822	1	0.483	0	0.295	0	0.253	0	0.255	0	0.354	0	0.448	0	0.760	1	0.957	1	0.984	1

Sunamganj	2025	0.013	0	0.003	0	0.001	0	0.000	0	0.000	0	0.000	0	0.000	0	0.000	0	0.000	0	0.000	0	0.000	0	0.000	0
Sylhet	2025	0.121	0	0.273	0	0.139	0	0.038	0	0.017	0	0.006	0	0.005	0	0.005	0	0.006	0	0.006	0	0.005	0	0.005	0
Tangail	2025	0.882	1	0.832	1	0.532	1	0.247	0	0.141	0	0.061	0	0.030	0	0.028	0	0.026	0	0.018	0	0.010	0	0.003	0
Thakurgaon	2025	0.162	0	0.036	0	0.040	0	0.020	0	0.016	0	0.011	0	0.004	0	0.003	0	0.003	0	0.002	0	0.001	0	0.000	0
Bagerhat	2026	0.217	0	0.184	0	0.246	0	0.363	0	0.532	1	0.784	1	0.831	1	0.896	1	0.876	1	0.905	1	0.930	1	0.945	1
Bandarban	2026	0.047	0	0.480	0	0.628	1	0.539	1	0.388	0	0.295	0	0.132	0	0.057	0	0.082	0	0.100	0	0.129	0	0.575	1
Barguna	2026	0.264	0	0.314	0	0.288	0	0.363	0	0.484	0	0.683	1	0.913	1	0.942	1	0.947	1	0.918	1	0.859	1	0.842	1
Barishal	2026	0.115	0	0.187	0	0.444	0	0.788	1	0.919	1	0.944	1	0.964	1	0.979	1	0.984	1	0.982	1	0.981	1	0.977	1
Bhola	2026	0.010	0	0.020	0	0.028	0	0.072	0	0.036	0	0.040	0	0.024	0	0.005	0	0.003	0	0.002	0	0.001	0	0.002	0
Bogura	2026	0.325	0	0.402	0	0.508	1	0.788	1	0.957	1	0.990	1	0.997	1	0.998	1	0.999	1	0.998	1	0.995	1	0.993	1
Brahmanbaria	2026	0.063	0	0.064	0	0.139	0	0.331	0	0.587	1	0.774	1	0.811	1	0.819	1	0.779	1	0.769	1	0.795	1	0.805	1
Chandpur	2026	0.077	0	0.286	0	0.504	1	0.669	1	0.751	1	0.692	1	0.682	1	0.541	1	0.321	0	0.265	0	0.154	0	0.075	0
Chapai	20	0.125	0	0.109	0	0.119	0	0.187	0	0.295	0	0.568	1	0.719	1	0.774	1	0.836	1	0.809	1	0.789	1	0.825	1

Nawabganj	26																								
Chattogram	2026	1.000	1	1.000	1	1.000	1	1.000	1	1.000	1	1.000	1	1.000	1	1.000	1	1.000	1	1.000	1	1.000	1	1.000	1
Chuadanga	2026	0.027	0	0.194	0	0.165	0	0.065	0	0.072	0	0.062	0	0.099	0	0.087	0	0.065	0	0.069	0	0.063	0	0.037	0
Cox's Bazar	2026	1.000	1	1.000	1	1.000	1	1.000	1	1.000	1	1.000	1	1.000	1	1.000	1	1.000	1	1.000	1	1.000	1	1.000	1
Cumilla	2026	0.979	1	0.990	1	0.991	1	0.994	1	0.998	1	0.998	1	0.997	1	0.998	1	0.994	1	0.993	1	0.995	1	0.988	1
Dhaka	2026	1.000	1	1.000	1	1.000	1	1.000	1	1.000	1	1.000	1	1.000	1	1.000	1	1.000	1	1.000	1	1.000	1	1.000	1
Dinajpur	2026	0.133	0	0.166	0	0.158	0	0.284	0	0.505	1	0.777	1	0.875	1	0.908	1	0.924	1	0.902	1	0.893	1	0.922	1
Faridpur	2026	0.038	0	0.050	0	0.127	0	0.296	0	0.494	0	0.564	1	0.535	1	0.536	1	0.504	1	0.527	1	0.562	1	0.598	1
Feni	2026	0.122	0	0.170	0	0.524	1	0.663	1	0.911	1	0.971	1	0.965	1	0.913	1	0.791	1	0.719	1	0.306	0	0.035	0
Gaibandha	2026	0.054	0	0.071	0	0.114	0	0.230	0	0.475	0	0.695	1	0.694	1	0.694	1	0.715	1	0.670	1	0.661	1	0.728	1
Gazipur	2026	0.461	0	0.501	1	0.704	1	0.852	1	0.917	1	0.943	1	0.953	1	0.957	1	0.950	1	0.950	1	0.957	1	0.960	1
Gopalganj	2026	0.009	0	0.042	0	0.082	0	0.055	0	0.051	0	0.037	0	0.015	0	0.014	0	0.017	0	0.013	0	0.016	0	0.022	0
Habiganj	20	0.001	0	0.001	0	0.001	0	0.001	0	0.001	0	0.002	0	0.002	0	0.002	0	0.002	0	0.001	0	0.002	0	0.003	0

	26																								
Jamalpur	2026	0.701	1	0.732	1	0.834	1	0.888	1	0.937	1	0.975	1	0.975	1	0.977	1	0.964	1	0.958	1	0.969	1	0.975	1
Jashore	2026	0.043	0	0.429	0	0.622	1	0.395	0	0.351	0	0.402	0	0.471	0	0.413	0	0.343	0	0.365	0	0.334	0	0.268	0
Jhalokathi	2026	0.637	1	0.820	1	0.944	1	0.982	1	0.989	1	0.996	1	0.998	1	0.996	1	0.997	1	0.991	1	0.984	1	0.985	1
Jhenaidah	2026	0.169	0	0.223	0	0.243	0	0.440	0	0.660	1	0.800	1	0.801	1	0.828	1	0.770	1	0.787	1	0.839	1	0.854	1
Joypurhat	2026	0.058	0	0.219	0	0.270	0	0.168	0	0.197	0	0.282	0	0.263	0	0.216	0	0.135	0	0.104	0	0.065	0	0.035	0
Khagrachhari	2026	0.221	0	0.147	0	0.181	0	0.573	1	0.854	1	0.981	1	0.995	1	0.991	1	0.989	1	0.980	1	0.928	1	0.925	1
Khulna	2026	0.077	0	0.081	0	0.140	0	0.305	0	0.597	1	0.679	1	0.598	1	0.609	1	0.541	1	0.552	1	0.654	1	0.696	1
Kishorganj	2026	0.048	0	0.081	0	0.155	0	0.258	0	0.213	0	0.134	0	0.107	0	0.051	0	0.037	0	0.028	0	0.016	0	0.011	0
Kurigram	2026	0.032	0	0.048	0	0.079	0	0.186	0	0.507	1	0.739	1	0.754	1	0.681	1	0.673	1	0.554	1	0.504	1	0.526	1
Kushtia	2026	0.041	0	0.047	0	0.089	0	0.276	0	0.666	1	0.787	1	0.784	1	0.781	1	0.712	1	0.715	1	0.744	1	0.773	1
Lakshmipur	2026	0.084	0	0.459	0	0.646	1	0.849	1	0.915	1	0.895	1	0.805	1	0.649	1	0.354	0	0.197	0	0.065	0	0.018	0
Lalmonirhat	2026	0.397	0	0.352	0	0.250	0	0.344	0	0.429	0	0.561	1	0.851	1	0.866	1	0.804	1	0.758	1	0.445	0	0.253	0

Madaripur	2026	0.223	0	0.138	0	0.109	0	0.243	0	0.368	0	0.482	0	0.649	1	0.649	1	0.507	1	0.560	1	0.627	1	0.447	0
Magura	2026	0.036	0	0.181	0	0.035	0	0.012	0	0.005	0	0.003	0	0.004	0	0.002	0	0.001	0	0.001	0	0.000	0	0.000	0
Manikganj	2026	0.163	0	0.120	0	0.133	0	0.246	0	0.334	0	0.563	1	0.818	1	0.810	1	0.881	1	0.846	1	0.781	1	0.710	1
Meherpur	2026	0.295	0	0.269	0	0.339	0	0.434	0	0.609	1	0.855	1	0.915	1	0.958	1	0.952	1	0.963	1	0.967	1	0.965	1
Moulvibazar	2026	0.010	0	0.018	0	0.002	0	0.000	0	0.000	0	0.000	0	0.000	0	0.000	0	0.000	0	0.000	0	0.000	0	0.023	0
Munshiganj	2026	0.784	1	0.820	1	0.813	1	0.832	1	0.837	1	0.842	1	0.946	1	0.960	1	0.954	1	0.939	1	0.855	1	0.729	1
Mymensingh	2026	0.999	1	0.999	1	1.000	1	1.000	1	1.000	1	1.000	1	1.000	1	1.000	1	1.000	1	1.000	1	1.000	1	1.000	1
Naogaon	2026	0.019	0	0.063	0	0.073	0	0.084	0	0.062	0	0.049	0	0.022	0	0.008	0	0.006	0	0.003	0	0.002	0	0.002	0
Narail	2026	0.300	0	0.413	0	0.503	1	0.767	1	0.895	1	0.857	1	0.911	1	0.737	1	0.250	0	0.102	0	0.014	0	0.006	0
Narayanganj	2026	0.980	1	0.984	1	0.992	1	0.997	1	0.999	1	0.999	1	0.999	1	0.999	1	0.999	1	0.999	1	0.999	1	0.999	1
Narsingdi	2026	0.108	0	0.346	0	0.753	1	0.857	1	0.892	1	0.913	1	0.907	1	0.866	1	0.815	1	0.771	1	0.655	1	0.523	1
Natore	2026	0.080	0	0.067	0	0.062	0	0.113	0	0.211	0	0.436	0	0.603	1	0.655	1	0.743	1	0.715	1	0.696	1	0.771	1

Netrokona	2026	0.807	1	0.770	1	0.828	1	0.927	1	0.960	1	0.974	1	0.971	1	0.970	1	0.953	1	0.944	1	0.947	1	0.949	1
Nilphamari	2026	0.022	0	0.328	0	0.298	0	0.144	0	0.150	0	0.184	0	0.217	0	0.158	0	0.138	0	0.145	0	0.139	0	0.103	0
Noakhali	2026	0.991	1	0.996	1	0.996	1	0.998	1	0.999	1	0.999	1	1.000	1	0.999	1	0.999	1	0.997	1	0.996	1	0.993	1
Pabna	2026	0.348	0	0.455	0	0.570	1	0.677	1	0.852	1	0.925	1	0.917	1	0.933	1	0.906	1	0.901	1	0.935	1	0.940	1
Panchagarh	2026	0.011	0	0.138	0	0.076	0	0.028	0	0.021	0	0.023	0	0.012	0	0.016	0	0.015	0	0.012	0	0.017	0	0.016	0
Patuakhali	2026	0.103	0	0.132	0	0.131	0	0.252	0	0.619	1	0.869	1	0.951	1	0.967	1	0.953	1	0.922	1	0.907	1	0.888	1
Pirojpur	2026	0.422	0	0.549	1	0.830	1	0.956	1	0.982	1	0.976	1	0.961	1	0.953	1	0.899	1	0.910	1	0.923	1	0.886	1
Rajbari	2026	0.005	0	0.011	0	0.032	0	0.025	0	0.142	0	0.468	0	0.434	0	0.340	0	0.260	0	0.225	0	0.074	0	0.009	0
Rajshahi	2026	0.082	0	0.099	0	0.127	0	0.258	0	0.486	0	0.703	1	0.747	1	0.774	1	0.826	1	0.786	1	0.782	1	0.840	1
Rangamati	2026	0.002	0	0.002	0	0.004	0	0.015	0	0.070	0	0.195	0	0.301	0	0.501	1	0.589	1	0.658	1	0.747	1	0.787	1
Rangpur	2026	0.015	0	0.051	0	0.099	0	0.140	0	0.157	0	0.143	0	0.106	0	0.135	0	0.164	0	0.149	0	0.183	0	0.208	0
Satkhira	2026	0.093	0	0.081	0	0.073	0	0.117	0	0.239	0	0.469	0	0.661	1	0.745	1	0.796	1	0.742	1	0.761	1	0.787	1

Shariatpur	2026	0.723	1	0.759	1	0.787	1	0.839	1	0.923	1	0.958	1	0.970	1	0.979	1	0.976	1	0.976	1	0.987	1	0.990	1
Sherpur	2026	0.177	0	0.724	1	0.737	1	0.776	1	0.795	1	0.809	1	0.861	1	0.776	1	0.666	1	0.650	1	0.504	1	0.412	0
Sirajganj	2026	0.813	1	0.866	1	0.851	1	0.899	1	0.957	1	0.982	1	0.992	1	0.990	1	0.992	1	0.986	1	0.988	1	0.990	1
Sunamganj	2026	0.002	0	0.001	0	0.001	0	0.002	0	0.002	0	0.003	0	0.004	0	0.004	0	0.004	0	0.004	0	0.008	0	0.010	0
Sylhet	2026	0.006	0	0.011	0	0.011	0	0.005	0	0.004	0	0.005	0	0.004	0	0.002	0	0.001	0	0.001	0	0.001	0	0.001	0
Tangail	2026	0.264	0	0.276	0	0.379	0	0.669	1	0.878	1	0.973	1	0.990	1	0.987	1	0.990	1	0.986	1	0.981	1	0.982	1
Thakurgaon	2026	0.020	0	0.035	0	0.106	0	0.328	0	0.577	1	0.668	1	0.596	1	0.547	1	0.502	1	0.496	0	0.491	0	0.517	1

Note: 1: High allart; 0: no warning

References

1. DGHS. Dengue Press Releases. Directorate General of Health Services. Available online: https://old.dghs.gov.bd/index.php/bd/home/5200-daily-dengue-status-report (accessed on 25 November 2025).
2. 21.DGHS. Bangladesh National Dengue Prevention and Control Strategy (2024–2030). Directorate General of Health Services. 2024. Available online: https://dashboard.dghs.gov.bd/pages/index.php (accessed on 25 November 2025).
3. NASA. Earthdata Search. Available online: https://search.earthdata.nasa.gov/search (accessed on 25 November 2025).
4. BBS. Bangladesh Bureau of Statistics. Available online: http://nsds.bbs.gov.bd/en (accessed on 25 November 2025).
5. WorldBank. World Bank Group—International Development, Poverty and Sustainability. Available online: https://www.worldbank.org/ext/en/home (accessed on 25 November 2025).
6. WHO. Indicators Index. Available online: https://www.who.int/data/gho/data/indicators/indicators-index (accessed on 25 November 2025).
7. Xu X, Shrestha S, Gilani H, Gumma MK, Siddiqui BN, Jain AK. Dynamics and drivers of land use and land cover changes in Bangladesh. Reg Environ Change. 2020;20: 54. doi:10.1007/s10113-020-01650-5

8. Fattah MdA, Gupta SD, Farouque MdZ, Ghosh B, Morshed SR, Chakraborty T, et al. Spatiotemporal characterization of relative humidity trends and influence of climatic factors in Bangladesh. Heliyon. 2023;9: e19991. doi:10.1016/j.heliyon.2023.e19991
9. Azur MJ, Stuart EA, Frangakis C, Leaf PJ. Multiple imputation by chained equations: what is it and how does it work? Int J Methods Psychiatr Res. 2011;20: 40–49. doi:10.1002/mpr.329
10. Olaniran OR, Alzahrani ARR. Bayesian Random Forest with Multiple Imputation by Chain Equations for High-Dimensional Missing Data: A Simulation Study. Mathematics. 2025;13: 956. doi:10.3390/math13060956
11. Xu G, Zhu H, Lee JJ. Borrowing strength and borrowing index for Bayesian hierarchical models. Comput Stat Data Anal. 2020;144: 106901. doi:10.1016/j.csda.2019.106901
12. Arnold KF, Davies V, de Kamps M, Tennant PWG, Mbotwa J, Gilthorpe MS. Reflection on modern methods: generalized linear models for prognosis and intervention—theory, practice and implications for machine learning. Int J Epidemiol. 2020;49: 2074–2082. doi:10.1093/ije/dyaa049
13. G K, K P I, Hasin A J, M LFJ, Siluvai S, G K. Support Vector Machines: A Literature Review on Their Application in Analyzing Mass Data for Public Health. Cureus. 17: e77169. doi:10.7759/cureus.77169
14. Wiens M, Verone-Boyle A, Henscheid N, Podichetty JT, Burton J. A Tutorial and Use Case Example of the eXtreme Gradient Boosting (XGBoost) Artificial Intelligence Algorithm for Drug Development Applications. Clin Transl Sci. 2025;18: e70172. doi:10.1111/cts.70172
15. Rossi F, Conan-Guez B. Functional multi-layer perceptron: a non-linear tool for functional data analysis. Neural Netw. 2005;18: 45–60. doi:10.1016/j.neunet.2004.07.001
16. Krichen M, Mihoub A. Long Short-Term Memory Networks: A Comprehensive Survey. AI. 2025;6: 215. doi:10.3390/ai6090215
17. Sun M, Meng Q, Zhang L, Hu X, Lei X, Chen S, et al. Convolutional Long Short-Term Memory network for generating 100 m daily near-surface air temperature. Sci Data. 2025;12: 749. doi:10.1038/s41597-025-05032-6
18. Farooq Z, Rocklöv J, Wallin J, Abiri N, Sewe MO, Sjödin H, et al. Artificial intelligence to predict West Nile virus outbreaks with eco-climatic drivers. Lancet Reg Health - Eur. 2022;17: 100370. doi:10.1016/j.lanepe.2022.100370
19. Al-Selwi SM, Hassan MF, Abdulkadir SJ, Muneer A, Sumiea EH, Alqushaibi A, et al. RNN-LSTM: From applications to modeling techniques and beyond—Systematic review. J King Saud Univ - Comput Inf Sci. 2024;36: 102068. doi:10.1016/j.jksuci.2024.102068
20. Adeoye M, Didelot X, Spencer SEF. Bayesian spatio-temporal modelling for infectious disease outbreak detection. Epidemics. 2026;54: 100879. doi:10.1016/j.epidem.2025.100879
21. Coly S, Garrido M, Abrial D, Yao A-F. Bayesian hierarchical models for disease mapping applied to contagious pathologies. PLoS ONE. 2021;16: e0222898. doi:10.1371/journal.pone.0222898
22. Massoud EC, Lee HK, Terando A, Wehner M. Bayesian weighting of climate models based on climate sensitivity. Commun Earth Environ. 2023;4: 365. doi:10.1038/s43247-023-01009-8
23. Morris M, Wheeler-Martin K, Simpson D, Mooney SJ, Gelman A, DiMaggio C. Bayesian hierarchical spatial models: Implementing the Besag York Mollié model in stan. Spat Spatio-Temporal Epidemiol. 2019;31: 100301. doi:10.1016/j.sste.2019.100301
24. Flagg K, Hoegh A. The integrated nested Laplace approximation applied to spatial log-Gaussian Cox process models. J Appl Stat. 50: 1128–1151. doi:10.1080/02664763.2021.2023116
25. Chen Q, Yan M, Li J, Wang X. Optimal meso-granularity selection for classification based on Bayesian optimization. Knowl-Based Syst. 2025;318: 113552. doi:10.1016/j.knosys.2025.113552
26. Chien L-C, Yu H-L. Impact of meteorological factors on the spatiotemporal patterns of dengue fever incidence. Environ Int. 2014;73: 46–56. doi:10.1016/j.envint.2014.06.018
